# M‐CSF directs myeloid and NK cell differentiation to protect from CMV after hematopoietic cell transplantation

**DOI:** 10.15252/emmm.202317694

**Published:** 2023-08-28

**Authors:** Prashanth K Kandalla, Julien Subburayalu, Clément Cocita, Bérengère de Laval, Elena Tomasello, Johanna Iacono, Jessica Nitsche, Maria M Canali, Wilfried Cathou, Gilles Bessou, Noushin Mossadegh‐Keller, Caroline Huber, Guy Mouchiroud, Roland P Bourette, Marie‐France Grasset, Martin Bornhäuser, Sandrine Sarrazin, Marc Dalod, Michael H Sieweke

**Affiliations:** ^1^ Center for Regenerative Therapies Dresden (CRTD) Technical University Dresden Dresden Germany; ^2^ Aix Marseille University, CNRS, INSERM CIML Marseille France; ^3^ Department of Internal Medicine I University Hospital Carl Gustav Carus Dresden Dresden Germany; ^4^ Aix‐Marseille University, CNRS, INSERM CIML, Turing Center for Living Systems Marseille France; ^5^ Institut NeuroMyoGene, UMR CNRS 5310 INSERM Lyon France; ^6^ CNRS, INSERM, CHU Lille, University Lille UMR9020‐U1277 ‐ CANTHER – Cancer Heterogeneity Plasticity and Resistance to Therapies Lille France; ^7^ CNRS UMR5534 Claude Bernard Lyon 1 University Villeurbanne France; ^8^ National Center for Tumor Diseases (NCT), Dresden Dresden Germany

**Keywords:** CMV (prophylaxis), HCT, host‐directed therapy, M‐CSF (CSF‐1), NK cell, Immunology, Stem Cells & Regenerative Medicine

## Abstract

Therapies reconstituting autologous antiviral immunocompetence may represent an important prophylaxis and treatment for immunosuppressed individuals. Following hematopoietic cell transplantation (HCT), patients are susceptible to *Herpesviridae* including cytomegalovirus (CMV). We show in a murine model of HCT that macrophage colony‐stimulating factor (M‐CSF) promoted rapid antiviral activity and protection from viremia caused by murine CMV. M‐CSF given at transplantation stimulated sequential myeloid and natural killer (NK) cell differentiation culminating in increased NK cell numbers, production of granzyme B and interferon‐γ. This depended upon M‐CSF‐induced myelopoiesis leading to IL15Rα‐mediated presentation of IL‐15 on monocytes, augmented by type I interferons from plasmacytoid dendritic cells. Demonstrating relevance to human HCT, M‐CSF induced myelomonocytic IL15Rα expression and numbers of functional NK cells in G‐CSF‐mobilized hematopoietic stem and progenitor cells. Together, M‐CSF‐induced myelopoiesis triggered an integrated differentiation of myeloid and NK cells to protect HCT recipients from CMV. Thus, our results identify a rationale for the therapeutic use of M‐CSF to rapidly reconstitute antiviral activity in immunocompromised individuals, which may provide a general paradigm to boost innate antiviral immunocompetence using host‐directed therapies.

## Graphical abstract

*Herpesviridae* like CMV are a major cause of morbidity and mortality in patients after HCT. Therapies reconstituting the host's antiviral immunocompetence for prophylaxis and treatment are an unmet medical need since licensed therapies are either insufficiently effective or have severe side effects.

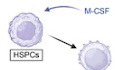

**The paper explained**

**Problem**

Viral infection or reactivation of lifelong dormant viruses in immunosuppressed patients accrues to a significant death toll. For example, reactivation of cytomegalovirus, an opportunistic virus of the *Herpesviridae* family, in patients that have undergone hematopoietic stem cell transplantation (HCT) is a major cause of acute mortality at the leukopenic phase when donor stem cell reconstitution has yet to occur. Unfortunately, licensed drugs against CMV are either insufficiently effective and rely on the viral replication machinery (letermovir) or have severe side effects including bone marrow toxicity and risk of posttreatment mutagenesis (ganciclovir). Hence, further alternative well‐tolerated host‐directed therapies are needed that rapidly confer broad‐spectrum antimicrobial competence in severely immunocompromised patients following HCT.

**Results**

The administration of M‐CSF around the time of transplantation revealed a novel mechanism to protect against lethal CMV infection in leukopenic mice following HCT. Here, M‐CSF stimulated hematopoietic stem cells to overcome leukopenia by committing to monopoiesis. We show that in mice M‐CSF engages critical host‐directed initial mechanisms to reconstitute antiviral immune responses after HCT, which are type I interferon and natural killer (NK) cell‐dependent. Mechanistically, NK cell‐mediated protection from lethal CMV infection arose from IL‐15 and type I interferon production by M‐CSF‐driven monopoiesis or plasmacytoid dendritic cell differentiation, respectively. Synergistically, these brought about NK cell differentiation and activity from recruited progenitors. As in mice, human G‐CSF‐mobilized stem and progenitor cells within PBMCs also responded to M‐CSF administration with elevated monopoiesis, IL‐15Rα expression, and functional NK cell generation. Lastly, we could show that M‐CSF did not confer any adverse effects on long‐term hematopoietic stem cell engraftment and hematopoietic lineage balance in the blood or acute post‐transplantation symptoms following allogenic HCT.

**Impact**

Overall, our preclinical results support the efficacy and feasibility of M‐CSF administration as a prophylactic host‐directed therapy that confers rapid protection from lethal CMV infection. Moreover, our data suggests a paradigm by which M‐CSF boosts innate antimicrobial immunocompetence, which may constitute a well‐tolerated general host‐directed therapeutic in states of severe immunosuppression.

## Introduction

The first months after hematopoietic cell transplantation (HCT) are characterized by profound immunosuppression, which leaves patients at high risk of viral infection or reactivation of common opportunistic viruses such as cytomegalovirus (CMV). The infection itself but also its subsequent treatment is associated with significant morbidity and mortality (Arber *et al*, 2003; Boeckh & Ljungman, 2009; Ahmed, 2011; El Chaer *et al*, 2016; Cho *et al*, 2019). Although vaccines against CMV are under development, they are not yet routinely available in the clinic (Plotkin, 2015). Moreover, antiviral treatments based on inhibition of viral replication are limited to specific viruses, can have significant bone marrow toxicity, and run the risk of variant development and breakthrough infections (Boeckh & Ljungman, 2009; Ahmed, 2011; El Chaer *et al*, 2016; Cho *et al*, 2019; Hill *et al*, 2021). Cell‐based therapies are still not widely on hand and associated with high costs (Kaeuferle *et al*, 2019). Biologics stimulating the patient's general antiviral immune response could therefore be a welcome alternative or complementary treatment option but are currently unavailable.

Myeloid cytokines can massively alter hematopoietic output (Boettcher & Manz, 2017) but G‐CSF, the major factor in clinical use, has no effect on antiviral immunity (Heuser *et al*, 2007). This appears likely because G‐CSF confers its activity only on late myeloid progenitors and mature myeloid cells that are not present early after HCT. By contrast, M‐CSF, another myeloid cytokine released during infections (Cheers & Stanley, 1988; Roth *et al*, 1997; Mossadegh‐Keller *et al*, 2013), and known to promote myelopoiesis (Motoyoshi, 1998; Metcalf, 2009; Ushach & Zlotnik, 2016), can directly act on hematopoietic stem and progenitor cells (HSPCs) to induce emergency myelopoiesis (Mossadegh‐Keller *et al*, 2013). Importantly, in concert with the myeloid transcription factor MafB, M‐CSF selectively controls asymmetric myeloid commitment division in HSPCs (Sarrazin *et al*, 2009; Sarrazin & Sieweke, 2011). Consequently, M‐CSF stimulates myeloid cell production without exhausting HSPCs (Mossadegh‐Keller *et al*, 2013; Kandalla *et al*, 2016). M‐CSF can protect against bacterial and fungal infections after HCT (Kandalla *et al*, 2016). However, antiviral activities of M‐CSF have not been reported yet.

Cytomegalovirus can lead to a diverse range of pathologies in immunocompromised humans (Griffiths *et al*, 2015; Griffiths & Reeves, 2021), and the closely related murine CMV (MCMV) has similar cellular tropism and kinetics (Krmpotic *et al*, 2003; Alexandre *et al*, 2014). The spleen is an early site for filtering blood‐borne virus and initiating immune responses, whereas the liver is a principal site of viral infection after its decline in the spleen (Hsu *et al*, 2009). Type I interferons (I‐IFNs) (Baranek *et al*, 2012), produced by plasmacytoid dendritic cells (pDCs) (Dalod *et al*, 2002; Zucchini *et al*, 2008), constitute a first line of defense against CMV with natural killer (NK) cells and cytotoxic T cells coming in as a critical second and third wave of the immune response that block viral replication by killing infected cells (Orange & Biron, 1996). Cytokines including IL‐12 and IL‐15 produced by conventional dendritic cells (cDCs) can indirectly contribute to viral defense by stimulating NK cell proliferation, activation, and effector function (Nguyen *et al*, 2002; Dalod *et al*, 2003; Baranek *et al*, 2012; Puttur *et al*, 2016). Other myeloid cells have been shown to have indirect and diverse roles in the response to CMV infection. Whereas Ly6C^−^CX3CR1^+^ patrolling monocytes act as carriers of CMV and can disseminate viral infection to distant organs throughout the body (Daley‐Bauer *et al*, 2014), Ly6C^+^CCR2^+^ inflammatory monocytes can activate NK and cytotoxic memory CD8^+^ T cells during microbial infection, including MCMV (Soudja *et al*, 2012; Rovis *et al*, 2016). Culture models proved that the ability of macrophages to resist MCMV infection depends on signaling mechanisms via I‐IFNs and type II IFNs (II‐IFNs) (Presti *et al*, 2001; Strobl *et al*, 2005; Kropp *et al*, 2011), which might also be important *in vivo*. Myeloid‐specific deletion of signal transducer and activator of transcription (STAT)1, a key transcription factor for mounting IFN responses, is also required for the early control of MCMV infection and spleen pathology but does not affect viral clearance (Gawish *et al*, 2019). Hence, the role of myeloid cells in MCMV infection appears multifaceted and complex.

Interestingly, in the myeloid STAT1 deletion model, the ability to combat early MCMV infection correlated with the ability to mount extramedullary hematopoiesis (Gawish *et al*, 2019). In this study we have specifically investigated the role of emergency hematopoiesis on MCMV infection under leukopenic conditions and report that M‐CSF‐induced myelopoiesis promotes rapid reconstitution of antiviral activity and protection from infection. Using a murine model of HCT and infection with lethal doses of MCMV, we observed that M‐CSF treatment prompted antiviral immunity resulting in substantially improved survival and pathogen clearance in mice. Dissecting the mechanism underlying this M‐CSF‐mediated protection against MCMV infection, we identified a multistep differentiation program in which M‐CSF‐induced myelopoiesis further stimulated NK cell differentiation and activation via IL‐15 and I‐IFN mediators. Lastly, we observed that M‐CSF also induced myelo‐monocytic differentiation from human G‐CSF‐mobilized HSPCs, enhanced IL15Rα expression on monocytes, and increased functional NK cells numbers.

## Results

### M‐CSF protects HCT recipients from CMV viremia and mortality

**Figure 1:**
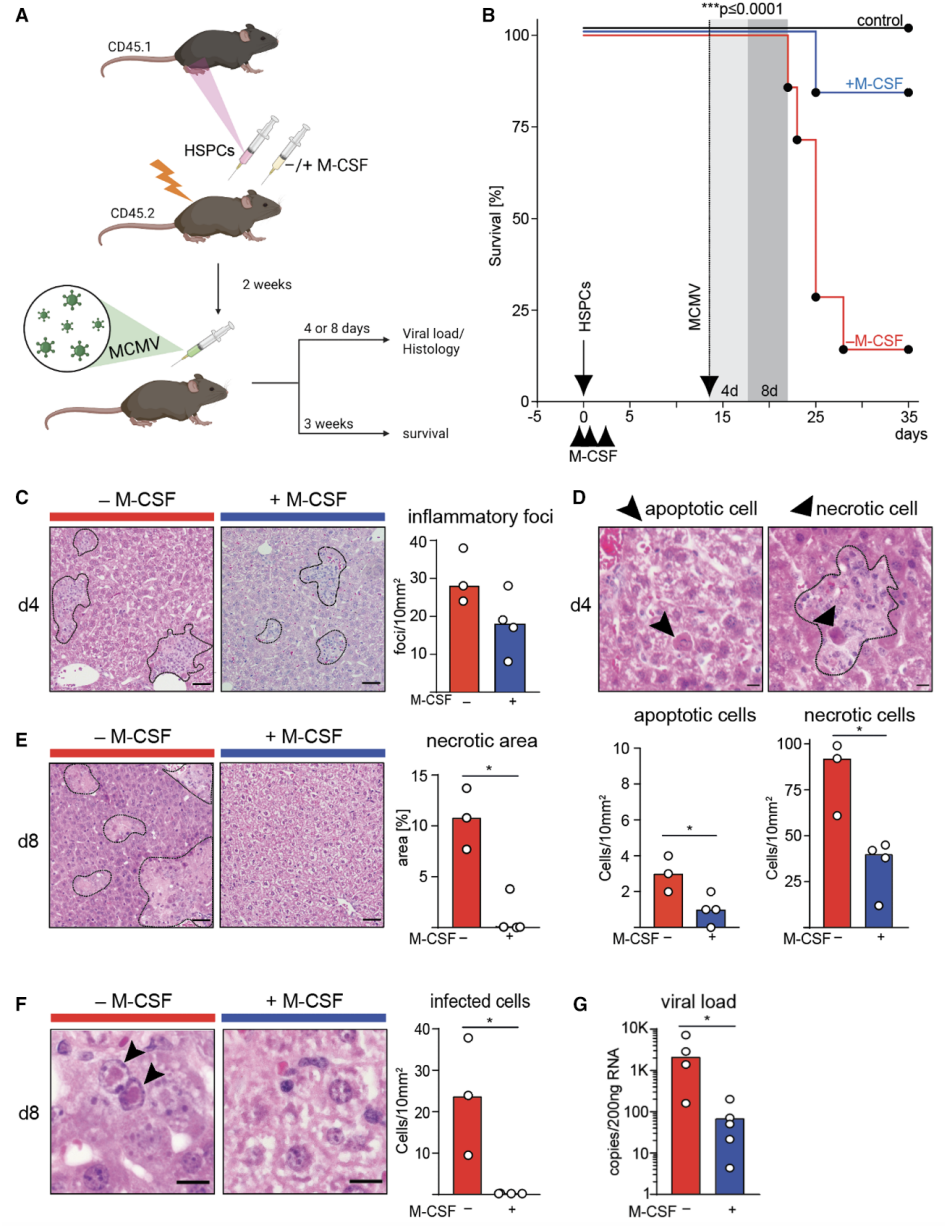
M‐CSF protects HSPC recipients from CMV viremia and mortality Leukopenia model to study MCMV viremia.Survival of mice after HSPC transplantation (arrow), MCMV infection (stippled arrow) and treatment (arrowheads) with control PBS (−M‐CSF; *n* = 7) or three doses of 10 μg mouse recombinant M‐CSF (+M‐CSF; *n* = 6). Transplanted, uninfected mice (*n* = 5) are shown as control.Histopathology of MCMV‐induced hepatitis. Assessment of inflammatory foci 4 days after infection of transplanted mice treated with M‐CSF or control PBS. Example of hematoxylin and eosin (H&E)‐staining and inflammatory foci (*n* = 4) (scale bar = 100 μm).Histopathology of MCMV‐induced hepatitis. Apoptotic (arrowheads) and necrotic (arrows) hepatocytes and quantification as median cell numbers per area (*n* = 4) (scale bar = 30 μm).Histopathology of MCMV‐induced hepatitis. Assessment of necrotic area 8 days after infection of transplanted mice (H&E). Percentage of affected areas (*n* = 4) (scale bar = 100 μm).Histological analysis of infected hepatocytes (H&E); quantification per area (*n* = 4) (scale bar = 30 μm).RT‐qPCR‐based quantitation of viral mRNA per 200 ng RNA (*n* = 5). Data information: ***P < 0.0001 by Mantel‐Cox test (B). *P < 0.05 by two‐tailed Mann–Whitney *U*‐test (C–G). All data are representative of at least two independent experiments. Source data are available online for this figure.

**Figure EV1:**
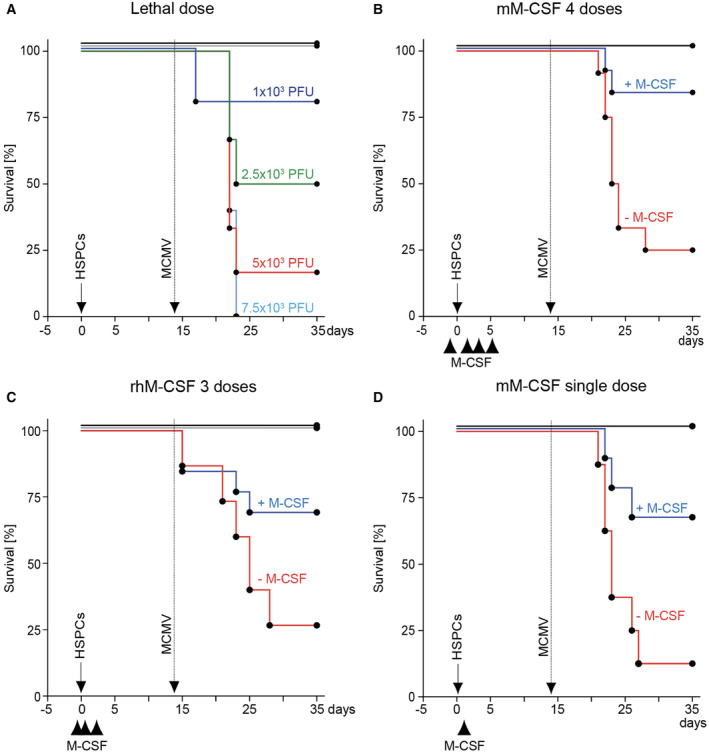
Titration of MCMV infection and M‐CSF treatments Survival of HSPC‐transplanted mice after MCMV infection. Two weeks after HCT, mice received MCMV intraperitoneally: 1,000 PFU (violet; *n* = 5), 2,500 PFU (green; *n* = 6), 5,000 PFU (red; *n* = 6) and 7,500 PFU (blue; *n* = 5). Transplantation controls (black; *n* = 10). Non‐irradiated, non‐transplanted mice with 7,500 PFU served as infection controls (brown; *n* = 6).Treatment with different doses and sources of M‐CSF. Survival of mice after infection (arrow), control (−M‐CSF, red) or M‐CSF (+M‐CSF, blue) or transplanted, uninfected controls (black). HSPC‐transplantation (solid arrow), MCMV infection (stippled) and different intravenous doses of control or M‐CSF. Treatment with 4 doses (−1 h, d+1, d+3, d+5) of 10 μg baculoviral‐expressed mouse M‐CSF (−M‐CSF, *n* = 12; +M‐CSF, *n* = 12; control, *n* = 5).Like B with 3 doses (−1 h, +5 h, +18 h) of 10 μg human recombinant M‐CSF (−M‐CSF, *n* = 15; +M‐CSF, *n* = 13; control, *n* = 5).Like B with a single dose (+5 h) of 10 μg baculoviral‐expressed mouse M‐CSF (−M‐CSF, *n* = 8; +M‐CSF, *n* = 9; control, *n* = 2). Data information: *P* < 0.0001 comparing +M‐CSF versus −M‐CSF as determined by Mantel‐Cox test (B–D). Source data are available online for this figure.

Cytomegalovirus infection/reactivation remains a perilous threat during immunosuppression (Arber *et al*, 2003; Locatelli *et al*, 2016; Ljungman *et al*, 2017). MCMV is a natural pathogen in mice that recapitulates patho‐mechanisms of human CMV infection (Krmpotic *et al*, 2003). To study the antiviral effects of M‐CSF on MCMV under leukopenic conditions, we used a murine HCT model (Kandalla *et al*, 2016). As shown in Fig 1A, mice received three injections of murine M‐CSF or PBS at the time of HCT and were infected 14 days later with MCMV doses accounting for 80–90% lethality in untreated transplant recipients (Fig EV1A). Survival rates significantly increased from 14.3 to 83.3% in M‐CSF‐treated mice (Fig 1B). Mice receiving four treatments of murine M‐CSF over several days (Fig EV1B) or human M‐CSF (Fig EV1C) both showed similar but not further improved survival rates. Accordingly, we used three treatments at the time of transplant as optimal condition throughout the study, although it should be stressed that a single M‐CSF treatment already improved survival rates to a similar extent (Fig EV1D).

Macrophage colony‐stimulating factor‐treated mice showed less severe liver injury with a proclivity for scarcer inflammatory foci (Fig 1C), a reduction of apoptotic or necrotic hepatocytes (Fig 1D), and decreased necrotic areas after MCMV infection (Fig 1E). M‐CSF‐treated mice also showed a decreased viral load as shown by reduced number of infected hepatocytes (Fig 1F) and viral RNA copy numbers (Fig 1G). Together, these results demonstrated that M‐CSF treatment protected HCT recipients from MCMV‐induced tissue damage and lethality.

### M‐CSF treatment increases NK cell abundance, differentiation, and activation

**Figure 2:**
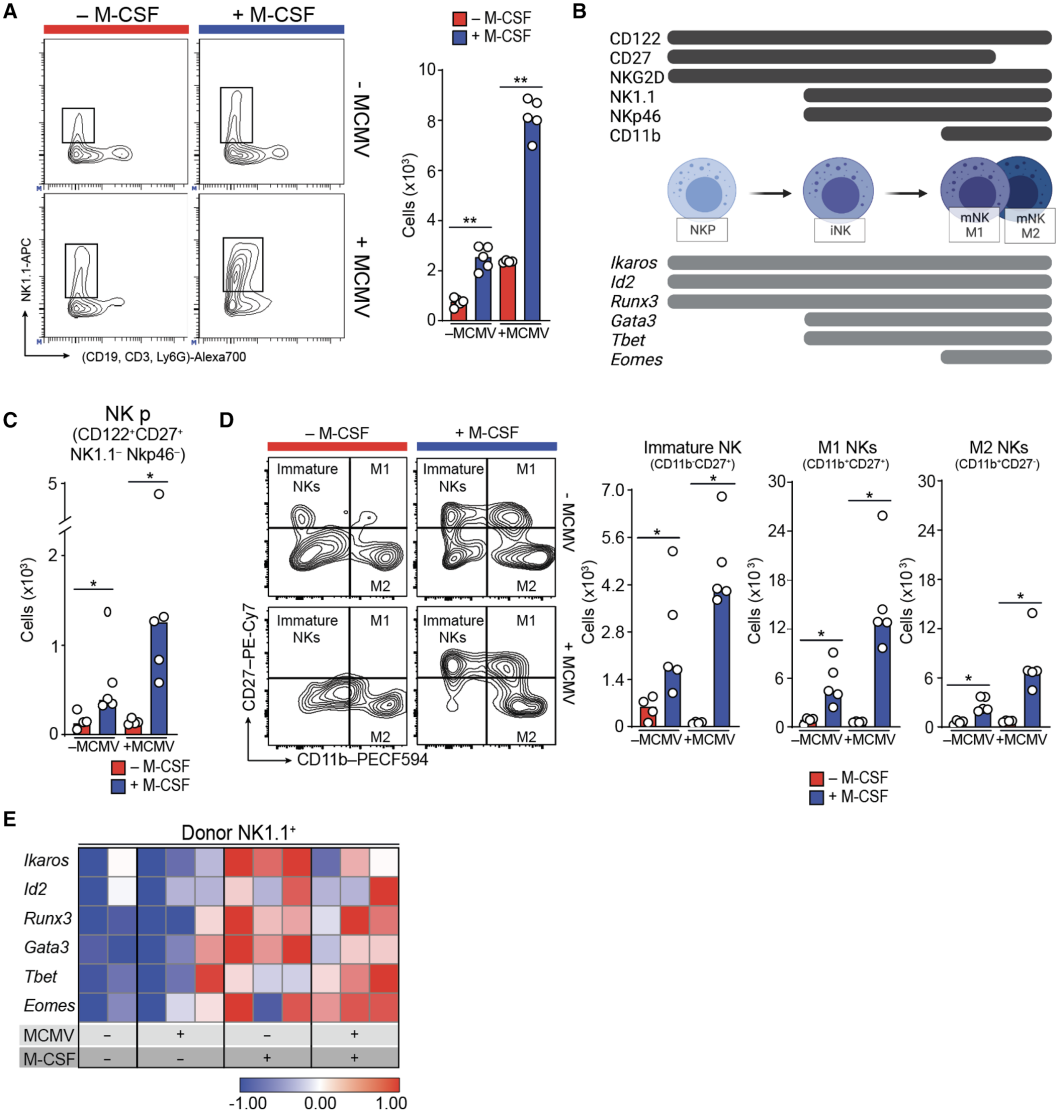
M‐CSF treatment increases NK cell production, differentiation, and activation Experimental set‐up as in Fig 1A. Analysis of spleen NK cell populations. Mice were MCMV‐ or mock‐infected (PBS control) 14 days after HCT (± M‐CSF support as indicated in Fig 1A). Analysis was performed 1.5 days after MCMV or mock infection. FACS examples and median of absolute number of total NK cells (CD19^−^CD3^−^Ly6G^−^NK1.1^+^) are shown (*n* = 5 mice per group, one independent experiment is shown but was confirmed twice).Markers specific to differentiation and maturation stages of NK cells used in this analysis are indicated.Median of absolute number of donor‐derived NK progenitor cells (CD122^+^CD27^+^NK1.1^−^Nkp46^−^CD45.1^+^) are displayed (*n* = 5 mice per group, one independent experiment is shown but was confirmed twice).FACS examples and median of absolute numbers of donor‐derived immature NK cells, donor‐derived M1 (CD11b^+^ CD27^+^) and M2 NK cells (CD11b^+^CD27^−^) are shown (*n* = 5 mice per group, one independent experiment is shown but was confirmed twice).Gene expression analysis of transcription factors expressed by NK cells in FACS‐sorted, donor‐derived NK1.1^+^ NK cells (definitions of Fig 2A) by nanofluidic Fluidigm array real‐time PCR. Data information: *P < 0.05, **P < 0.01 by two‐tailed Mann–Whitney *U*‐test. All data are representative of two independent experiments. Source data are available online for this figure.

**Figure EV2:**
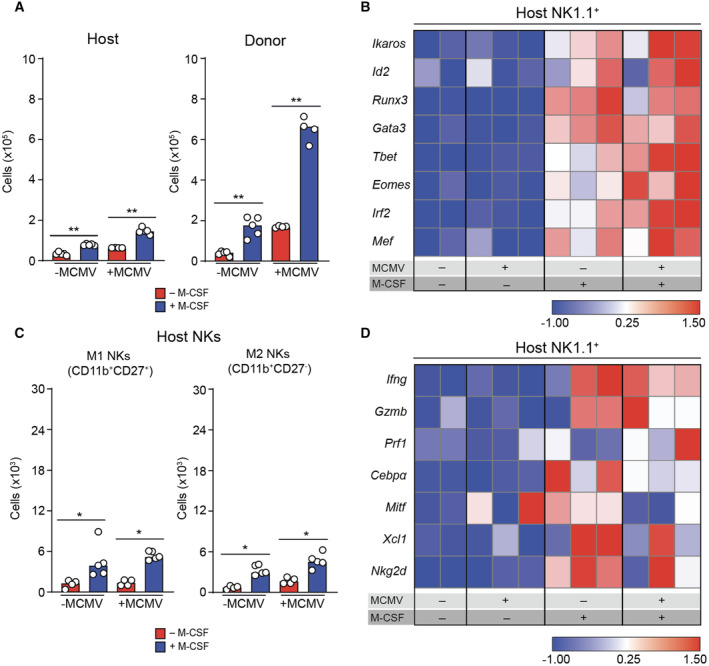
M‐CSF effect on NK cell production, maturation and differentiation in donor and recipient cells after hematopoietic cell transplantation Median of absolute numbers of (CD45.2^+^) recipient (left) and (CD45.1^+^) donor (right) NK cells (CD19^−^CD3^−^Ly6G^−^NK1.1^+^). **P < 0.01 by Mann–Whitney *U*‐test.Gene expression profiling of transcription factors measured by nanofluidic Fluidigm array RT‐qPCR of host‐derived NK cells, which were isolated from the spleens of control or M‐CSF‐treated recipient mice 1.5 days after MCMV of infection or time‐matched, mock‐infected, HSPC‐transplanted mice.Median of absolute numbers of host‐derived M1 NK cells (CD11b^+^CD27^+^) and host‐derived M2 NK cells (CD11b^+^CD27^−^) in the spleen of PBS control or M‐CSF‐treated recipient mice 1.5 days after MCMV or mock infection 14 days after HSPC transplantation. *P < 0.05 by Mann–Whitney *U*‐test.Gene expression profiling of host‐derived NK cells, which were isolated from the spleen of control or M‐CSF‐treated recipient mice 1.5 days after MCMV or mock infection for activation and maturation related factors by RT‐qPCR. Source data are available online for this figure.

Since NK cells are early antiviral effector cells, including during HCT (Ullah *et al*, 2016), we investigated whether M‐CSF treatment influenced NK cells. We observed an increase in NK cell numbers in the spleen two weeks after M‐CSF treatment both in uninfected mice and after infection (Fig 2A). Separate analysis of CD45.2^+^ recipient and CD45.1^+^ graft donor cells revealed that most of the NK cell increase arose from donor cells (Fig EV2A). Since M‐CSF is short‐lived (Koths, 1997), but increased NK cell numbers two weeks after application, we supposed the mechanism to act on NK cell progenitors. NK cell differentiation stages can be identified by differential expression of surface markers and transcription factors (Fig 2B) (Huntington *et al*, 2013; Vosshenrich & Di Santo, 2013; Serafini *et al*, 2015). NK cell progenitors express CD122, CD27, and NKG2D but not the mature markers NK1.1 and NKp46. We observed that M‐CSF increased the number of donor‐derived CD122^+^CD27^+^ NK cell progenitors both in uninfected and in infected mice (Fig 2C).

Consequently, we analyzed the NK cell maturation and differentiation status, which can be distinguished into CD11b^−^CD27^+^ immature, CD11b^+^CD27^+^ mature M1 and CD11b^+^CD27^−^ mature M2 NK cells (Fig 2B) (Kim *et al*, 2002; Hayakawa & Smyth, 2006; Chiossone *et al*, 2009). M‐CSF treatment increased both donor‐derived immature and mature M1 and M2 NK cells, particularly in infected mice (Fig 2D). A smaller increase of progenitor and mature cells was also observed for resident host NK cells (Fig EV2B).

This was further confirmed by gene expression analysis of stage‐specific transcription factors (Fig 2B). Fourteen days after M‐CSF‐supported HCT and after an additional 1.5 days of MCMV or mock infection, spleen NK1.1^+^ cells showed increased expression of the immature NK cell transcription factors *Ikaros*, *Id2*, *Runx3*, *Gata3*, and *Tbet* as well as the mature NK cell transcription factor *Eomes* after exposure to MCMV (Fig 2E). Similar observations were made for host‐derived NK cells (Fig EV2C). Whereas *Ikaros* and *Gata3* were more strongly induced by M‐CSF in uninfected mice, *Tbet* and *Eomes* were preferentially induced after infection (Fig 2E). Importantly, infection alone was insufficient for the observed inductions. Together, this indicated that M‐CSF leads to an increased number of NK cell progenitors and enhanced their differentiation along the NK cell lineage trajectory.

### NK cells execute M‐CSF‐stimulated antiviral immunity

**Figure 3:**
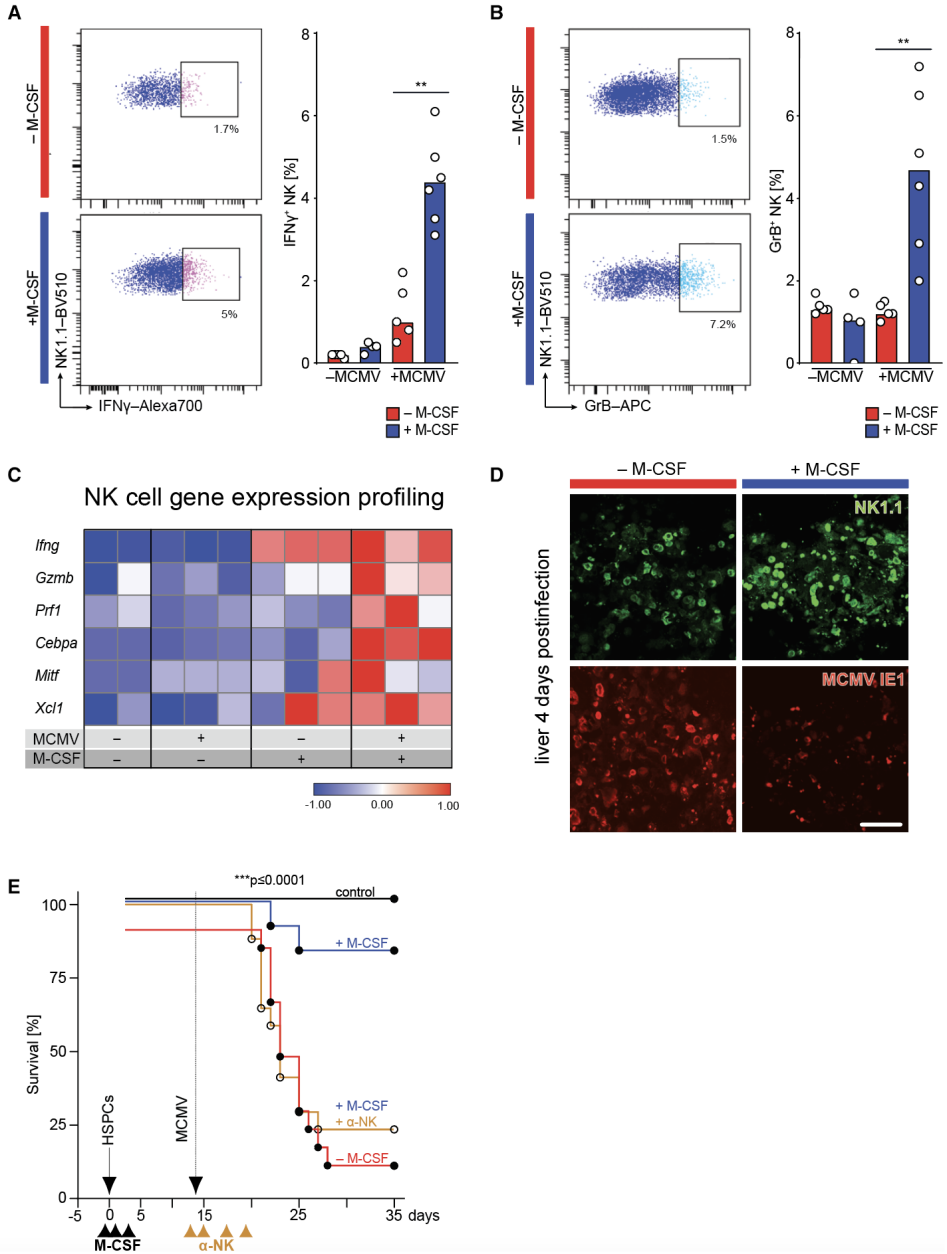
NK cell activity is required for the antiviral effect of M‐CSF Analysis performed 1.5 days (or 4 days in D) after MCMV or mock infection. NK cell activity in the spleen. FACS examples and median percentage of donor‐derived NK1.1^+^ NK cells producing IFNγ (*n* = 5–6 mice per group, two independent experiments).FACS examples and median percentage of donor‐derived NK1.1^+^ NK cells producing GrB (*n* = 5–6 mice per group, two independent experiments).Gene expression analysis of activation and maturation‐related factors in FACS‐sorted, donor‐derived NK1.1^+^ NK cells by RT‐qPCR.Immunofluorescence analyses with anti‐NK1.1 and anti‐MCMV IE1 antibodies in liver of HSPC‐transplanted and M‐CSF‐ or control mice 4 days after MCMV infection (scale bar = 100 μm).Assessment of M‐CSF‐mediated antiviral NK cell response. Survival of PBS control (−M‐CSF, *n* = 15), M‐CSF‐ and control IgG‐treated (*n* = 12), M‐CSF and anti‐NK1.1‐treated (*n* = 17) or transplanted, uninfected control mice (*n* = 4). Mice underwent HSPC‐transplantation (solid arrow), control PBS or M‐CSF‐treatment (black arrowheads) and were infected with MCMV (stippled arrow) as shown in Fig 1A and B. Repeated treatment with anti‐NK1.1 antibody (or control IgG) was done before and after infection (d−1, d1, d3, d5). Data information: **P < 0.01 by two‐tailed Mann–Whitney *U*‐test (A, B), ***P < 0.0001 by Mantel‐Cox test (E). All data are representative of two independent experiments. Source data are available online for this figure.

The major antiviral activity of NK cells is mediated by the production of inflammatory cytokines like IFNγ, and perforin (*PFR1*)‐dependent delivery of granzyme B (GrB) into infected cells (Bukowski *et al*, 1984). Interestingly, M‐CSF treatment increased the number of IFNγ‐ (Fig 3A) and GrB‐producing NK cells (Fig 3B) in infected mice in concert with enhanced mRNA levels for *IFNG*, *GZMB* and *PRF1* as well as maturation and activation genes (*CEBPA*, *MITF* and *XCL1*; Figs 3C and EV2D). Consistently, M‐CSF induced NK cell accumulation at infectious foci within the liver early after infection culminating in reduced numbers of MCMV‐infected cells (Fig 3D). To determine whether antiviral NK cell activity was required for the protective effect of M‐CSF, we depleted NK cells using anti‐NK1.1 antibodies in M‐CSF‐treated and MCMV‐infected HCT recipients (Fig 3E). NK cell depletion nearly abolished the increased survival of M‐CSF‐treated mice, demonstrating that a significant part of the protective effect of M‐CSF against viral lethality depended on NK cells.

### M‐CSF‐induced myelopoiesis is required for its antiviral effect

**Figure 4:**
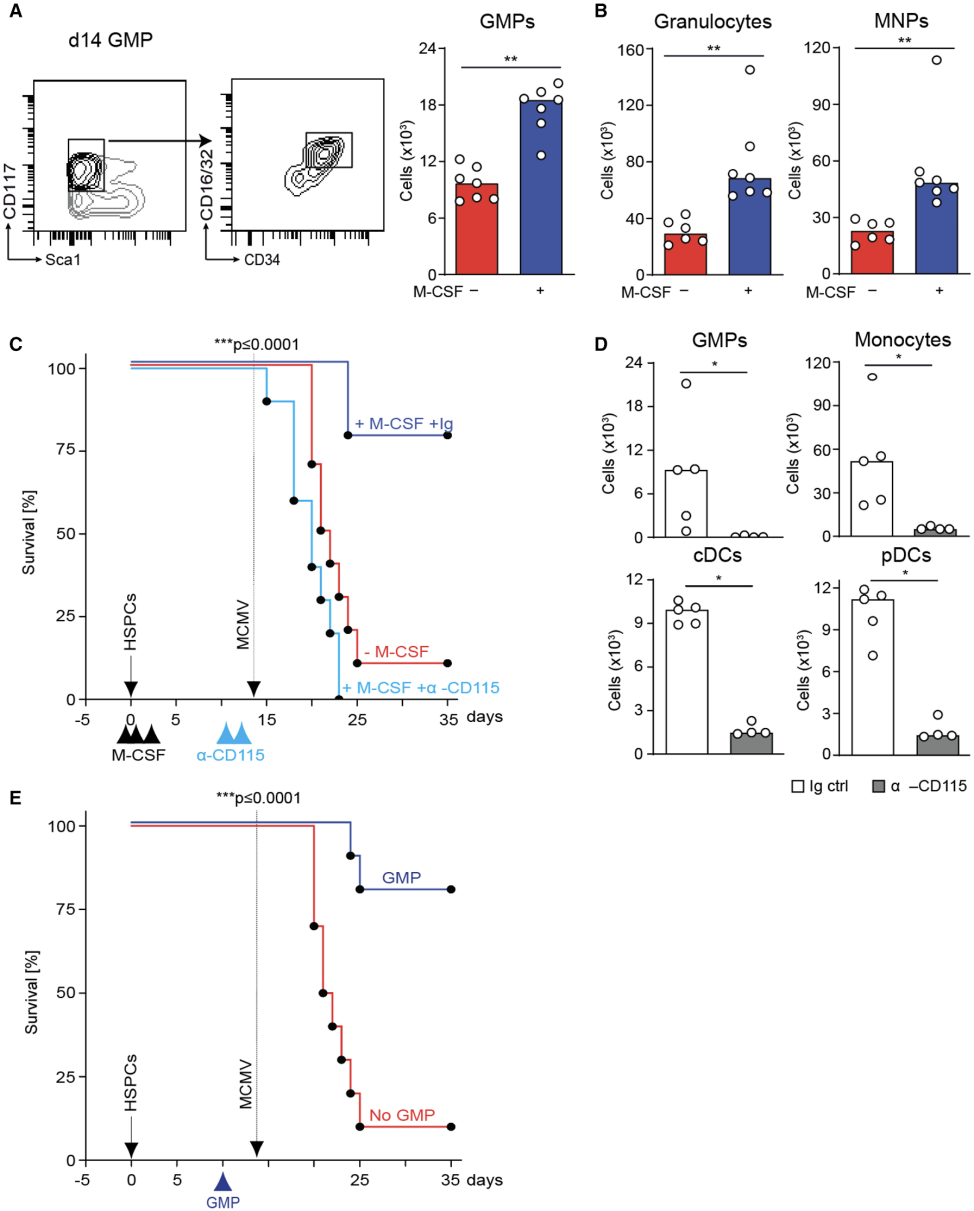
M‐CSF‐induced myelopoiesis is required for its antiviral effect Splenic GMPs of control or M‐CSF‐treated, uninfected mice 14 days after HCT.Splenic granulocytes (Ly6G^+^CD11b^+^) and mononuclear phagocytes (Ly6G^−^CD11b^+^) of control or M‐CSF‐treated, uninfected recipient mice 14 days after transplantation.Analysis of M‐CSF‐dependent myeloid cells for its antiviral effect. Survival curve of MCMV‐infected and PBS‐control‐treated (*n* = 10), M‐CSF and Ig‐control‐treated (*n* = 9) or M‐CSF and anti‐CD115 antibody‐treated mice (*n* = 10). After HCT (solid arrow), control or M‐CSF applied (black arrowheads). Infection with MCMV (stippled arrow) as in Fig 1A and B and treatment twice with anti‐CD115 antibody before infection (d−2, d−1).Splenic GMPs, monocytes, cDCs and pDCs of uninfected control‐ or anti‐CD115 antibody‐treated recipient mice 48 h after first treatment.GMP‐derived myeloid cells for antiviral activity. GMP transplantation with 50,000 cells on day 10 after HCT. Survival of MCMV‐infected control (no GMP, *n* = 10) or GMP‐transplanted mice (GMP, *n* = 10). Mice underwent HCT (HSPCs) (solid arrow), were infected with MCMV (stippled arrow) and GMP‐transplanted 10 days after HCT. Data information: ***P < 0.0001 by Mantel‐Cox test (C, E), *P < 0.05, **P < 0.01 by Mann–Whitney *U*‐test. All data are representative of two independent experiments. Source data are available online for this figure.

**Figure EV3:**
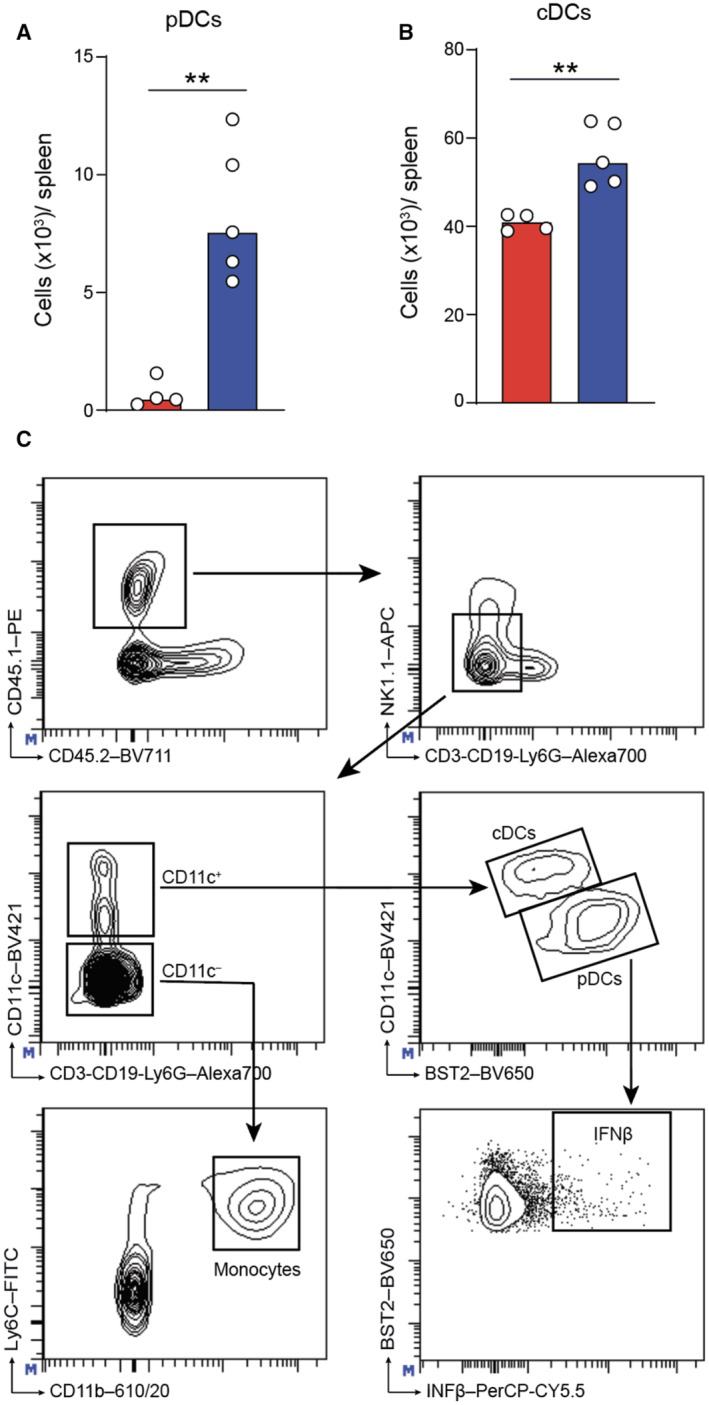
M‐CSF increases myelopoiesis of plasmacytoid dendritic cells and conventional dendritic cells Median of absolute numbers of donor‐derived spleen pDCs (Lin^−^CD11c^lo^BST2^high^) of mice treated with PBS control or M‐CSF 14 days after HCT and analyzed after an additional 1.5 days of MCMV or mock infection. **P < 0.01 by Mann–Whitney *U*‐test.Median of absolute numbers of cDCs (Lin^−^CD11c^+^BST2^−/low^) of mice treated with PBS control or M‐CSF 14 days after HCT and analyzed after an additional 1.5 days of MCMV or mock infection. **P < 0.01 by Mann–Whitney *U*‐test.Gating strategy for CD45.1^+^ monocytes, pDCs, IFN‐β and cDCs. Source data are available online for this figure.

Since M‐CSF has not been reported to act directly on the NK cell lineage, we investigated whether M‐CSF's effects on the myeloid lineage could indirectly impact on NK cell–mediated antiviral activity. M‐CSF treatment can increase donor myelopoiesis in HSPC‐transplanted mice (Mossadegh‐Keller *et al*, 2013; Kandalla *et al*, 2016). Accordingly, M‐CSF increased donor‐derived GMPs, granulocytes, mononuclear phagocytes (Fig 4A and B), pDCs, and cDCs (see Fig EV3A–C) two weeks after HCT. To determine whether this was relevant to the antiviral effect of M‐CSF, we used complementary loss‐ and gain‐of‐function approaches. We injected anti‐MCSFR/CD115 antibody 12 days after HCT, which selectively eliminates M‐CSF‐dependent myeloid cells (Tagliani *et al*, 2011). Myeloid cell depletion completely abolished the protective effect of M‐CSF treatment in MCMV‐infected HCT recipients (Fig 4C). Affirmatively, these mice showed reduced GMPs, monocytes, cDCs, and pDCs 48 h after anti‐CD115 myeloid depletion (Fig 4D). This indicated that myeloid cells were required for the M‐CSF‐dependent antiviral activity. For gain‐of‐function experiments, we transplanted GMPs into mice without M‐CSF support ten days after HCT (Fig 4A). GMP transplantation resulted in increased survival comparable to M‐CSF treatment (Fig 4E). Together, these experiments demonstrated that the antiviral activity of M‐CSF depends upon M‐CSF‐induced myelopoiesis.

### M‐CSF drives myeloid IL‐15 trans‐presentation to promote antiviral competence

**Figure 5:**
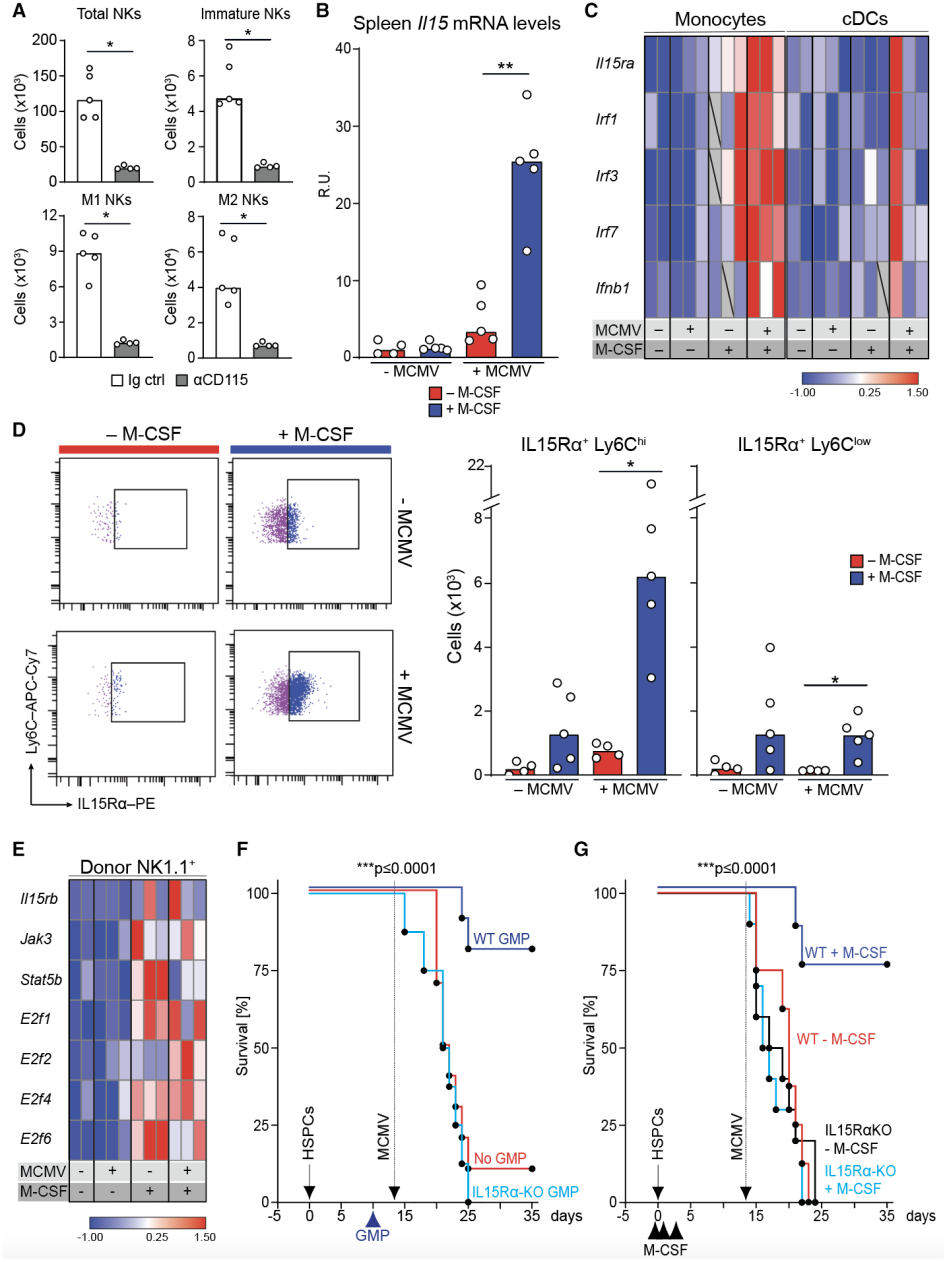
Myeloid IL‐15 trans‐presentation is required for the antiviral activity of M‐CSF Splenic NK1.1^+^, immature and mature NK cells of uninfected control or anti‐CD115‐treated mice 2 days after depletion and 14 days after HCT and M‐CSF treatment.Mice MCMV‐ or mock‐infected 14 days after HCT and analyzed 1.5 days after. *Il15* mRNA levels (RT‐qPCR).Mice MCMV‐ or mock‐infected 14 days after HCT and analyzed 1.5 days after. Sorted, donor‐derived monocytes and cDCs assessed by Fluidigm.Mice MCMV‐ or mock‐infected 14 days after HCT and analyzed 1.5 days after. Ly6C^hi^ monocytes (left), IL15Rα‐expressing, donor‐derived Ly6C^hi^ or Ly6C^low^ monocytes (right).Mice MCMV‐ or mock‐infected 14 days after HCT and analyzed 1.5 days after. Gene expression analysis of donor‐derived NK cells by Fluidigm.GMPs transplanted after HCT (D10). Survival of MCMV‐infected control (no GMP, *n* = 10), WT GMP (*n* = 10) or *IL15R*α‐KO GMP‐transplanted mice (IL15Rα‐KO GMP, *n* = 8). HCT (solid arrow), MCMV infection (stippled) and GMP‐transplantation (arrowhead).Survival of WT HCT, control‐treated (*n* = 8), WT HCT, M‐CSF‐treated (*n* = 8) or *IL15R*α‐KO HCT, control‐treated (*n* = 10) or *IL15R*α‐KO HCT, M‐CSF‐treated mice (*n* = 10). Mice transplanted with WT control HSPCs or *IL15R*α‐KO HSPCs (solid arrow), control or M‐CSF treatment (black arrowheads), and MCMV infection (stippled). Data information: *P < 0.05; **P < 0.01 by two‐tailed Mann–Whitney *U*‐test (A–D). ***P < 0.0001 by Mantel‐Cox test (F and G). Data are representative of two independent experiments. Source data are available online for this figure.

Since myelopoiesis and NK cell differentiation were required for the antiviral effect of M‐CSF, we hypothesized that M‐CSF‐induced myelopoiesis could indirectly affect NK cell differentiation and antiviral activity. Indeed, anti‐CD115‐mediated depletion of myeloid cells resulted in reduced immature and mature NK cells (Fig 5A). To identify myeloid signals that could affect NK cells, we first focused on IL‐15, a cytokine paramount for NK cell differentiation and effector functions (Nguyen *et al*, 2002; Budagian *et al*, 2006; Huntington *et al*, 2009; Boudreau *et al*, 2011). IL‐15 can be produced and trans‐presented by IL15Rα on myeloid cells (Lucas *et al*, 2007; Castillo *et al*, 2009; Huntington *et al*, 2009; Patidar *et al*, 2016) including during MCMV infection (Fehniger *et al*, 2007; Baranek *et al*, 2012; Ghilas *et al*, 2021). M‐CSF treatment resulted in swiftly increased IL‐15 mRNA levels in spleens after MCMV infection (Fig 5B). Since IL‐15 signaling requires trans‐presentation by the surface molecule IL15Rα (CD215) (Budagian *et al*, 2006; Castillo *et al*, 2009; Huntington *et al*, 2009), we analyzed the expression levels of IL15Rα in cDCs and monocytes, both capable of stimulating NK cells via IL‐15 (Lucas *et al*, 2007; Baranek *et al*, 2012; Domínguez‐Andrés *et al*, 2017). Both mRNA (Fig 5C) and surface protein analysis (Fig 5D) revealed that IL15Rα was induced in Ly6C^hi^ monocytes but only weakly in cDCs (Fig 5C) or Ly6C^lo^ monocytes (Fig 5D).

Next, we analyzed the effect of increased IL‐15 signaling from Ly6C^hi^ monocytes on the expression of IL‐15 response genes in NK target cells. Like IL‐15 signaling, which is engaged once the IL‐15/L15Rα complex binds to IL15Rβ on target cells (Huntington *et al*, 2009; Baranek *et al*, 2012; Patidar *et al*, 2016), M‐CSF treatment increased expression of the downstream genes *IL15RB* and of *STATB5*, *JAK3*, and *E2F1‐6* in NK cells (Fig 5E). To check whether IL‐15‐dependent myeloid cell to NK cell signaling was important for antiviral activity protecting HCT recipients from lethal MCMV infection, we compared *IL15RA*‐KO GMPs incapable of trans‐presenting IL‐15 with WT GMPs. We observed 80% survival in WT GMP‐transplanted mice after infection but no survival of *IL15RA*‐KO GMP‐transplanted mice (50,000 GMPs for each genotype), demonstrating that IL‐15 signaling from myeloid cells was required for NK cell support (Fig 5F). Furthermore, M‐CSF treatment resulted in no survival advantage in *IL15RA*‐KO HCT recipient mice and was comparable to untreated WT HCT mice (Fig 5G), indicating that IL‐15 signaling was acting downstream of M‐CSF. Together, our data demonstrate that myeloid‐derived IL‐15 signaling was required for the antiviral effect derived from M‐CSF‐induced myelopoiesis.

### M‐CSF‐induced I‐IFN production stimulates IL‐15‐dependent antiviral immunity

**Figure 6:**
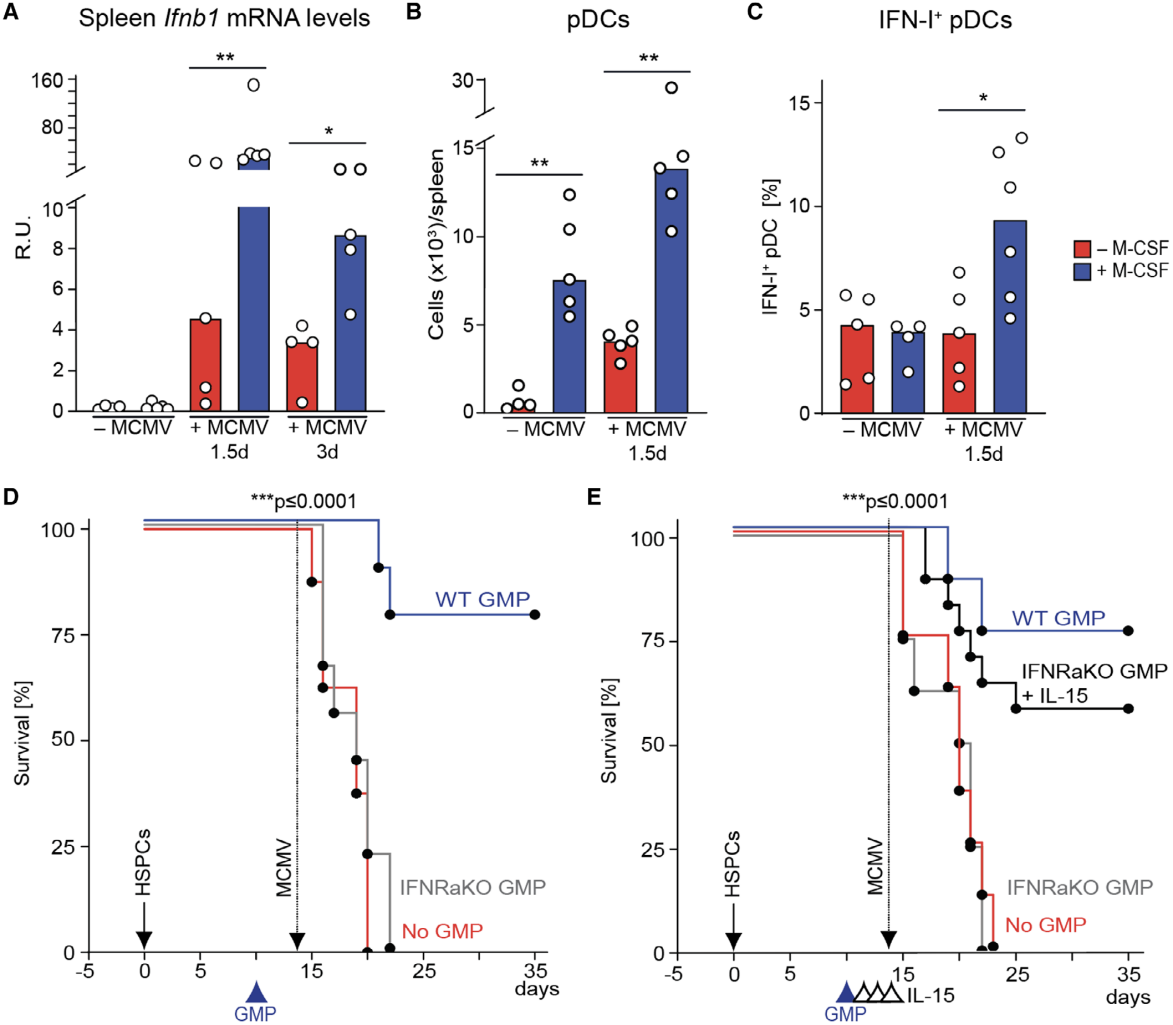
M‐CSF‐induced I‐IFN production stimulates IL‐15‐dependent antiviral effects MCMV‐ or mock‐infection 14 days after HCT. Analysis performed 1.5 days (or 3 days in A) after. Splenic Ifnb1 mRNA levels of control or M‐CSF‐treated mice (RT‐qPCR).Donor‐derived splenic Lin^−^CD11c^lo^BST2^hi^ pDCs.% IFN‐β^+^ pDCs.Survival of MCMV‐infected, no GMP control (*n* = 8), WT GMP (*n* = 9) or *Ifnar1*‐KO GMP‐transplanted mice (*n* = 9). HCT (solid arrow), MCMV infection (stippled) and GMP‐transplantation 10 days after HCT (arrowhead). *P* < 0.0001 by Mantel‐Cox test.Survival of MCMV‐infected, no GMP control (*n* = 8), WT GMP (*n* = 8) or *Ifnar1*‐KO GMP‐transplanted mice without (*n* = 8) or with IL‐15 rescue treatment (*n* = 16). HCT (solid arrow), MCMV infection (stippled), GMP transplantation 10 days after HCT (blue arrowhead) and treatment with 0.5 μg IL‐15 or control on days 12, 13 and 14 (black arrowheads). Data information: **P < 0.01, *P < 0.05 by Mann–Whitney *U*‐test (A–D). ***P < 0.0001 by Mantel‐Cox test (E and F). All data are representative of two independent experiments. Source data are available online for this figure.

Type I interferons contribute to the early antiviral immune response preceding NK cell activation (Degli‐Esposti & Smyth, 2005; Liu *et al*, 2005; Baranek *et al*, 2012; Alexandre *et al*, 2014; Cocita *et al*, 2015) and thus, may constitute a rapid response mechanism that could prevent fatal viremia during leukopenia after HCT. MCMV infection was shown to increase *IFNB1* mRNA in the spleen (Dalod *et al*, 2002; Zucchini *et al*, 2008). Consistently, we found enhanced *IFNB1* mRNA levels in the spleen swiftly after MCMV infection, which were strongly further augmented with M‐CSF treatment (Fig 6A). During MCMV infection, I‐IFNs are predominantly produced by pDCs. We observed that pDC numbers (Fig 6B) and I‐IFN‐producing pDCs (Fig 6C) were increased in the spleen of M‐CSF‐treated mice 14 days after HCT, particularly after MCMV infection. Monocytes also showed a strongly increased expression of *IFNB1* and upstream transcription factors of the IRF family (Fig 5C). Together, this supported the notion that M‐CSF treatment increased I‐IFN levels during MCMV infection of HCT recipients by promoting a faster reconstitution of I‐IFN‐producing monocytes and pDCs. This also agrees with the observation that M‐CSF‐driven myelopoiesis can also stimulate pDC development (Fancke *et al*, 2008). The observed effects of both loss‐ and gain‐of‐function experiments targeted at myeloid cells (Fig 4C and D) or transplantation of GMPs, which also give rise to pDCs, thus support the notion of I‐IFNs also contributing to antiviral immunity upon M‐CSF administration after HCT.

Beyond its direct antiviral effects on infected cells, I‐IFNs can also indirectly affect the antiviral immune response by activating NK cells or by stimulating IL‐15 production in myeloid cells (Nguyen *et al*, 2002; Degli‐Esposti & Smyth, 2005; Baranek *et al*, 2012). To investigate the relative importance of I‐IFNs on myeloid cells, we injected *IFNAR1*‐KO or WT GMPs at day 10 after HCT. *IFNAR1* deficiency abolished the protective effect of GMP transplantation (Fig 6D), indicating that I‐IFN stimulation of myeloid cells was required for their antiviral effect. IL‐15 treatment prior to infection could partially restore the deficiency of *IFNAR1*‐KO GMPs (Fig 6E), indicating the importance of I‐IFN induction of IL‐15 production in myeloid cells. Together, this suggested that the antiviral activity of I‐IFNs was mainly due to its effect on the identified myeloid and NK cell differentiation program rather than a direct effect on infected cells.

### M‐CSF recapitulates its effects in human G‐CSF‐mobilized PBMCs

**Figure 7:**
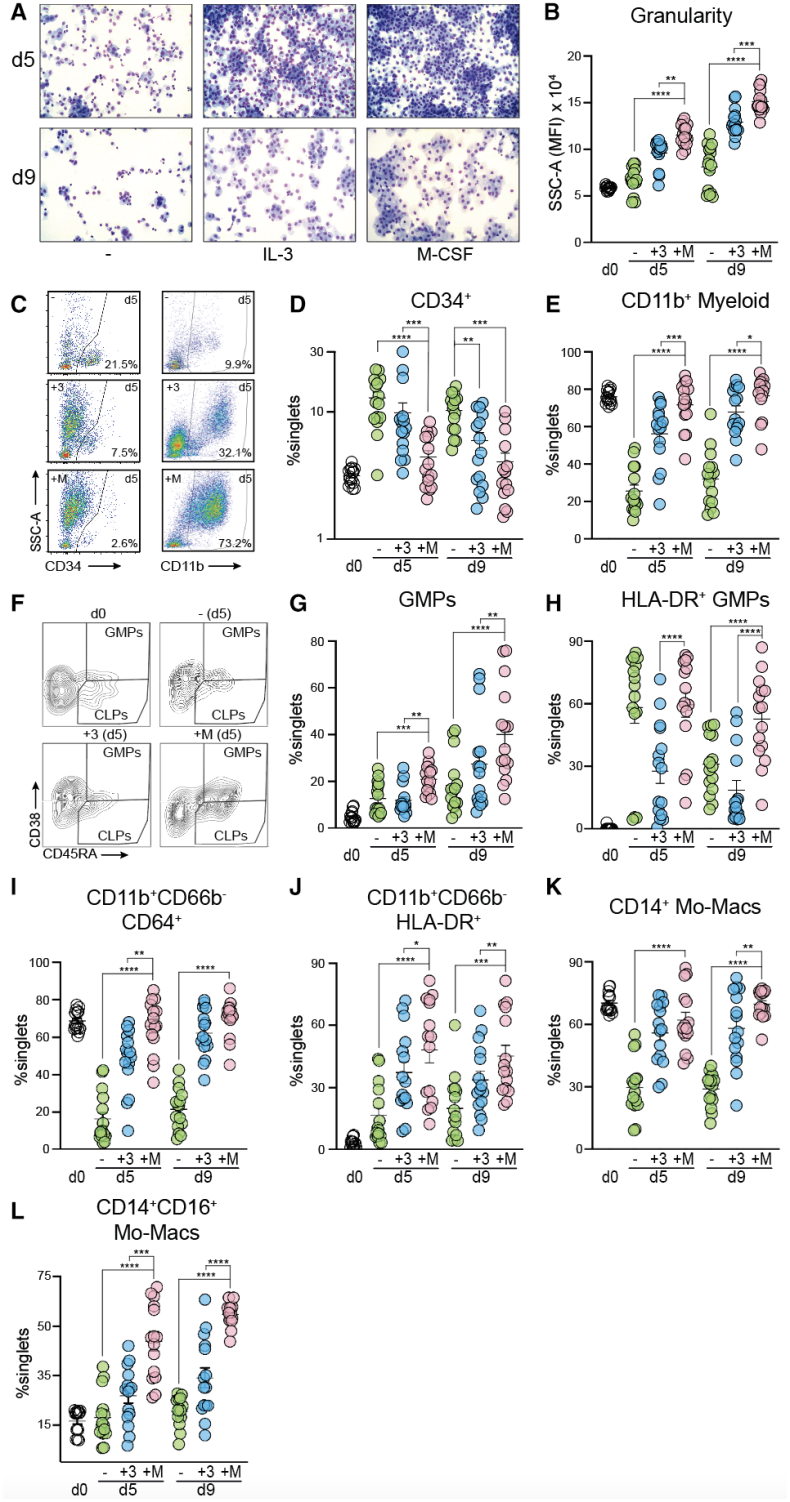
M‐CSF supports terminal differentiation of monocyte‐derived macrophages from human G‐CSF‐mobilized PBMCs Cytospins at days 5 or 9 after *in vitro* differentiation without myelopoiesis‐inducing cytokines (−), or with IL‐3 (+3) or M‐CSF (+M) (modified Giemsa). Images were acquired with a Zeiss AX10 benchtop microscope at 40× magnification.Median fluorescence intensity of SSC‐A (granularity) at seeding (d0), or after *in vitro* cytokine treatment without myelopoiesis‐inducing cytokines (−, green circle), with IL‐3 (+3, blue circle) or with M‐CSF (+M, salmon circle) (data shown in technical triplicates from five biological donors).Pseudocolor scatterplots of G‐CSF‐mobilized PBMCs after *in vitro* cytokine treatment shown for CD34 (left) and CD11b expression (right) on the *x*‐axis and SSC‐A on the *y*‐axis without myelopoiesis‐inducing cytokines (−, top row), with IL‐3 (+3, middle row) or with M‐CSF (+M, bottom row).Frequency of CD34^+^ HSPCs at seeding (d0), or after *in vitro* cytokine treatment without myelopoiesis‐inducing cytokines (−, green circle), with IL‐3 (+3, blue circle) or with M‐CSF (+M, salmon circle) (data shown in technical triplicates from five biological donors).Frequency of CD11b^+^ myeloid cells at seeding (d0), or after *in vitro* cytokine treatment without myelopoiesis‐inducing cytokines (−, green circle), with IL‐3 (+3, blue circle) or with M‐CSF (+M, salmon circle) (data shown in technical triplicates from five biological donors).Contour plots of HSPC populations comprising of CLPs (CD34^+^CD45RA^+^CD38^−^) or GMPs (CD34^+^CD45RA^+^CD38^+^) upon selection from G‐CSF‐mobilized PBMCs (d0) or after *in vitro* cytokine treatment at day 5: without myelopoiesis‐inducing cytokines (−) versus IL‐3 (+3) versus M‐CSF (+M).Frequency of GMPs at seeding (d0, empty circle) or without myelopoiesis‐inducing cytokine treatment (−, green circle), with IL‐3 (+3, blue circle) or with M‐CSF (+M, salmon circle) (data shown in technical triplicates from five biological donors).Frequency of HLA‐DR^+^ GMPs at seeding (d0, empty circle) or without myelopoiesis‐inducing cytokine treatment (−, green circle), with IL‐3 (+3, blue circle) or with M‐CSF (+M, salmon circle) (data shown in technical triplicates from five biological donors).M‐CSF‐stimulated differentiation of monocyte‐derived macrophages (Mo‐Macs, CD11b^+^CD66b^−^CD64^+^) (data shown in technical triplicates from five biological donors).M‐CSF‐stimulated differentiation of monocyte‐derived macrophages (Mo‐Macs, CD11b^+^CD66b^−^HLA‐DR^+^) (data shown in technical triplicates from five biological donors).Frequency of CD14^+^ cells at seeding (d0), or after *in vitro* cytokine treatment without myelopoiesis‐inducing cytokines (−, green circle), with IL‐3 (+3, blue circle) or with M‐CSF (+M, salmon circle) (data shown in technical triplicates from five biological donors).M‐CSF‐stimulated differentiation of monocyte‐derived macrophages (Mo‐Macs, CD14^+^CD16^+^) (data shown in technical triplicates from five biological donors). Data information: Data are illustrated as mean ± SEM. A ratio‐paired *t*‐test was used. *P < 0.05, **P < 0.01, ***P < 0.001, ****P < 0.0001. Data are representative of five independent experiments. Source data are available online for this figure.

**Figure EV4:**
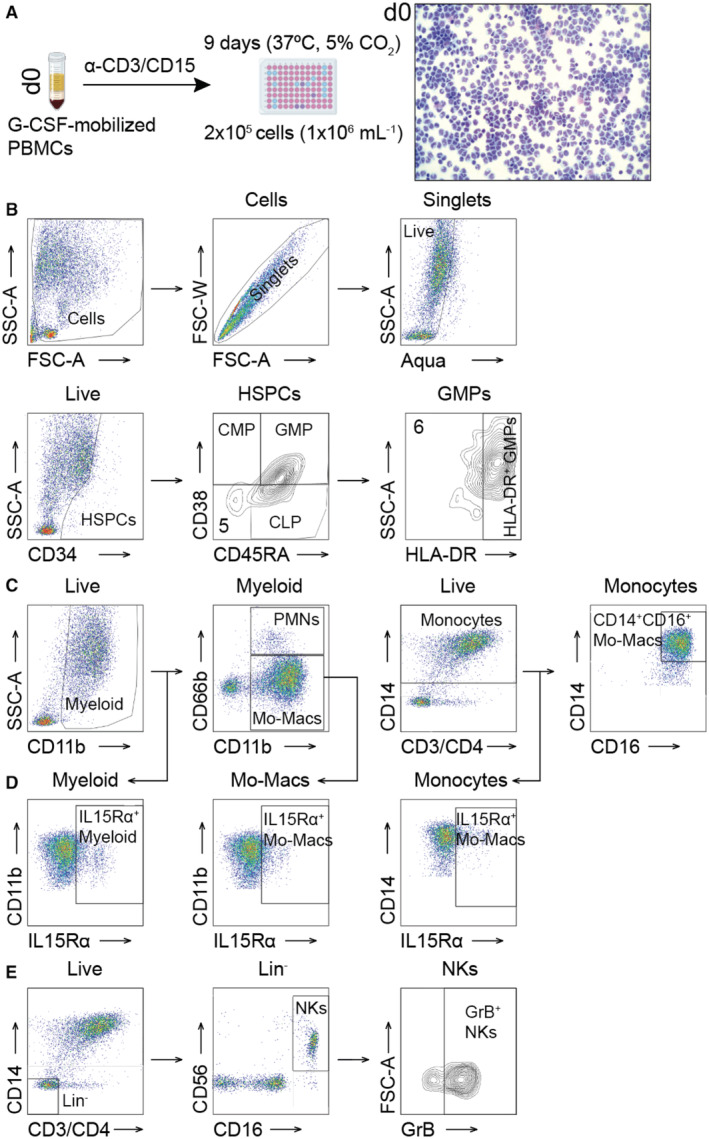
Gating strategy for M‐CSF‐driven myelopoiesis in G‐CSF‐mobilized human PBMCs, IL15Rα expression and NK cell frequency and activity Workflow for G‐CSF‐mobilized leukapheresis samplesGating strategy for flow cytometric assessment.“Live” singlets assessed for CD11b (“Myeloid” cells): polymorphonuclear neutrophils (PMNs, CD11b^+^CD66b^+^) and CD11b^+^CD66b^−^ “Monocytic” cells. The “Monocytes” (CD14^+^) gate permitted identification of CD14^+^CD16^+^ monocyte‐derived macrophages. At baseline (d0), this gating strategy was used to identify classical monocytes (CMs, CD14^+^CD16^−^), intermediate monocytes (IMs, CD14^+^CD16^+^) or non‐classical monocytes (NCMs, CD14^−^CD16^+^) accordingly.IL15Rα expression measured on myeloid, monocytic cells, or monocytes identified in (C).The gating strategy of OMIP‐027 with minor modifications by gating on the Lin^−^ cells from the dot plot shown in (C). Source data are available online for this figure.

To determine whether M‐CSF could affect myeloid and NK cell differentiation in human HSPCs, we assayed its impact on myelopoiesis, IL15Rα expression, NK cell numbers and functional competence in HSPC‐enriched PBMCs from G‐CSF‐mobilized stem cell donors (G‐PBMCs). *In vitro* differentiation from G‐PBMCs was established in the presence of stem cell factor (SCF) alone or in combination with the multi‐lineage myeloid cytokine IL‐3 or with M‐CSF, respectively (protocol Fig EV4A and the gating strategy used Fig EV4B–E). Both IL‐3 and particularly M‐CSF fostered myelopoiesis, yielding increasing proportions of cells with macrophage morphology (Fig 7A) and increased granularity (Fig 7B) over time. Supporting the notion that M‐CSF induced myelopoiesis, we found a faster and stronger reduction of CD34^+^ progenitors (Fig 7C and D) and a concomitant increase of CD11b^+^ myeloid cells for M‐CSF conditions (Fig 7C and E). Consistent with a faster myeloid commitment in the presence of M‐CSF, we also found increased numbers of GMPs (Fig 7F and G), in particular HLA‐DR^+^ mature GMPs (Fig 7H) (Sengupta *et al*, 1988; Lachmann *et al*, 2015). Beyond GMPs, the enhanced and accelerated frequencies of CD11b^+^ myeloid cells after M‐CSF treatment were mainly due to CD11b^+^CD66b^−^ monocytic cells rather than CD11b^+^CD66b^+^ granulocytes, which displayed differentiation commitment to monocyte‐derived macrophages (Mo‐Macs) as observed by increased expression of CD64 (Fig 7I) and HLA‐DR (Fig 7J) in CD11b^+^CD66b^−^ cells. This was further confirmed by enhanced expression of CD14^+^ (Fig 7K) at days 5 and 9, which after M‐CSF treatment, showed increased co‐expression of CD16 (Fig 7L). Together, these data indicated that M‐CSF also resulted in increased monopoiesis with a subsequent differentiation to Mo‐Macs in human HSPCs.

**Figure 8:**
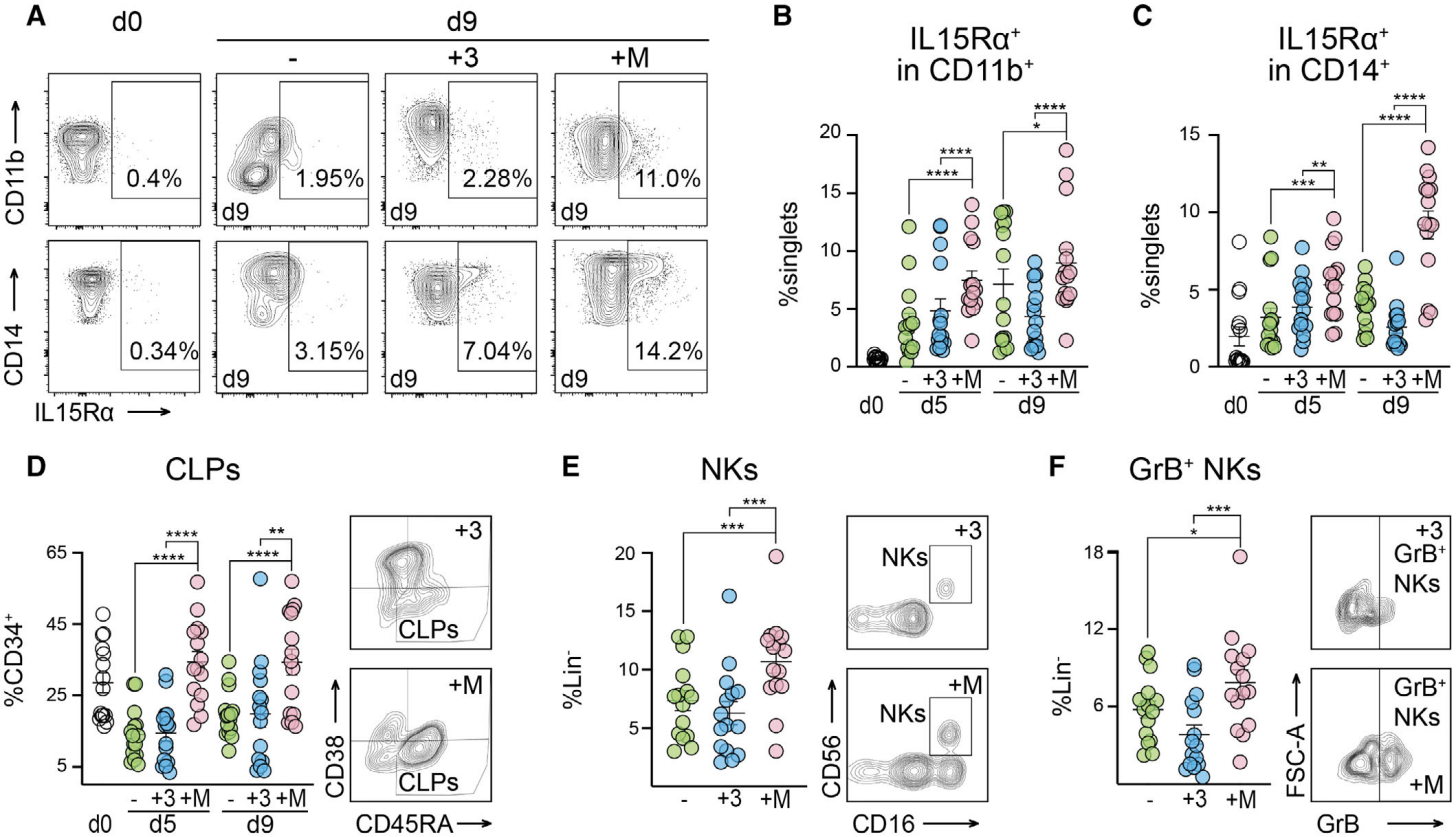
M‐CSF‐driven myelopoiesis induces IL15Rα expression by monocyte‐derived macrophages and supports NK cell viability and cytokine competence in human G‐CSF‐mobilized PBMCs Contour plots of IL15Rα expression by CD11b^+^ and CD14^+^ Mo‐Macs.Quantification of IL15Rα expression in CD11b^+^CD66b^−^ Mo‐Macs (data shown in technical triplicates from five biological donors).Quantification of IL15Rα expression in CD14^+^ Mo‐Macs (data shown in technical triplicates from five biological donors).Quantification of CLPs with representative contour plots of CLP enrichment after M‐CSF treatment (data shown in technical triplicates from five biological donors).M‐CSF supports NK cells (NKs, SSC‐A^low^Lin^−^CD56^+^CD16^+^) compared to − and +3 with representative contour plots of NKs comparing +3 with +M treatment (data shown in technical triplicates from five biological donors).M‐CSF enhances Granzyme B (GrB) production in NKs. Representative contour plots of GrB^+^ NKs after +3 or +M treatment are shown (data shown in technical triplicates from five biological donors). Data information: Data are illustrated as mean ± SEM. A ratio‐paired *t*‐test was used. *P < 0.1, **P < 0.05, ***P < 0.01, ****P < 0.001. Data are representative of five independent experiments. Source data are available online for this figure.

As observed in murine cells, M‐CSF treatment also resulted in enhanced IL15Rα expression on CD11b^+^ myeloid cells and CD14^+^ Mo‐Macs (Fig 8A) with increasing levels during differentiation on CD11b^+^CD66b^−^ or CD14^+^ Mo‐Macs (Fig 8B and C). Together, this indicated that M‐CSF treatment also enhanced IL‐15 presentation on human Mo‐Macs.

Finally, we further queried the effect of M‐CSF‐driven myelopoiesis and IL15Rα signaling in human G‐PBMCs on functional NK cell differentiation. We first analyzed CLPs, which encompass NK cell progenitors (Grzywacz *et al*, 2011). Interestingly, CLPs were enriched in M‐CSF‐treated G‐PBMCs, both at days 5 and 9 (Fig 8D). Although the culture regime lacked exogenous IL‐2, IL‐15 or IL‐21 and thus was not ideal for NK cell differentiation and survival, M‐CSF‐driven myelopoiesis resulted in significantly more NK cells (SSC‐A^low^Lin^−^CD56^+^CD16^+^) at day 9 of culture (Fig 8E). In line with the findings in murine cells, M‐CSF treatment also increased the numbers of GrB‐expressing NKs (Fig 8F) significantly compared to IL‐3‐driven myelopoiesis on day 9 of *in vitro* culture, indicating that in contrast to IL‐3, M‐CSF treatment specifically stimulated functionally competent NK cell production also in human G‐PBMCs.

Together, these findings indicated that the coordinated myeloid‐driven NK cell differentiation and activation program initiated by M‐CSF‐mediated myelopoiesis in mice was translatable to the human context and was thus directly relevant for clinical conditions of HCT.

### No adverse events of M‐CSF after allogeneic HCT

**Figure EV5:**
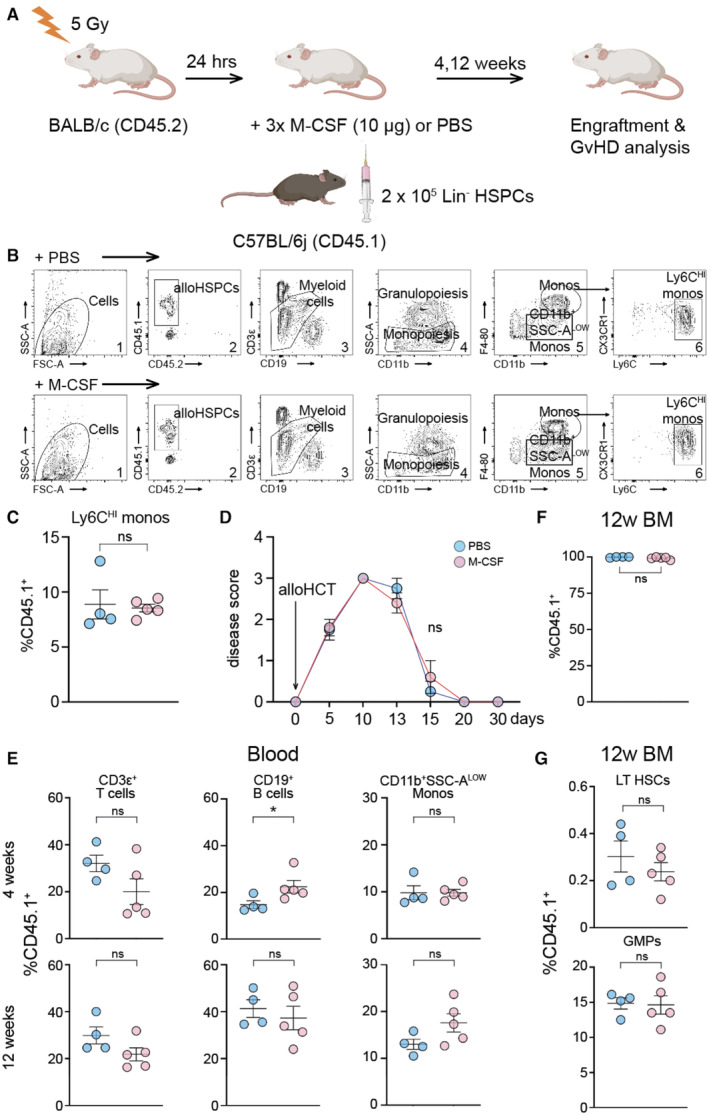
M‐CSF does neither confer adverse effects on tri‐lineage long‐term engraftment nor on GvHD after allogeneic hematopoietic cell transplantation The protocol for allogeneic hematopoietic stem cell transplantations (alloHCT) between BALB/c CD45.2^+^ recipient and C57BL/6j CD45.1^+^ donor mice. Before (1 h) or after (5 h, 20 h) alloHCT with 2 × 10^5^ lineage negative (Lin^−^) hematopoietic stem and progenitor cells (HSPCs), mice received PBS or 10 μg baculoviral‐expressed human M‐CSF.Engraftment of CD45.1^+^ cells was assessed at 4 and 12 weeks after alloHCT using the gating strategy by Alexander *et al* (2014).Quantification of inflammatory Ly6C^HI^ CD11b^+^F4/80^+^ monocytes (monos) of CD45.1^+^ cells.Disease scoring was applied as published by Lai *et al* (2012).Tri‐lineage engraftment (CD3ε^+^ T cells, CD19^+^ B cells, CD11b^+^SSC‐A^LOW^ monocytes) at 4 and 12 weeks post‐HCT in the blood.CD45.1^+^ cells in the bone marrow (BM) 12 weeks after alloHCT.Percentage of HSCs (KSL Flt3^−^CD150^+^CD48^−^) and GMPs in CD45.1^+^ lineage negative BM cells 12 weeks after alloHCT. Data information: The data are illustrated as mean ± SEM. The Mann–Whitney *U*‐test was used to test for statistical significance between PBS‐treated (*n* = 4) or M‐CSF‐treated allografted mice (*n* = 5). *P < 0.05, ns = not significant. Source data are available online for this figure.

Next, we analyzed the effects of M‐CSF on engraftment and recovery of transplant recipients in an allogeneic HCT model (Fig EV5A). Following allogeneic HCT and assessment according to previous reports (Alexander *et al*, 2014), we did not find any differences in the frequency of C57BL/6j CD45.1^+^ donor HSPC‐derived CD11b^+^F4/80^+^ monocytes or inflammatory Ly6C^HI^ monocytes after M‐CSF treatment (Fig EV5B and C). Furthermore, disease scoring (Lai *et al*, 2012) showed no statistical difference between mice treated with M‐CSF or PBS, going to the lowest possible score as early as 20 days following allogeneic HCT for both conditions (Fig EV5D). All mice survived M‐CSF treatment and vehicle control after allogeneic HCT. We further showed that tri‐lineage engraftment in the peripheral blood was not affected by M‐CSF treatment at 4 and 12 weeks following allogeneic HCT (Fig EV5E). Merely few residual recipient BALB/c CD45.2^+^ cells were found (“alloHSPCs” in Fig EV5B), reflecting full bone marrow (BM) engraftment of CD45.1^+^ donor cells, which we confirmed at 12 weeks (Fig EV5F). These CD45.1^+^ donor cells were unaffected by M‐CSF treatment concerning long‐term engrafting HSPCs (KSL Flt3^−^CD150^+^CD48^−^) and GMPs alike in the BM 12 weeks after allogeneic HCT (Fig EV5G).

Together, our data reveal no contraindication for the short‐term treatment with M‐CSF following allogeneic HCT, suggesting that it should be a safe and feasible cytokine to promote antiviral activity in standard protocols of allogeneic HCT.

## Discussion

In this study we have identified the previously unknown protective effects of M‐CSF‐induced myelopoiesis against viral infection during the vulnerable leukopenic phase after HCT. We identified a coordinated differentiation program between myeloid and NK cells that plays a major role in reconstituting protection against viral infection and assigns a critical role to M‐CSF‐induced myelopoiesis in participating in antiviral immunity.

Immunocompromised individuals are prone to opportunistic infections including CMV viremia, but also to infection‐induced morbidity and mortality as shown in mice (Arber *et al*, 2003). Here, we used a murine model of immunosuppression after HCT to investigate the protective antiviral effects of M‐CSF‐induced myelopoiesis preceding MCMV infection. HCT is an important major therapeutic strategy that involves a conditioning therapy by which the recipient's hematopoietic system is immunosuppressed to foster engraftment of donor HSPCs. Patients encounter severe immunodeficiency after HCT that leaves them highly vulnerable to opportunistic bacterial, fungal, and viral infection before the donor's hematopoietic system is sufficiently reconstituted. Although improvements have been made in prophylaxis and management, viral infection, and reactivation, such as CMV, still contribute significantly to morbidity and mortality after allogeneic HCT (Zaia, 1990; Ljungman *et al*, 2002; Boeckh & Ljungman, 2009). Unfortunately, available antiviral drugs are associated with numerous adverse effects (Ahmed, 2011; El Chaer *et al*, 2016). For example, ganciclovir severely compromises myelopoiesis, and thus further aggravates susceptibility to secondary infections (Goodrich *et al*, 1993; Boeckh *et al*, 1996; Salzberger *et al*, 1997) and enhances risk to secondary malignancy (de Kanter *et al*, 2021). Although progress has been made with the introduction of letermovir as a non‐toxic antiviral agent, it might select for virus variants and virus breakthrough infections as well as late reactivation once cessation of prophylaxis occurs (Hill *et al*, 2021). Furthermore, as an agent targeting viral terminase complex it is limited to be used against CMV and is not effective against other viruses. Adoptive transfer protocols of lymphoid progenitors also have been proposed as a therapeutic strategy in refractory or high‐risk cases (Kaeuferle *et al*, 2019). Cell therapy approaches, however, require complex logistics, which limits their availability and leads to high costs. Given the remaining clinical need for both acute and prophylactic antiviral treatments, the application of M‐CSF may represent an attractive, cost‐effective, and broadly applicable antiviral host‐directed approach.

Several properties of M‐CSF make it an ideal candidate for accelerating immunocompetence recovery in HCT recipients and present key advantages over other myeloid cytokines used in clinical practice. We showed before in murine models that M‐CSF directly engages HSPCs and thus intervenes at the earliest point of the differentiation hierarchy to initiate the production of innate immune cells (Sarrazin *et al*, 2009; Mossadegh‐Keller *et al*, 2013; Kandalla *et al*, 2016). M‐CSF prophylaxis could therefore shorten the time of immune system reconstitution to reduce the risk of infections. Other cytokines, in particular G‐CSF, are also used to stimulate immune functionality. However, in contrast to M‐CSF, G‐CSF can only act on already existing mature or late myeloid progenitor cells to activate their functional competence. Since these cells will only develop weeks after HCT, G‐CSF will be ineffective in the early phase after HCT. By acting at the earliest point of the hematopoietic differentiation hierarchy, M‐CSF can stimulate myelopoiesis swiftly after conditioning therapy. Consistent with this, we showed previously *in vivo* in mice that M‐CSF but not G‐CSF can stimulate the increased production of myeloid cells from HSPCs and protect from bacterial and fungal infections (Kandalla *et al*, 2016). Importantly, in previous murine studies M‐CSF‐induced myelopoiesis neither compromises stem cell numbers or activity (Sarrazin *et al*, 2009), nor comes at the expense of the generation of other blood cell lineages like platelets that are important for restoring blood clotting activity (Kandalla *et al*, 2016). Here, we report an additional advantage of M‐CSF treatment by promoting rapid reconstitution of antiviral activity *in vivo* and protection from viral infection through a multistep myeloid and NK cell differentiation program. A significant advantage of the early action of M‐CSF on HSPCs appears to be the stimulation of a combination of innate immune cells that are required to combat pathogens. Whereas G‐CSF only stimulates granulocytes and their direct progenitors, M‐CSF stimulates the production of (i) granulocytes, mediating cytotoxic bacterial killing, (ii) monocytes and macrophages, capable of pathogen control by phagocytosis and reactive oxygen production, and (iii) dendritic cells with the strongest antigen presentation activity that alerts the adaptive immune system. In this study, we now show that M‐CSF also induced I‐IFN‐producing pDCs and indirectly stimulated NK cell differentiation and activation through induction of IL‐15‐producing monocytic cells, which together mediated strong antiviral activity.

Allogeneic HCT harbors the risk of acute and chronic GvHD (Ferrara *et al*, 2009). To date, most pre‐clinical studies indicated a beneficial effect of M‐CSF in allogeneic HCT. M‐CSF treatment applied in mice just prior to transplantation induced host macrophages to engulf alloreactive T cells (Hashimoto *et al*, 2011). Conversely, depletion of resident macrophages by abrogating the M‐CSF/CSF‐1R‐axis during the peri‐transplantation period aggravated GvHD *in vivo* (Macdonald *et al*, 2010). With respect to chronic GvHD in the human setting, a retrospective analysis of over 50 Japanese bone marrow transplant recipients receiving M‐CSF at or early after transplantation showed a lower risk to develop this complication (Kimura *et al*, 2012). This underpins the relevance of the timing of M‐CSF administration for the differential effect on GvHD development. Although the early application of M‐CSF peri‐transplantation mediated the antimicrobial effects described by us and lead to a lower rate of chronic GvHD, it must be noted that the therapeutic window appears to be smaller when the compound is applied later after engraftment of T cell‐replete allografts in mice (Alexander *et al*, 2014).

The M‐CSF prophylaxis described by us targets NK cells and pDCs, whose protective functions during CMV infection are well described both in mice (Alexandre *et al*, 2014; Brinkmann *et al*, 2015) as well as in humans (Brinkmann *et al*, 2015). This is important for a fast antiviral response under immunosuppressed and leukopenic conditions since an antiviral T cell response cannot be mounted, especially in the context of T cell‐depleted allografts. Under these circumstances, the development of engrafted T cells arising from donor HSPCs occurs much later than viral reactivation during immunosuppressive leukopenia. In line with this, a recent randomized trial demonstrated that allogeneic transplantation of *ex vivo* T cell‐depleted hematopoietic grafts clearly reduced the incidence of GvHD but was associated with a significant increase of non‐relapse mortality (Luznik *et al*, 2022). As the majority (> 50%) of these deaths were related to infectious complications, the use of M‐CSF peri‐transplantation may be a strategy to improve the overall outcome of T cell‐depleted allogeneic HCT.

The effect on NK cells described in this study is mediated by M‐CSF‐induced myelopoiesis, in particular by monocytes. The role of monocytes and macrophages in CMV infection is multifaceted. On the one hand, rodent studies have shown they can be target cells for MCMV infection (Hanson *et al*, 1999; Hokeness *et al*, 2005; Daley‐Bauer *et al*, 2012), thus serving as vehicles of CMV dissemination (Smith *et al*, 2004; Daley‐Bauer *et al*, 2014). On the other hand, the observation that macrophage depletion *in vivo* increased MCMV burden (Hanson *et al*, 1999), also supports a protective role during CMV infection. This ambiguity might be dependent on the context of infection or on the specific monocyte subpopulation. Whereas Ly6C^−^CX3CR1^hi^ patrolling monocytes are involved in CMV dissemination in the mouse (Daley‐Bauer *et al*, 2014), Ly6C^+^CCR2^+^ inflammatory monocytes can engage antiviral responses in early infection via direct or indirect mechanisms (Salazar‐Mather *et al*, 2002; Hokeness *et al*, 2005; Soudja *et al*, 2012; Rovis *et al*, 2016; Gawish *et al*, 2019). In murine models, Ly6C^+^CCR2^+^ inflammatory monocytes could initiate differentiation of memory CD8^+^ T and NK cells into antimicrobial effector cells (Soudja *et al*, 2012) or showed direct iNOS‐mediated antiviral effects (Rovis *et al*, 2016). I‐IFN signaling is also important for recruitment of CCR2^+^ inflammatory monocytes via MCP‐1/CCL2 *in vivo* (Salazar‐Mather *et al*, 2002). Thus, mice deficient for MCP‐1 or CCR2 showed a reduced accumulation of monocyte‐derived macrophages and NK cells in liver, increased viral titers, widespread virus‐induced liver pathology and reduced survival (Hokeness *et al*, 2005; Crane *et al*, 2009). Previously, murine studies showed that CD11c^hi^ DC‐derived IL‐15 promoted NK cell priming (Lucas *et al*, 2007) and that inflammatory monocyte‐derived IL‐15 could stimulate NK cell differentiation (Soudja *et al*, 2012). In the immunosuppressed settings investigated here, Ly6C^hi^ monocytes appeared to be more important than DCs for IL‐15 presentation, since they expressed higher levels of IL15Rα required for IL‐15 cross‐presentation to NK cells (Lucas *et al*, 2007). Consistent with the synergistic role of IL‐15 and I‐IFNs for NK cell activation demonstrated in murine studies (Nguyen *et al*, 2002; Lucas *et al*, 2007), we observed that both *IL15RA*‐ and *IFNAR1*‐deficiency in GMP‐derived myeloid cells abolished their protective effect against MCMV, whereas ectopic IL‐15 could rescue *IFNAR1*‐deficiency. This suggested that IL‐15 induction in monocytes required I‐IFNs that were mainly produced by M‐CSF‐induced pDCs. Together, our experiments revealed the surprising capacity of M‐CSF to initiate a fully synchronized differentiation program and cytokine mediated crosstalk between different myeloid and NK cell lineages to provide effective antiviral prophylaxis during leukopenia following HCT‐mediated immunosuppression. Compatible with this notion, M‐CSF therapy could not only be beneficial for HCT protocols but also for other settings of severe leukopenia, e.g., after chemotherapy or in septicemia. Furthermore, the myeloid‐mediated antiviral activity described here could also be more generally helpful to combat other viral infections. M‐CSF‐induced IL‐15 might promote innate immune responses via the induction of NK cells without a coinciding T helper type 2 cell‐dependent cytokine storm, as has been suggested for SARS‐CoV2 in humans (Kandikattu *et al*, 2020). This renders M‐CSF application an attractive, potentially antiviral treatment prophylaxis.

However, further studies are needed to evaluate the clinical employability of M‐CSF following HCT as a prophylaxis of CMV infection in humans. For this, phase I/II clinical trials will be needed to evaluate the addition of M‐CSF to the currently licensed cytokine treatment options comprising of G‐CSF and GM‐CSF.

## Materials and Methods

### Mice and *in vivo* treatments

For reconstitution 3,000 c‐Kit/CD117^+^Sca1^+^Lin^−^ HSPCs, isolated using a lineage depletion kit (Miltenyi Biotec) and FACS sorting from 6 to 8‐week CD45.1^+^ bone marrow, were injected with 150,000 cKit^−^Ter119^+^ CD45.2^+^ carrier cells (Miltenyi Biotec) and murine (baculovirus expressed) or human recombinant M‐CSF (Chiron/Novartis) in 200 μl PBS retroorbitally into lethally irradiated (160 kV, 25 mA, 6.9 Gy) 8–14 weeks sex‐matched CD45.2^+^ mice as described previously (Sarrazin *et al*, 2009; Mossadegh‐Keller *et al*, 2013). Myeloid or NK cells were depleted by multiple intraperitoneal injections of 100 μg of rat anti‐CD115 (Tagliani *et al*, 2011), anti‐NK1.1 mAb (Cocita *et al*, 2015) or control IgG in PBS before mice were infected with MCMV at day 14 post HSPC transplantation, a time point that was carefully chosen to extrapolate to typical CMV reactivation in humans which is commonly observed early, i.e., between days 40 and 100, after HCT (Teira *et al*, 2016). In total, 50,000 granulocyte‐monocyte progenitors (GMPs) (Lin^−^CD117^+^Sca^−^1^−^CD34^+^CD16/32^+^) from WT or *IL15R*α‐KO or *IFNAR1*‐KO mice were FACS sorted and injected on day 10 after HCT. For consistency, all survival analyses using Kaplan–Meier estimates pertaining to MCMV‐infected mice are illustrated until 35 days post HSPC transplantation, although several mice were observed until day 50 without any further signs of morbidity and mortality.

For allogeneic HCT, BALB/c CD45.2^+^ recipient and C57BL/6j CD45.1^+^ donor mice were used. In brief, BALB/c CD45.2^+^ recipient mice were irradiated with 5 Gy, followed by allogeneic HCT after 24 h. Imminently before (1 h) or shortly after (5 and 20 h) allogeneic HCT with 2 × 10^5^ Lin^−^ HSPCs from C57BL76j CD45.1^+^ donors, the mice received PBS or 10 μg of baculoviral expressed human M‐CSF. Following alloHCT, scoring for disease activity was performed according to Lai *et al* (2012) on days 5, 10, 13, 15, 20, and 30, as well as donor HSPC‐derived blood cells were ascertained on day 30 in accordance with Alexander *et al* (2014).

For all *in vivo* experiments, block randomization was used to establish identical or similar group sizes.

### MCMV infection, viral loads, and histopathology

Two weeks after HCT, mice were injected intraperitoneally with 5,000 plaque‐forming units (PFU) of MCMV K181 v70 in 200 μl PBS. Viral loads were measured by quantitative reverse transcription polymerase chain reaction (RT‐qPCR) of *Ie1* mRNA (Baranek *et al*, 2012) extracted from frozen tissues 36–40 h (1.5 days) or 72 h as reported previously (Cocita *et al*, 2015). Paraformaldehyde‐fixed (4%), paraffin‐embedded and hematoxylin and eosin (H&E)‐stained liver sections were scored by a trained veterinary pathologist blinded to sample identity for indicated parameters.

### Human hematopoietic stem and progenitor cell differentiation

Human G‐CSF‐mobilized HSPCs were obtained from leukapheresis samples from the Department of Transfusion Medicine of the TU Dresden. On the day of donation, a Ficoll‐density gradient centrifugation step was performed as described previously (Subburayalu *et al*, 2021) to isolate the peripheral blood mononuclear cell (PBMC) layer containing mainly mononuclear cells, T cells, NK cells, HSPCs and low‐density granulocytes. To evaluate the effect of M‐CSF on selective co‐cultures between mononuclear cells, NK cells and HSPCs, T cells and low‐density granulocytes were depleted with anti‐CD3 and anti‐CD15 microbeads using a QuadroMACS separator (Miltenyi Biotec, Cat. 130‐090‐976) and LS columns (Miltenyi Biotec, Cat. 130‐042‐401). Cell viability and purity of selection were confirmed by light microscopic assessment of modified Giemsa stained cytospins as detailed previously (Bashant *et al*, 2019). 2 × 10^5^ cells (1 × 10^6^ cells ml^−1^) of the CD3/CD15‐depleted G‐CSF‐mobilized PBMCs were subsequently transferred to 96U‐bottom ultralow adherence plates (Nunclon Sphera, Cat. 174925) to be cultured in StemPro34 serum‐free medium (Gibco, Cat. 10639011) with 1× penicillin/streptomycin (Thermo Fisher, Cat. 15140122) supplemented with stem cell factor (SCF) (R&D, Cat. 255‐SC‐050/CF, 20 ng ml^−1^) ± the following cytokine compositions: (i) none, (ii) recombinant human IL‐3 (R&D, Cat. 203‐IL‐050/CF, 25 ng ml^−1^) or (iii) human M‐CSF recombinant protein (Invitrogen, Cat. PHC9501, 100 ng ml^−1^). A partial medium change was performed every 48 h with 2× cytokine composites to replenish cytokines. Cell differentiation and viability were confirmed using cytospins on days 5 and 9.

### Flow cytometry analysis

Spleen leukocyte suspensions were prepared using DNAse I and collagenase D (Baranek *et al*, 2012). For FACS sorting and analysis, we used previously reported protocols (Mossadegh‐Keller *et al*, 2013; Kandalla *et al*, 2016), published HSPC definitions (Bryder *et al*, 2006), indicated antibodies (see Table EV1), FACSCanto, LSRI, LSRII and FACSAriaIII equipment and DIVA software (BD), analyzing only populations with at least 200 events.

For human samples, an antibody panel was used to distinguish progenitors of human HSPCs (Lin^−^CD34^+^) such as common lymphoid progenitors (CLPs, Lin^−^CD34^+^CD38^−/low^CD45RA^+^), common myeloid progenitors (CMPs, Lin^−^CD34^+^CD38^+^CD45RA^−^) or granulocyte‐macrophage progenitors (GMPs, Lin^−^CD34^+^CD38^+^CD45RA^+^) with mature GMPs additionally expressing HLA‐DR from mature myeloid cells (either CD11b^+^CD66b^−^ or Lin^+^ ± CD14/CD16) whose IL15Rα expression was quantified. For NK cell abundance and activity, an optimized panel as published previously was used (Costanzo *et al*, 2015). For flow cytometry, 2 × 10^5^ cells were harvested on the day of seeding (day 0) or on days 5 and 9, respectively.

### Immunofluorescence

Freshly frozen OCT embedded (Sakura Finetek). 8 μm sections (Leica CM3050 S cryostat) were fixed 10′ in 4°C acetone, blocked 30′ with PBS/2%BSA, stained with 1:100 directly coupled antibody (see Table EV2) in PBS/2%BSA for 1 h, mounted in ProlongGold (Invitrogen) and acquired on a LSM780 Carl Zeiss microscope.

### Microfluidic real‐time RT‐PCR gene expression analysis

Total mRNA extraction from 50,000 FACS‐sorted cells and cDNA synthesis were performed with μMACS one step T7 template kit (Miltenyi) and specific gene expression (primers in Table EV3) was detected according to Fluidigm protocols as previously described (Soucie *et al*, 2016) or by SybrGreen method (Mossadegh‐Keller *et al*, 2013). Ct values were calculated by BioMark Real‐time PCR Analysis software (Fluidigm) using the ΔΔCt method and *HPRT* for normalization.

### Statistical analysis

None of the mice or human samples were excluded from the experiments. Multiple statistical methods, including Student's *t*‐test, Mann–Whitney *U*‐test, log‐rank (Mantel‐Cox) test were used in this study depending on the data type, and the details can be found in the figure legends: test used and exact value of *n*. For *in vivo* experiments, block randomization was used to establish identical or similar group sizes. No statistical methods were used to predetermine sample sizes. Veterinary pathologists assessing and scoring liver sections were blinded to sample identity for indicated parameters. No further blinding was performed. Experiments were performed independently at least twice.

Data between two groups were analyzed with unpaired Student's *t*‐tests. All statistical analyses were performed using GraphPad Prism (Version 9.4.0, GraphPad Software, La Jolla, CA, USA). All data were analyzed using the FlowJo software (Version 10.8.1; Tree Star, Ashland, OR, USA) and expressed as medians + individual data points or means ± SEM unless stated otherwise. *P*‐values less than 0.05 were considered significant.

### Study approval

Procedures involving animals and their care were conducted in accordance with institutional guidelines and internal laws and policies and approved by the Institutional Animal Care and Use Committee (APAFIS #17258‐2018102318448168‐v5 and APAFIS #36188‐2022032912082580‐v6). All mouse experiments were performed under specific pathogen‐free conditions and animals were monitored daily for signs of morbidity.

The use of human samples was approved by the Ethical Review Committee of the TU Dresden (approval no. EK477112016 and EK393092016) and all human research conformed to the Declaration of Helsinki and the Department of Health and Human Services Belmont Report. Informed consent was obtained from all participants.

## Author contributions

**Prashanth K Kandalla:** Conceptualization; data curation; formal analysis; validation; investigation; visualization; methodology; writing – original draft; project administration; writing – review and editing. **Julien Subburayalu:** Conceptualization; data curation; formal analysis; validation; investigation; visualization; methodology; writing – original draft; project administration; writing – review and editing. **Clément Cocita:** Resources; formal analysis; investigation; methodology. **Bérengère de Laval:** Resources; data curation; formal analysis; validation; investigation; visualization; methodology; writing – review and editing. **Elena Tomasello:** Resources; methodology. **Johanna Iacono:** Methodology. **Jessica Nitsche:** Investigation. **Maria M Canali:** Resources. **Wilfried Cathou:** Formal analysis; methodology. **Gilles Bessou:** Resources. **Noushin Mossadegh‐Keller:** Methodology. **Caroline Huber:** Methodology. **Guy Mouchiroud:** Resources. **Roland P Bourette:** Resources. **Marie‐France Grasset:** Resources. **Martin Bornhäuser:** Writing – review and editing. **Sandrine Sarrazin:** Formal analysis; methodology; project administration; writing – review and editing. **Marc Dalod:** Conceptualization; resources; formal analysis; methodology; writing – review and editing. **Michael H Sieweke:** Conceptualization; formal analysis; supervision; funding acquisition; validation; writing – original draft; project administration; writing – review and editing.

## Disclosure and competing interests statement

MHS is a patent holder of WO2014167018A1 (Use of M‐CSF for preventing or treating myeloid cytopenia and related complications). The authors declare no further competing interests.

## For more information

- Authors' home website: https://tu‐dresden.de/cmcb/crtd/forschungsgruppen/crtd‐forschungsgruppen/sieweke & www.ciml.univ‐mrs.fr/science/lab‐michael‐sieweke/stem‐cell‐and‐macrophage‐biology.
- Information on CMV in transplanted patients provided by Cedars Sinai: https://www.cedars‐sinai.org/health‐library/diseases‐and‐conditions/c/cmv‐and‐transplant‐patients.html.

## Supporting information



Expanded View Figures PDF

Table EV1

Table EV2

Table EV3

Source Data for Expanded View

PDF+

Source Data for Figure 1

Source Data for Figure 2

Source Data for Figure 3

Source Data for Figure 4

Source Data for Figure 5

Source Data for Figure 6

Source Data for Figure 7

Source Data for Figure 8

## Data Availability

This study includes no data deposited in external repositories.
