# Supplementary material for: M‐CSF directs myeloid and NK cell differentiation to protect from CMV after hematopoietic cell transplantation
Source: EMBO Mol Med. 2023 Aug 28;15(11):e17694. doi: 10.15252/emmm.202317694 (PMC10630876; doi:10.15252/emmm.202317694)
Supplement: Supplementary file 1 — Expanded View Figures PDF [file EMMM-15-e17694-s005.pdf]

Expanded View Figures

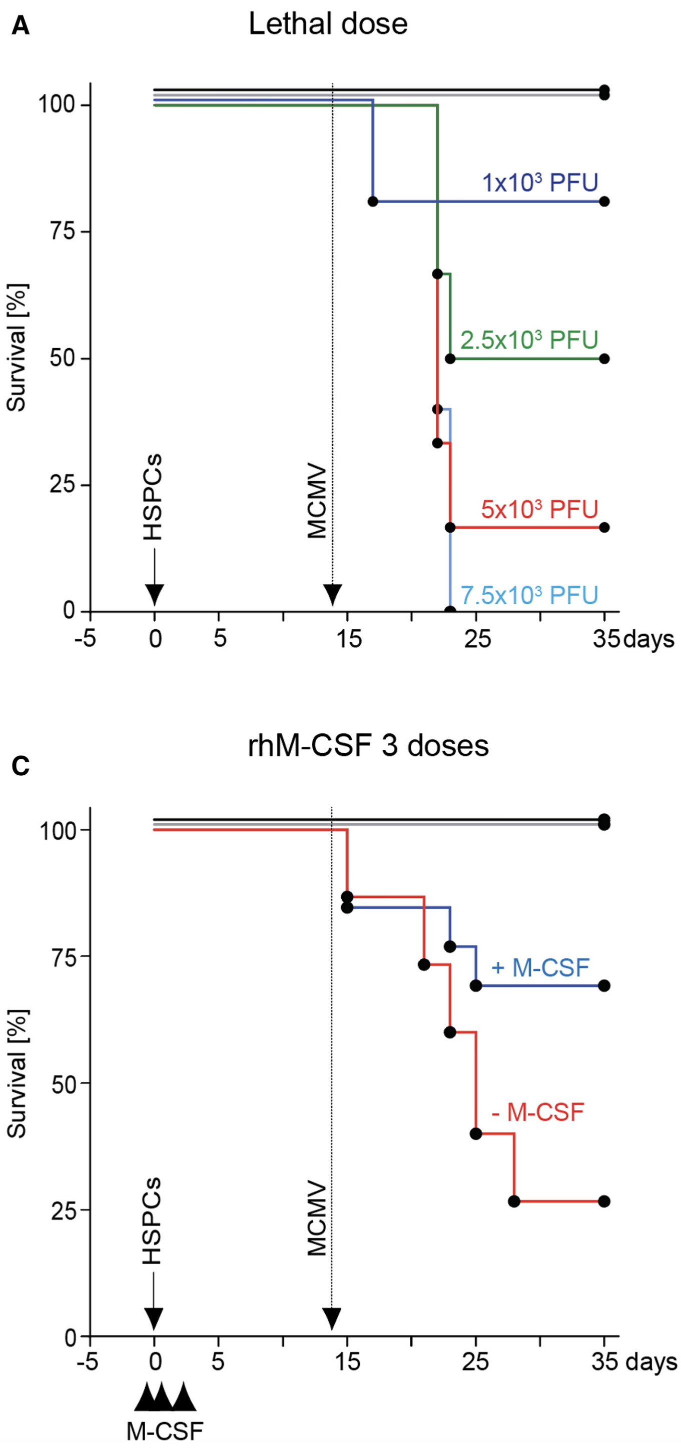

Figure EV1.

© 2023 The Authors EMBO Molecular Medicine 15: e17694 | 2023 21 of 20

Figure EV1. Titration of MCMV infection and M-CSF treatments.

A Survival of HSPC-transplanted mice after MCMV infection. Two weeks after HCT, mice received MCMV intraperitoneally: 1,000 PFU (violet; $n = 5$), 2,500 PFU (green; $n = 6$), 5,000 PFU (red; $n = 6$) and 7,500 PFU (blue; $n = 5$). Transplantation controls (black; $n = 10$). Non-irradiated, non-transplanted mice with 7,500 PFU served as infection controls (brown; $n = 6$).

B Treatment with different doses and sources of M-CSF. Survival of mice after infection (arrow), control (−M-CSF, red) or M-CSF (+M-CSF, blue) or transplanted, uninfected controls (black). HSPC-transplantation (solid arrow), MCMV infection (stippled) and different intravenous doses of control or M-CSF. Treatment with 4 doses (−1 h, d+1, d+3, d+5) of 10 μg baculoviral-expressed mouse M-CSF (−M-CSF, $n = 12$; +M-CSF, $n = 12$; control, $n = 5$).

C Like B with 3 doses (−1 h, +5 h, +18 h) of 10 μg human recombinant M-CSF (−M-CSF, $n = 15$; +M-CSF, $n = 13$; control, $n = 5$).

D Like B with a single dose (+5 h) of 10 μg baculoviral-expressed mouse M-CSF (−M-CSF, $n = 8$; +M-CSF, $n = 9$; control, $n = 2$).

Data information: $P < 0.0001$ comparing +M-CSF versus −M-CSF as determined by Mantel-Cox test (B–D).
Source data are available online for this figure.

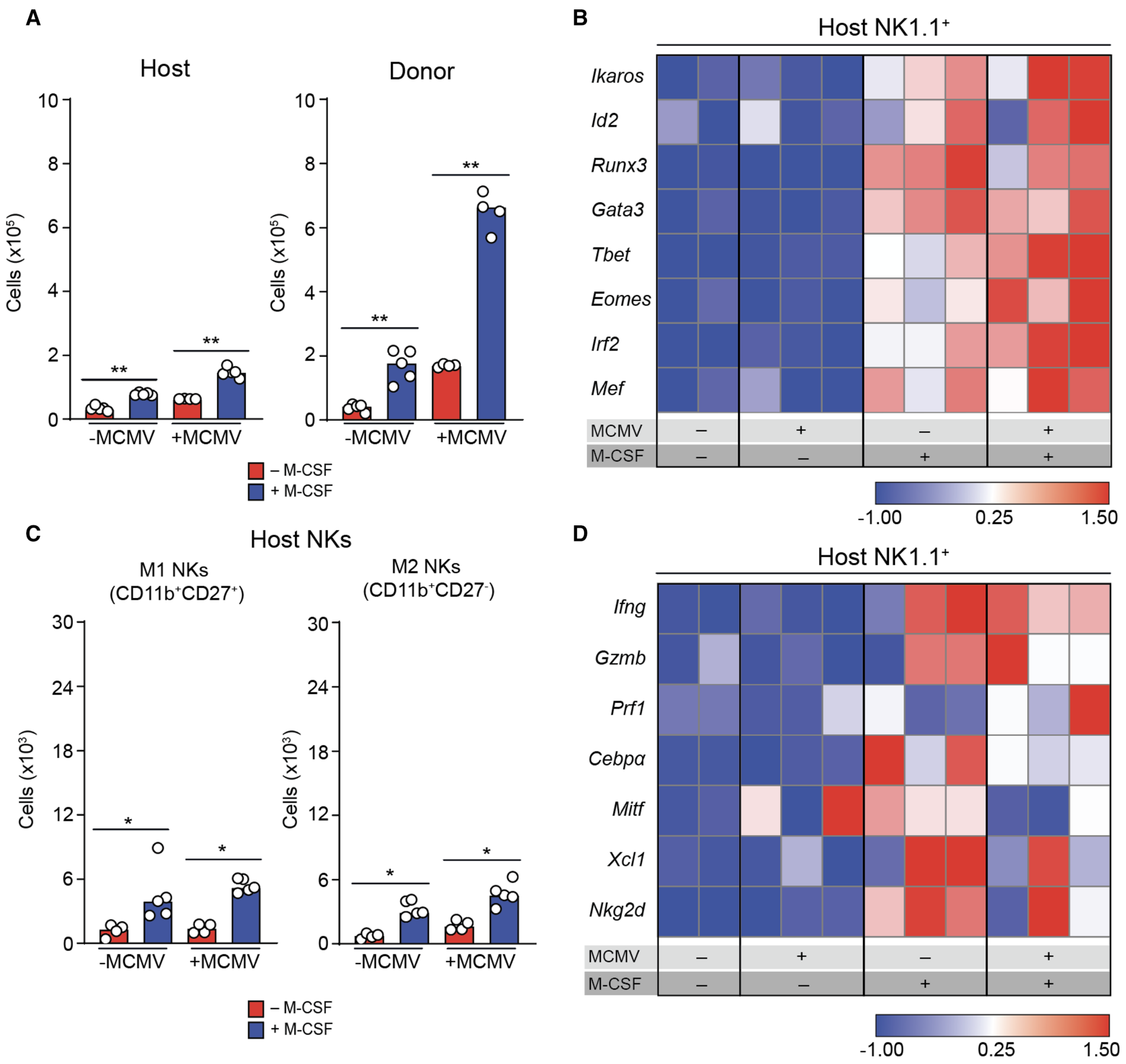

Figure EV2.

© 2023 The Authors

◀ **Figure EV2. M-CSF effect on NK cell production, maturation and differentiation in donor and recipient cells after hematopoietic cell transplantation.**

A Median of absolute numbers of ($CD45.2^+$) recipient (left) and ($CD45.1^+$) donor (right) NK cells ($CD19^-CD3^-Ly6G^-NK1.1^+$). $^{**}P < 0.01$ by Mann–Whitney *U*-test.

B Gene expression profiling of transcription factors measured by nanofluidic Fluidigm array RT-qPCR of host-derived NK cells, which were isolated from the spleens of control or M-CSF-treated recipient mice 1.5 days after MCMV of infection or time-matched, mock-infected, HSPC-transplanted mice.

C Median of absolute numbers of host-derived M1 NK cells ($CD11b^+CD27^+$) and host-derived M2 NK cells ($CD11b^+CD27^-$) in the spleen of PBS control or M-CSF-treated recipient mice 1.5 days after MCMV or mock infection 14 days after HSPC transplantation. $^{*}P < 0.05$ by Mann–Whitney *U*-test.

D Gene expression profiling of host-derived NK cells, which were isolated from the spleen of control or M-CSF-treated recipient mice 1.5 days after MCMV or mock infection for activation and maturation related factors by RT-qPCR.

Source data are available online for this figure.

© 2023 The Authors

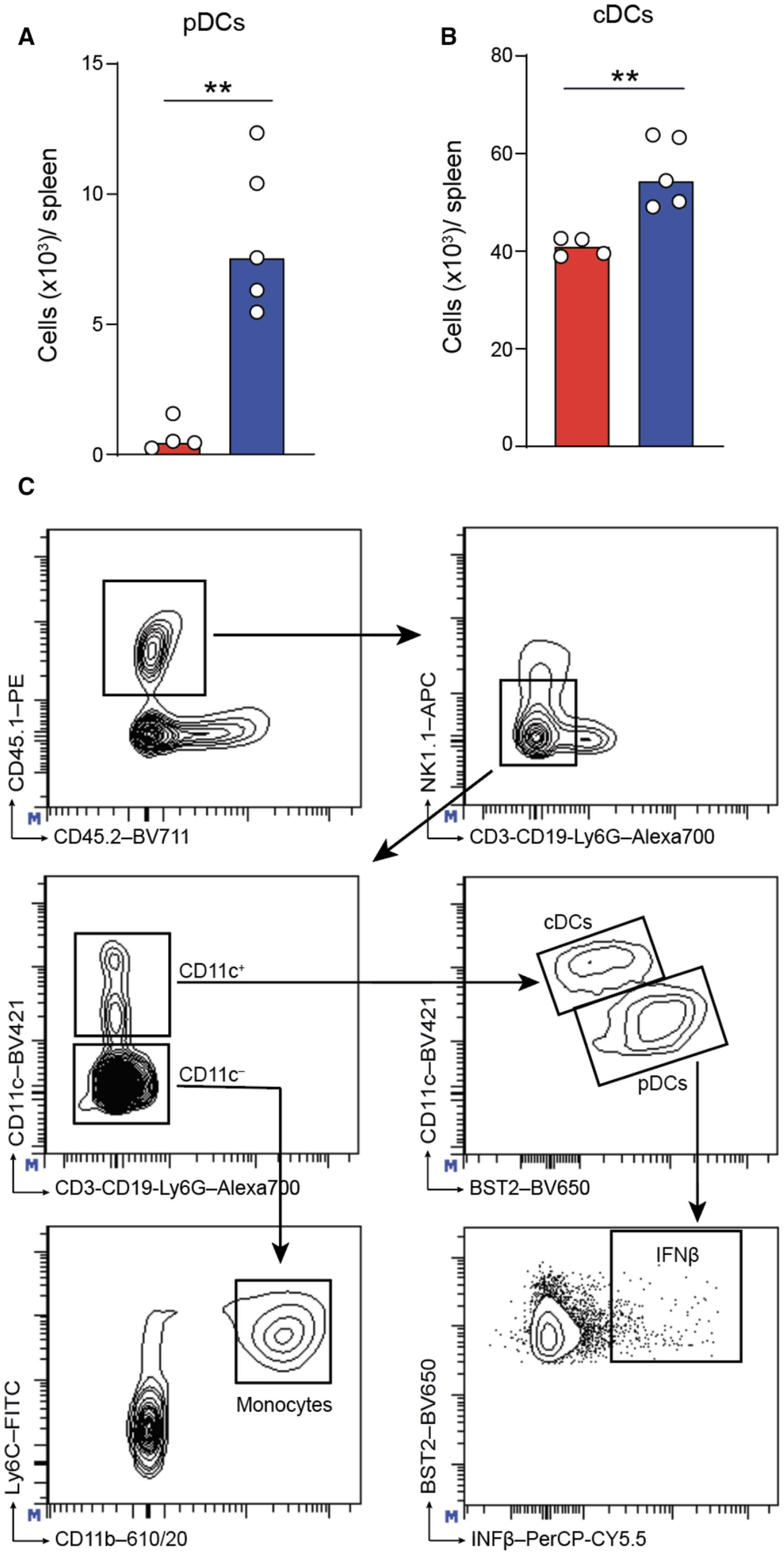

Figure EV3. M-CSF increases myelopoiesis of plasmacytoid dendritic cells and conventional dendritic cells.

A Median of absolute numbers of donor-derived spleen pDCs ($Lin^{-}CD11c^{lo}BST2^{high}$) of mice treated with PBS control or M-CSF 14 days after HCT and analyzed after an additional 1.5 days of MCMV or mock infection. **$P < 0.01$ by Mann–Whitney *U*-test.

B Median of absolute numbers of cDCs ($Lin^{-}CD11c^{+}BST2^{-/low}$) of mice treated with PBS control or M-CSF 14 days after HCT and analyzed after an additional 1.5 days of MCMV or mock infection. **$P < 0.01$ by Mann–Whitney *U*-test.

C Gating strategy for $CD45.1^{+}$ monocytes, pDCs, IFN-β and cDCs.

Source data are available online for this figure.

© 2023 The Authors

Figure EV4. Gating strategy for M-CSF-driven myelopoiesis in G-CSF-mobilized human PBMCs, IL15Rα expression and NK cell frequency and activity.

A Workflow for G-CSF-mobilized leukapheresis samples
B Gating strategy for flow cytometric assessment.
C "Live" singlets assessed for CD11b ("Myeloid" cells): polymorphonuclear neutrophils (PMNs, $CD11b^+CD66b^+$) and $CD11b^+CD66b^−$ "Monocytic" cells. The "Monocytes" ($CD14^+$) gate permitted identification of $CD14^+CD16^+$ monocyte-derived macrophages. At baseline (d0), this gating strategy was used to identify classical monocytes (CMs, $CD14^+CD16^−$), intermediate monocytes (IMs, $CD14^+CD16^+$) or non-classical monocytes (NCMs, $CD14^−CD16^+$) accordingly.
D IL15Rα expression measured on myeloid, monocytic cells, or monocytes identified in (C).
E The gating strategy of OMIP-027 with minor modifications by gating on the $Lin^−$ cells from the dot plot shown in (C).

Source data are available online for this figure.

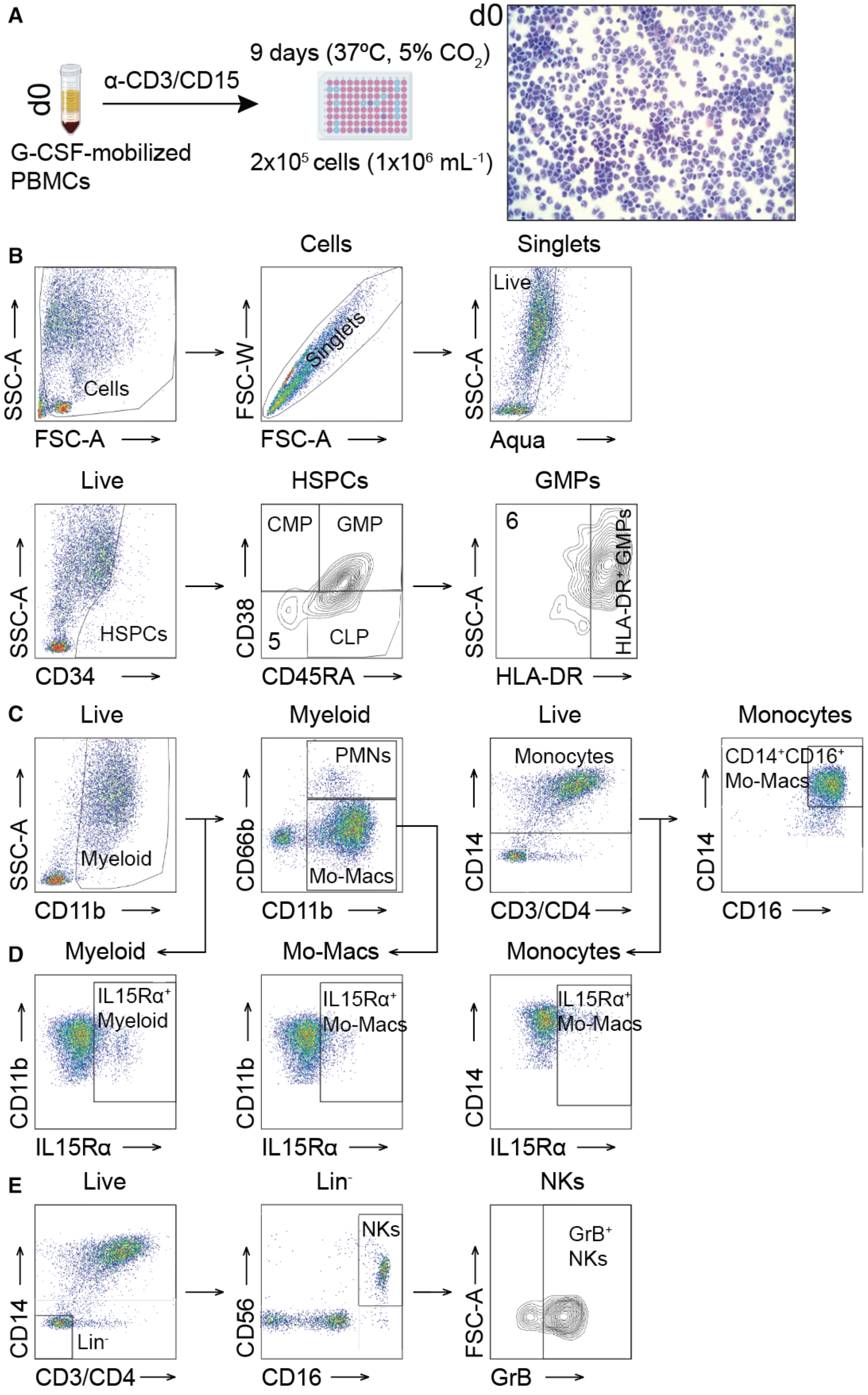

Figure EV4.

© 2023 The Authors

Figure EV5. M-CSF does neither confer adverse effects on tri-lineage long-term engraftment nor on GvHD after allogeneic hematopoietic cell transplantation.

A The protocol for allogeneic hematopoietic stem cell transplantations (alloHCT) between BALB/c $CD45.2^+$ recipient and C57BL/6j $CD45.1^+$ donor mice. Before (1 h) or after (5 h, 20 h) alloHCT with 2×10^5 lineage negative (Lin^-) hematopoietic stem and progenitor cells (HSPCs), mice received PBS or 10 μg baculoviral-expressed human M-CSF.
B Engraftment of $CD45.1^+$ cells was assessed at 4 and 12 weeks after alloHCT using the gating strategy by Alexander *et al* (2014).
C Quantification of inflammatory $Ly6C^{HI}$ $CD11b^+F4/80^+$ monocytes (monos) of $CD45.1^+$ cells.
D Disease scoring was applied as published by Lai *et al* (2012).
E Tri-lineage engraftment ($CD3\varepsilon^+$ T cells, $CD19^+$ B cells, $CD11b^+SSC\text{-}A^{LOW}$ monocytes) at 4 and 12 weeks post-HCT in the blood.
F $CD45.1^+$ cells in the bone marrow (BM) 12 weeks after alloHCT.
G Percentage of HSCs (KSL $Flt3^-CD150^+CD48^-$) and GMPs in $CD45.1^+$ lineage negative BM cells 12 weeks after alloHCT.

Data information: The data are illustrated as mean ± SEM. The Mann–Whitney *U*-test was used to test for statistical significance between PBS-treated ($n = 4$) or M-CSF-treated allografted mice ($n = 5$). *$P < 0.05$, ns = not significant.
Source data are available online for this figure.

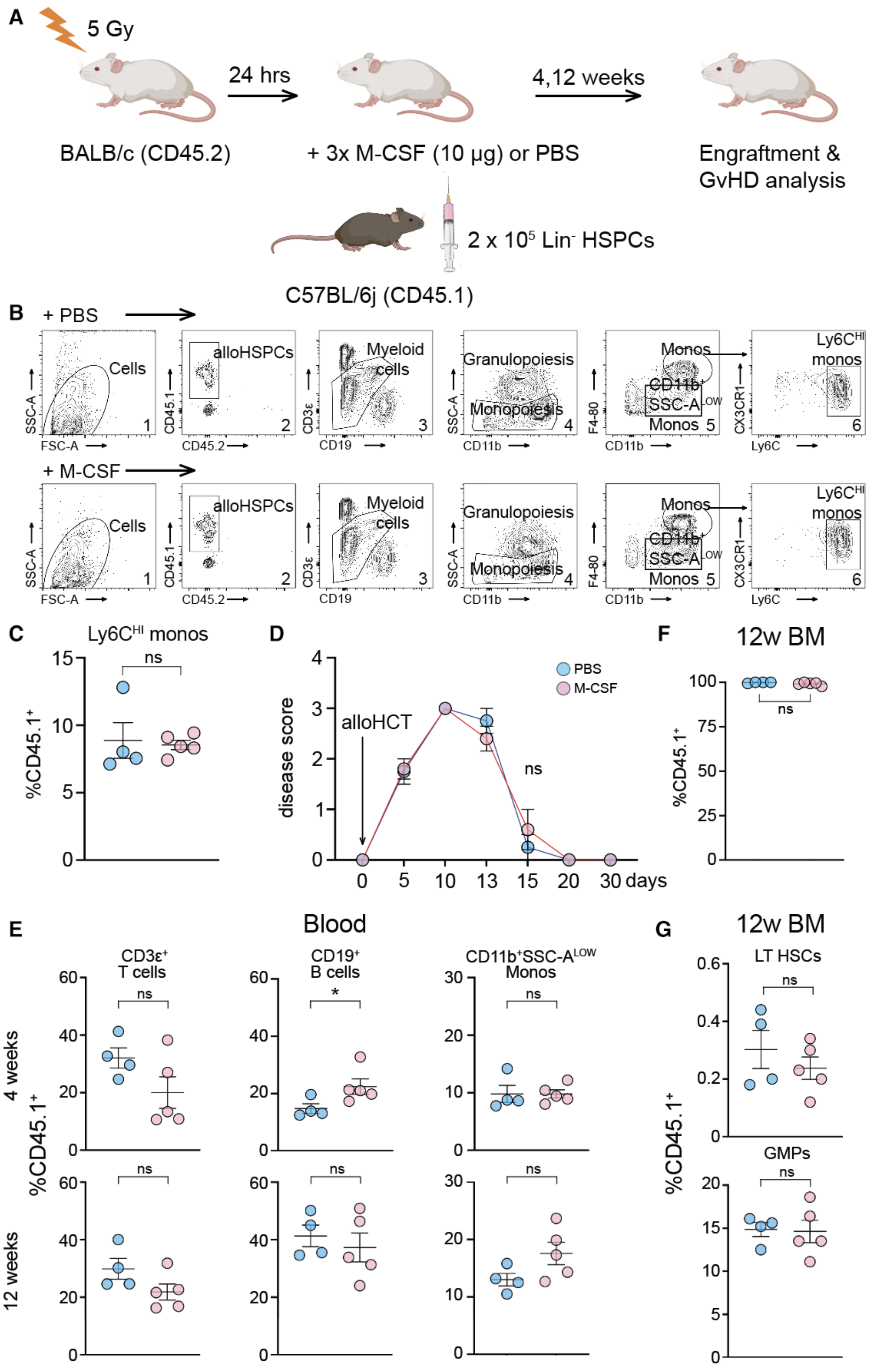

Figure EV5.

 © 2023 The Authors
