# Supplementary material for: M‐CSF directs myeloid and NK cell differentiation to protect from CMV after hematopoietic cell transplantation
Source: EMBO Mol Med. 2023 Aug 28;15(11):e17694. doi: 10.15252/emmm.202317694 (PMC10630876; doi:10.15252/emmm.202317694)
Supplement: Supplementary file 2 — Table EV1 [file EMMM-15-e17694-s007.docx]

| **Antigen** | **Fluorophore** | **Clone** | **Manufacturer** | **Cat. No.** |
| --- | --- | --- | --- | --- |
| CD45R/B220 | APC | RA3-6B2 | Biolegend | 103211 |
| CD11b | PerCP | M1/70 | Biolegend | 101229 |
| CD11c | A700 | HL3 | BD Pharmingen | 560583 |
| CD19 | APC | 6D5 | Biolegend | 115511 |
| CD19 | FITC | 1D3 | Biolegend | 152403 |
| CD19 | PE | 1D3 | Biolegend | 152407 |
| CD3ε | PE | 145-2c11 | Biolegend | 100307 |
| CD4 | PE | RM4-5 | Biolegend | 100511 |
| CD4 | PerCP | RM4-5 | Biolegend | 100537 |
| CD8α | Pacific Blue | 53-6.7 | Biolegend | 100728 |
| CD8α | PE | 53-6.7 | Biolegend | 100707 |
| CD8α | PerCP | 53-6.7 | Biolegend | 100731 |
| CD8β | PE | H35-17.2 | eBioscience | 12-0083-82 |
| CD49b | APC | DX5 | Biolegend | 108909 |
| Granzyme B | APC | GB11 | eBioscience | GRB05 |
| IFNβ | FITC | RMMB1 | Novus Biologicals | 22400-3 |
| IFNγ | A700 | XMG1.2 | eBioscience | 56-7311-82 |
| IL-12 | APC | C15-6 | Biolegend | 505205 |
| Ki67 | Pacific Blue | B56 | Biolegend | 350513 |
| NK-1.1 | BV510 | PK136 | BD Biosciences | 563096 |
| NK-1.1 | APC | PK136 | BD Biosciences | 561117 |
| NKp46 | PE | 29A1.4 | eBioscience | 12-3351-82 |
| Streptavidin | PE-Cy7 |  | BD Biosciences | 557598 |
| Purified NK1.1 |  | PK136 | BD Biosciences | 553162 |
| Purified polyclonal rat IgG, F(ab’)_2_ fragment specific |  |  | Jackson ImmunoResearch | 212-005-106 |
| CD117 | BV605 | 2B8 | Biolegend | 105847 |
| Sca-1 | PerCP-Cy5.5 | D7 | eBioscience | 45-5981-82 |
| CD34 | A700 | RAM34 | eBioscience | 56-0341-82 |
| CD16/32 | PE | 2.4G2 | BD Biosciences | 553145 |
| CD11b | PECF594 | M1/70 | BD Biosciences | 562287 |
| Ly6G | FITC | 1A8 | eBioscience | 11-9668-82 |
| Ly6C | APC | HK1.4 | eBioscience | 17-5932-82 |
| Ultra-LEAF Purified CD115 |  | AFS98 | Biolegend | 135537 |
| CD45.2 | PerCP/Cy5.5 | 104 | BD Biosciences | 552950 |
| CD45.1 | V450 | A20 | BD Biosciences | 560520 |
| Ter119 |  | TER-119 | eBioscience | MA1-70078 |
| CD71 |  | R17217 | eBioscience | 14-0711-82 |
| LIVE/DEAD Fixable Violet |  |  | Invitrogen | L34955 |
| LIVE/DEAD Fixable Aqua |  |  | Invitrogen | L34957 |
| CD3 | APC-Cy7 | SP34-2 | BD Biosciences | 557757 |
| CD4 | APC-H7 | SK3 | BD Biosciences | 641398 |
| CD14 | PE-Cy5 | TuK4 | Invitrogen | MHCD1406 |
| CD16 | Pacific Blue | 3G8 | BD Biosciences | 558122 |
| CD56 | PE-Cy7 | NCAM16.2 | BD Biosciences | 335809 |
| Granzyme B | PE CF594 | GB11 | BD Biosciences | 562462 |
| CD34 | PE-Cy7 | 581 | Biolegend | 343515 |
| CD38 | BV650 | HIT2 | Biolegend | 303505 |
| CD45RA | Pacific Blue | HI100 | Biolegend | 304117 |
| CD11b | FITC | M1/70 | Biolegend | 101205 |
| CD64 | PE/Dazzle594 | 10.1 | Biolegend | 305031 |
| CD66b | APC-Cy7 | G10F5 | Biolegend | 305125 |
| HLA-DR | AF647 | L243 | Biolegend | 307621 |
| IL15Rα | PE | JM7A4 | Biolegend | 330207 |
| CX3CR1 | FITC | SA011F11 | Biolegend | 149019 |
| CD45.2 | PerCP/Cy5.5 | 104 | BD Biosciences | 552950 |
| CD45.1 | V450 | A20 | BD Biosciences | 560520 |
| CD11b | BV605 | M1/70 | BD Biosciences | 563015 |
| F4/80 | BV785 | BM8 | Biolegend | 123141 |
| CD3ε | APC/AF6 | 145.2C11 | BD Biosciences |  |
| Ly6C | AC7 | HK1.4 | Biolegend | 128025 |
| CD19 | PEC7 | 6D5 | Biolegend | 115519 |

**Table EV1.** **Information on flow cytometry antibodies.** The following antibodies were used according to the manufacturer’s instructions throughout the study. Antibodies from LIVE/DEAD Fixable Aqua onwards refers to the antibodies used for the experiments using G-CSF-mobilized HSPCs. Antibodies from CX3CR1 onwards refers to the antibodies used for the allotransplantation studies.
