# Supplementary material for: M‐CSF directs myeloid and NK cell differentiation to protect from CMV after hematopoietic cell transplantation
Source: EMBO Mol Med. 2023 Aug 28;15(11):e17694. doi: 10.15252/emmm.202317694 (PMC10630876; doi:10.15252/emmm.202317694)
Supplement: Supplementary file 3 — Table EV2 [file EMMM-15-e17694-s014.docx]

| **Antigen** | **Fluorophore** | **Clone** | **Manufacturer** | **Cat. No.** |
| --- | --- | --- | --- | --- |
| NK-1.1 (IgG2a) | Unconjugated | PK136 | Invitrogen | MA1-70100 |
| m123/IE-1 (MCMV) | Unconjugated | IE1.01 | Capri (Center for Proteomics) | HR-MCMV-12 |
| Goat anti-Mouse IgG2a Cross-adsorbed secondary antibody | Alexa Fluor 594 |  | Invitrogen | A-21135 |
| **Table EV2.** **Information on immunofluorescence antibodies.** The following antibodies were used according to the manufacturer’s instructions throughout the study. | | | | |
