# Supplementary figures and images for: M‐CSF directs myeloid and NK cell differentiation to protect from CMV after hematopoietic cell transplantation
Source: EMBO Mol Med. 2023 Aug 28;15(11):e17694. doi: 10.15252/emmm.202317694 (PMC10630876; doi:10.15252/emmm.202317694)

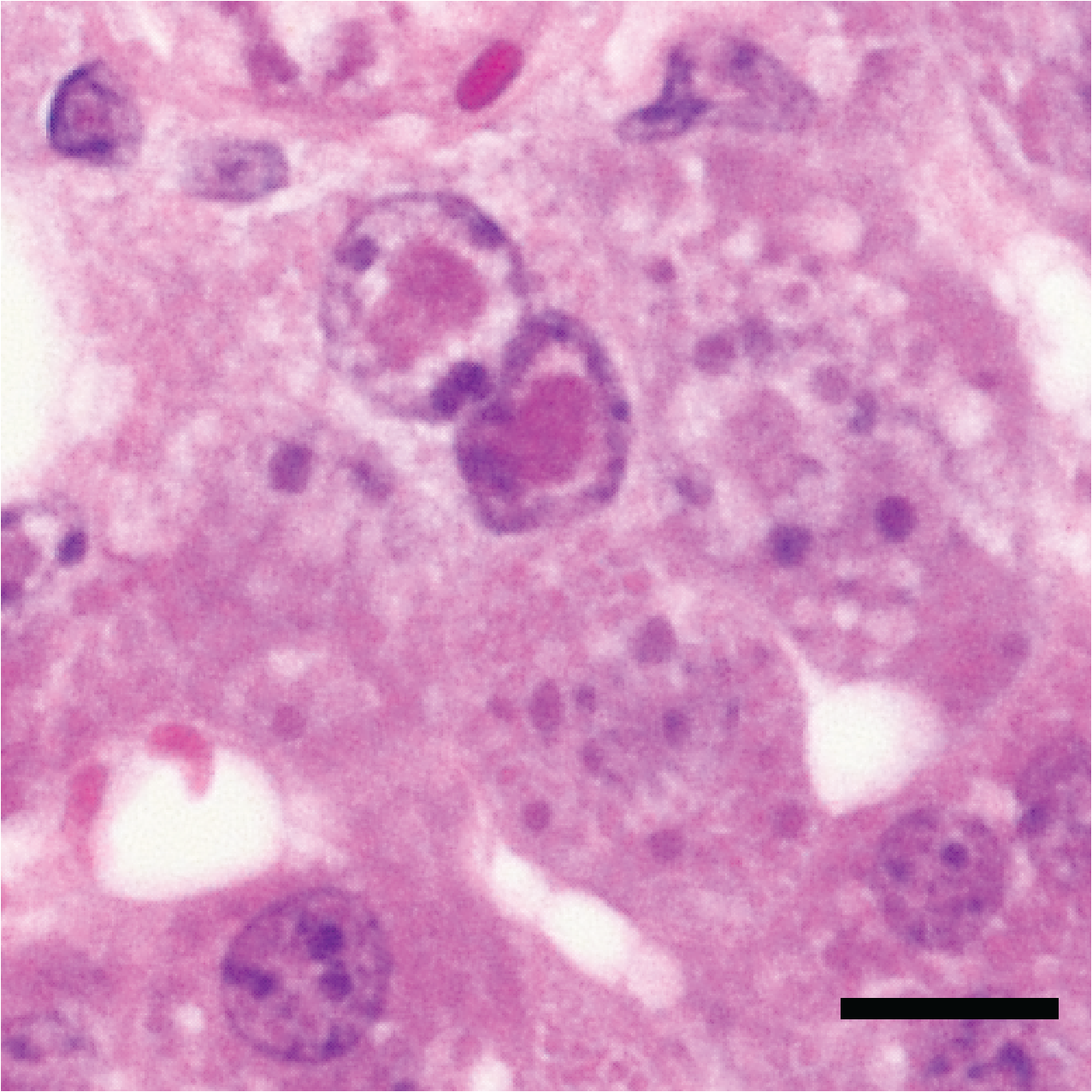

Supplement: Supplementary file 7 — Source Data for Figure 1 [file EMMM-15-e17694-s015.zip › Figure 1/1F/1F PBS d8.tiff]

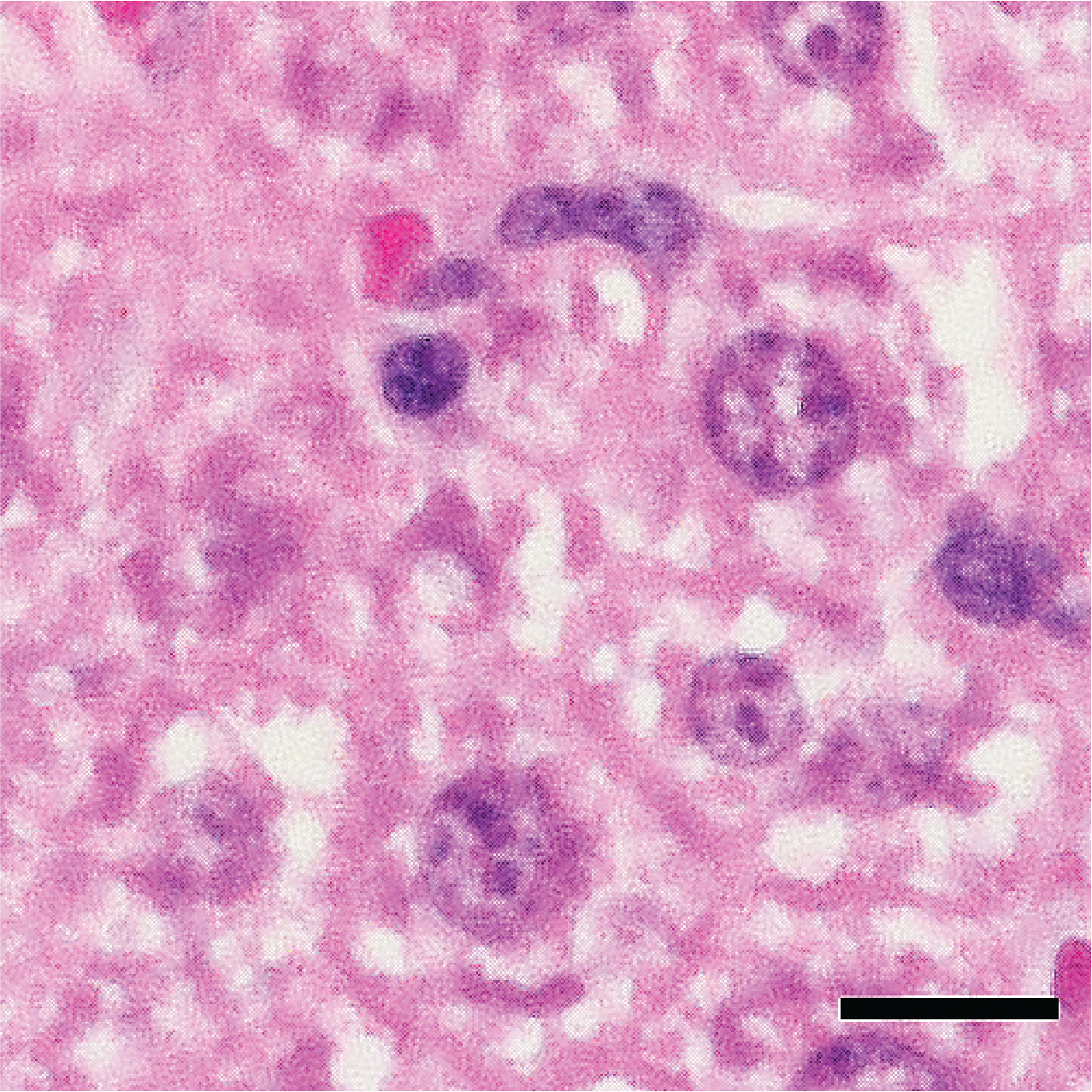

Supplement: Supplementary file 7 — Source Data for Figure 1 [file EMMM-15-e17694-s015.zip › Figure 1/1F/1F M-CSF d8.tiff]

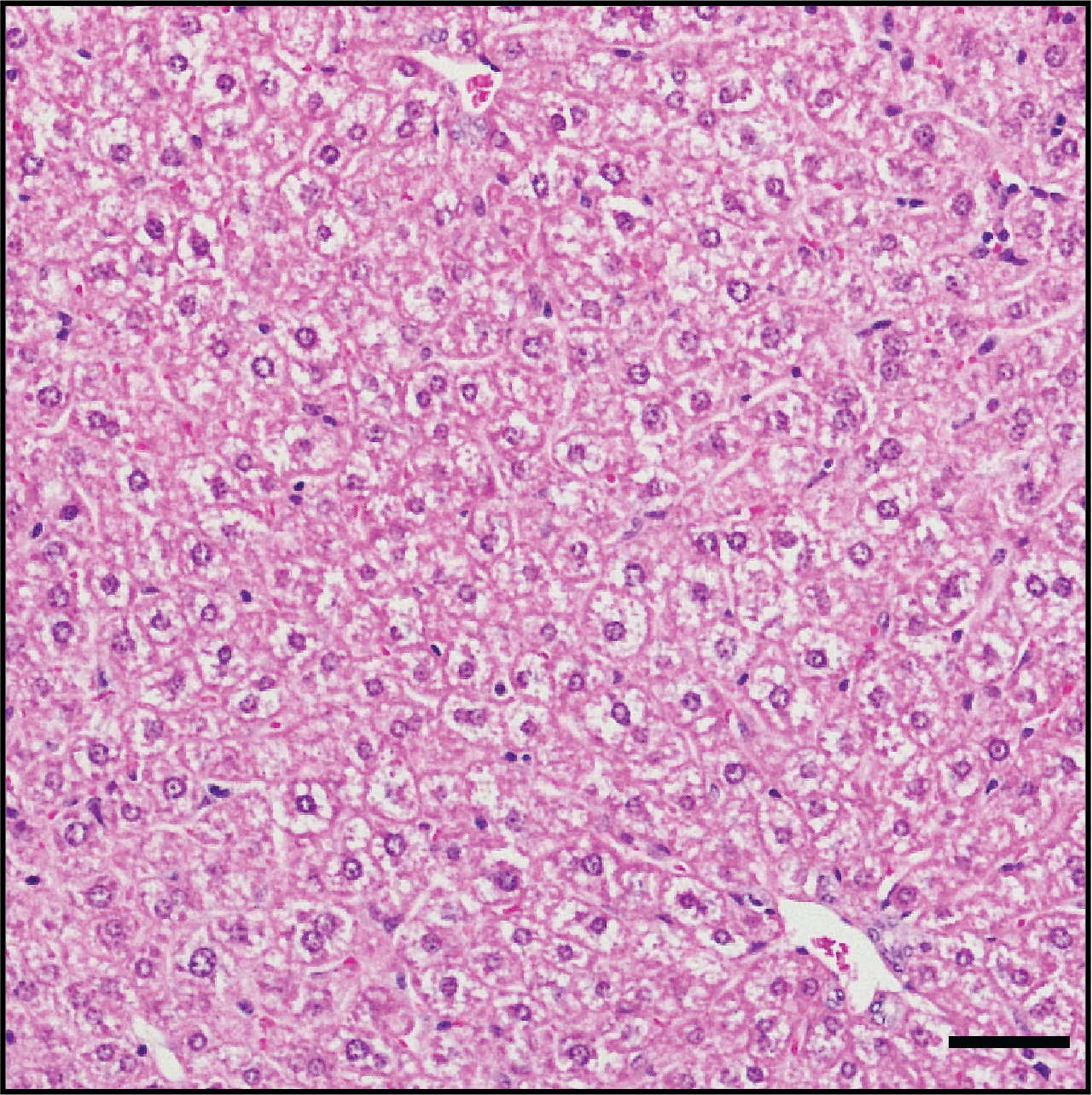

Supplement: Supplementary file 7 — Source Data for Figure 1 [file EMMM-15-e17694-s015.zip › Figure 1/1E/1E M-CSF d8.tiff]

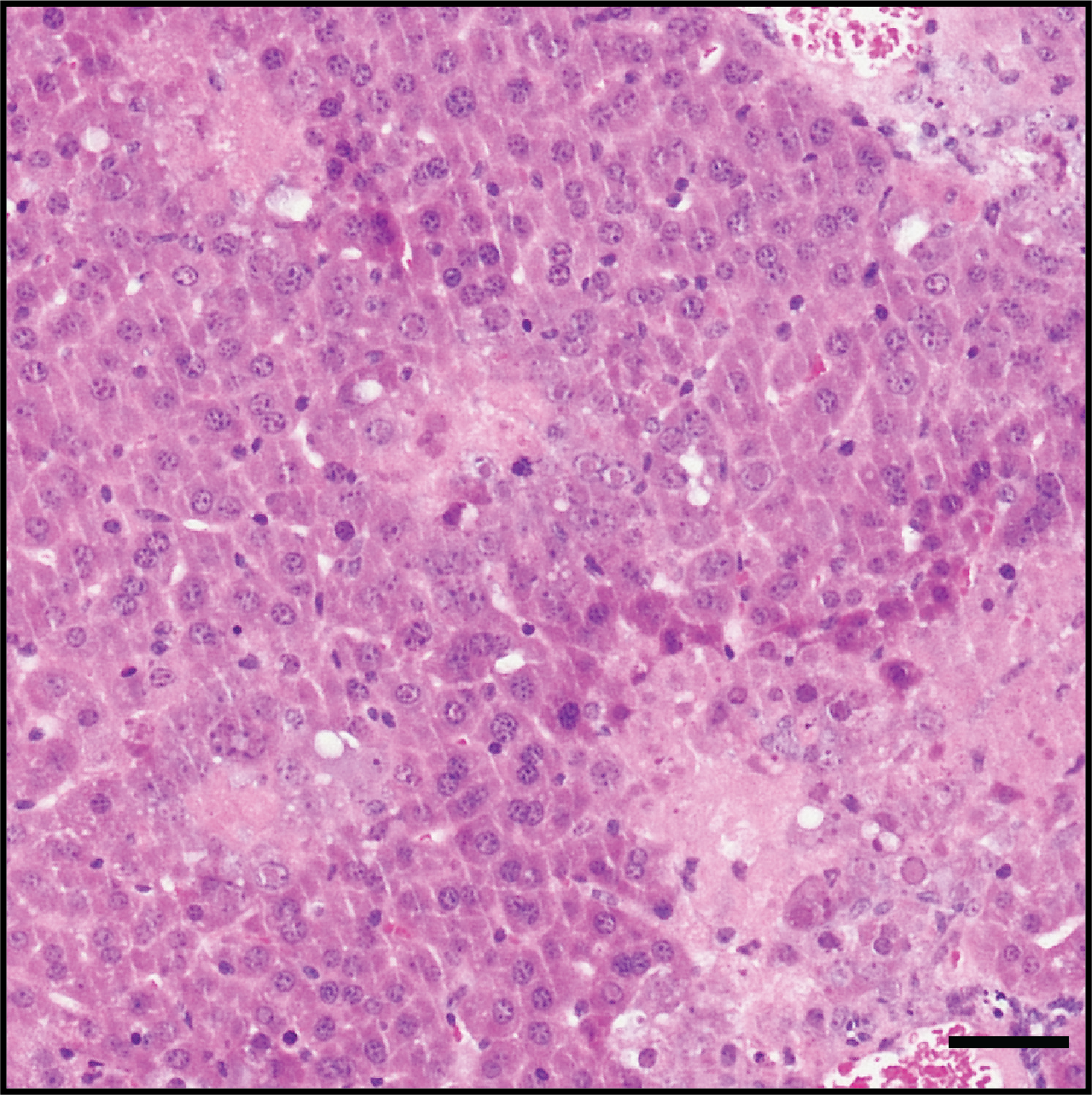

Supplement: Supplementary file 7 — Source Data for Figure 1 [file EMMM-15-e17694-s015.zip › Figure 1/1E/1E PBS d8.tiff]

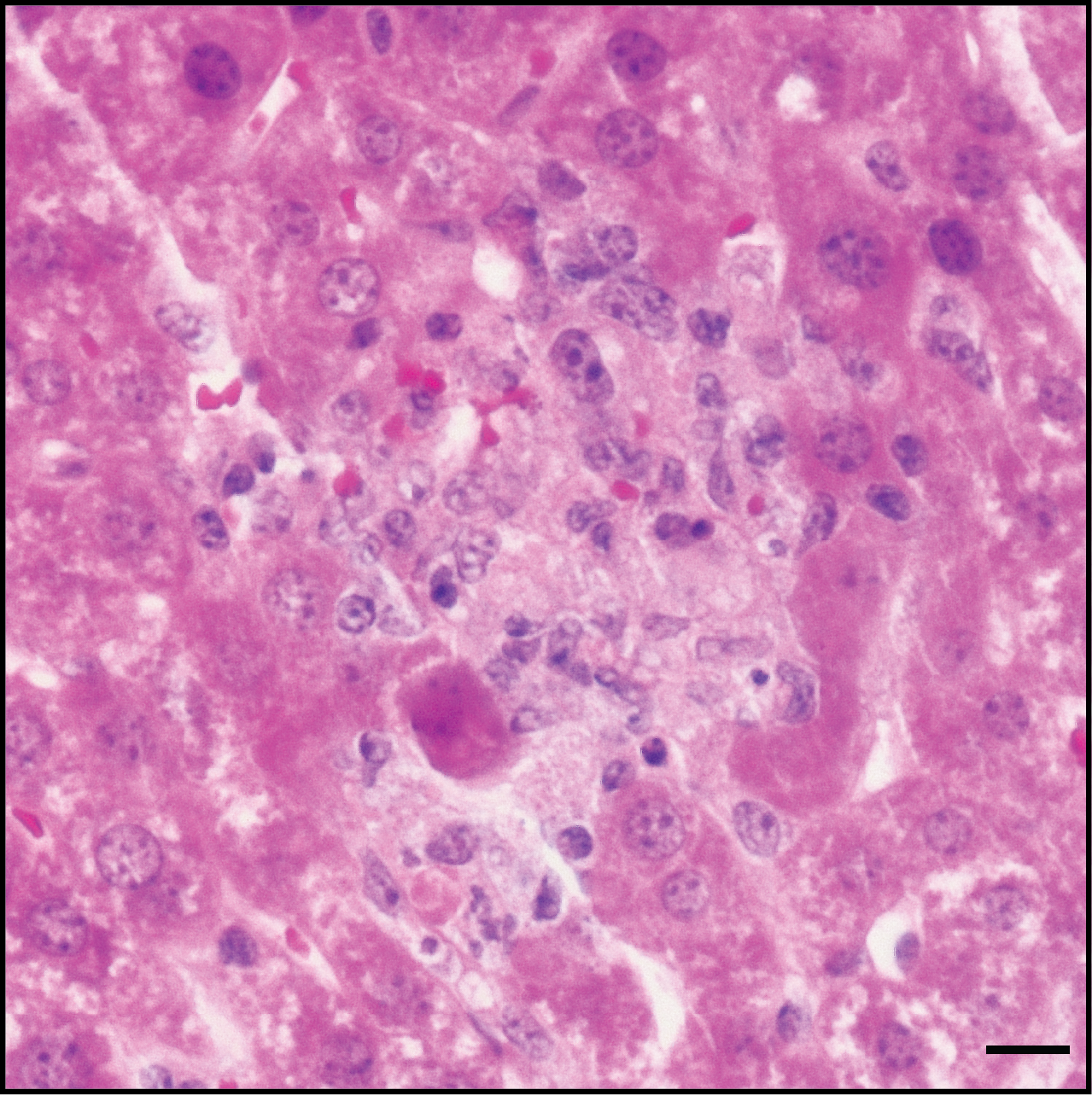

Supplement: Supplementary file 7 — Source Data for Figure 1 [file EMMM-15-e17694-s015.zip › Figure 1/1D/1D Necrotic cells.tiff]

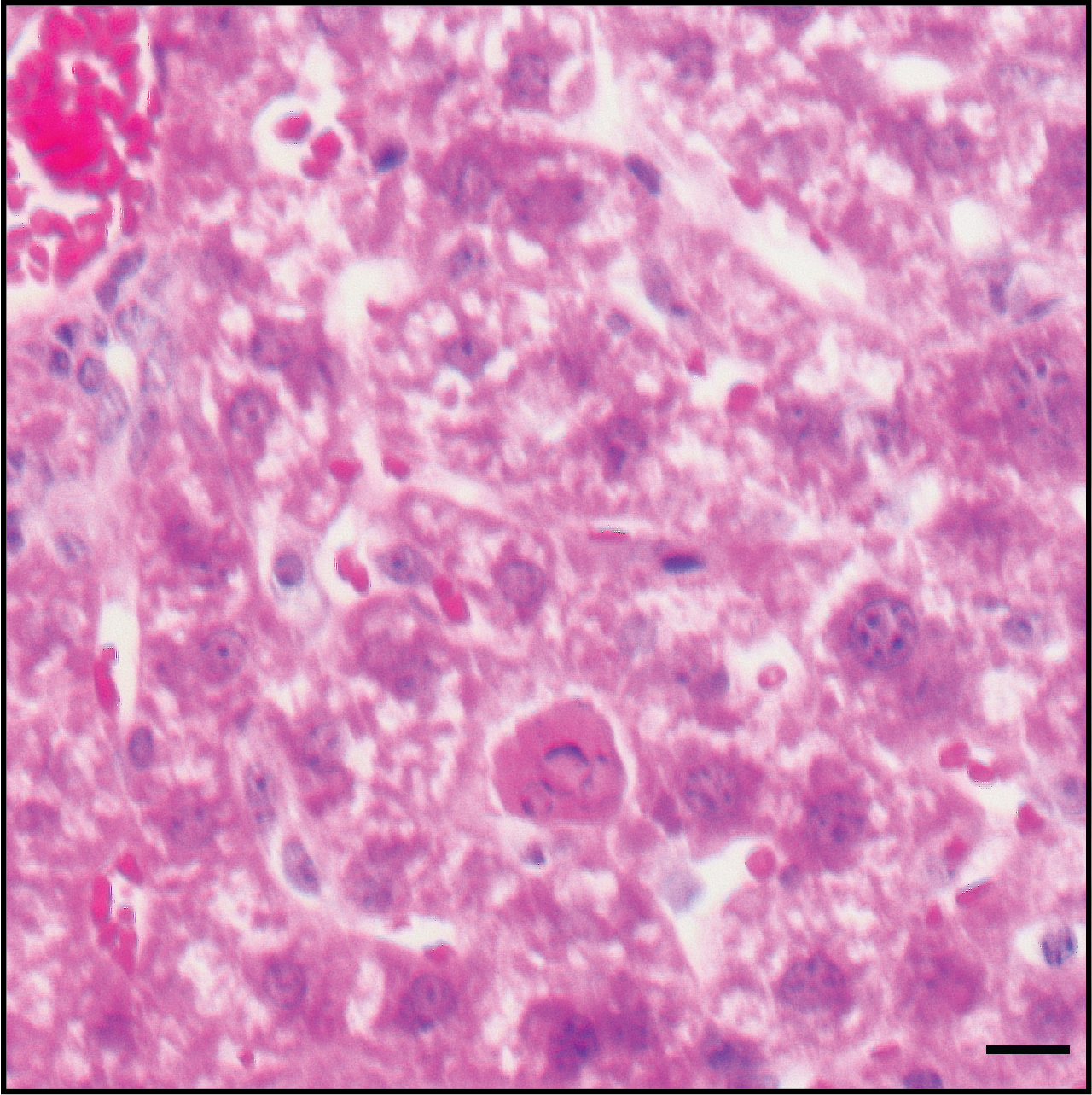

Supplement: Supplementary file 7 — Source Data for Figure 1 [file EMMM-15-e17694-s015.zip › Figure 1/1D/1D Apoptotic cells.tiff]

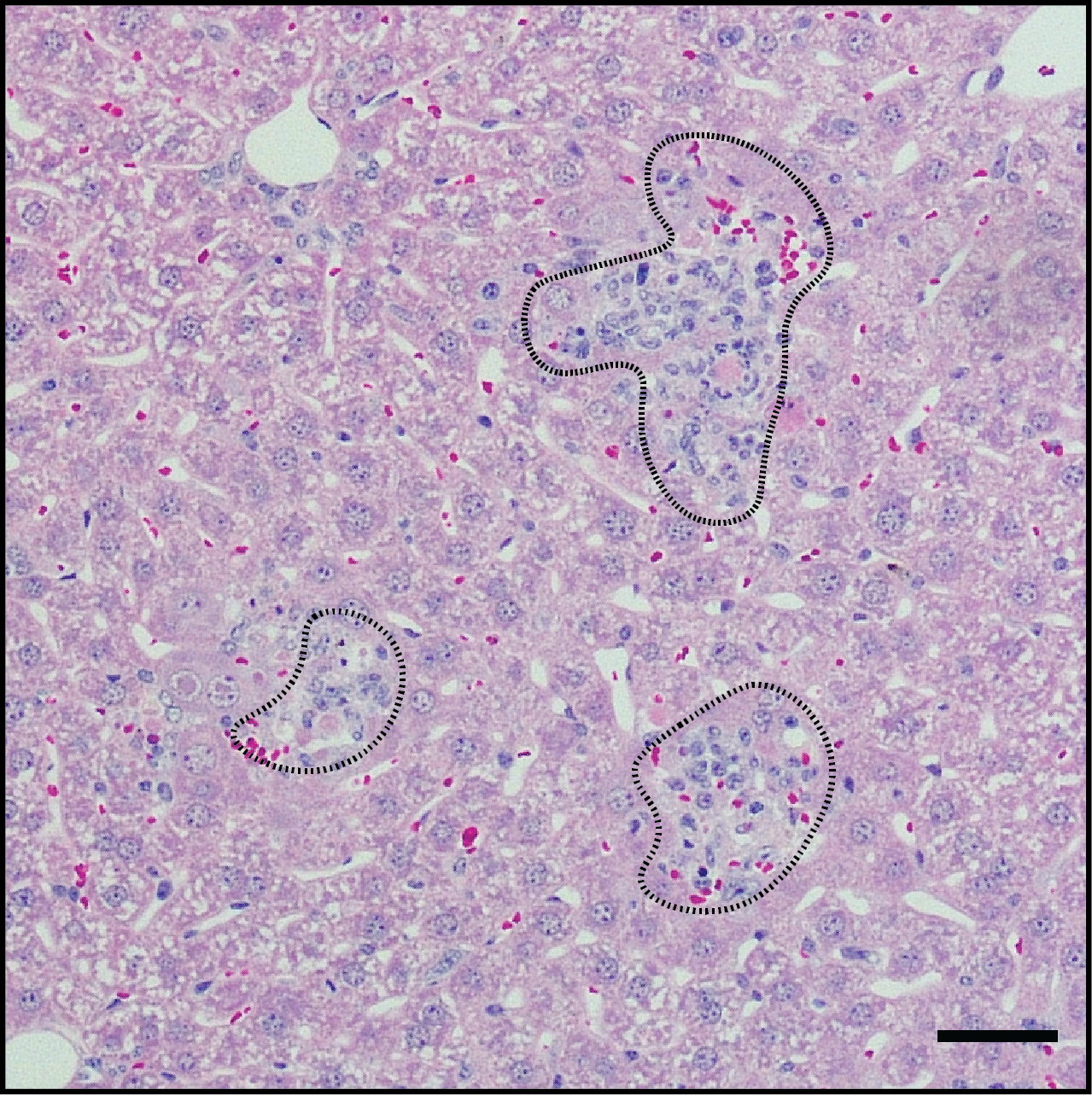

Supplement: Supplementary file 7 — Source Data for Figure 1 [file EMMM-15-e17694-s015.zip › Figure 1/1C/1C M-CSF.tiff]

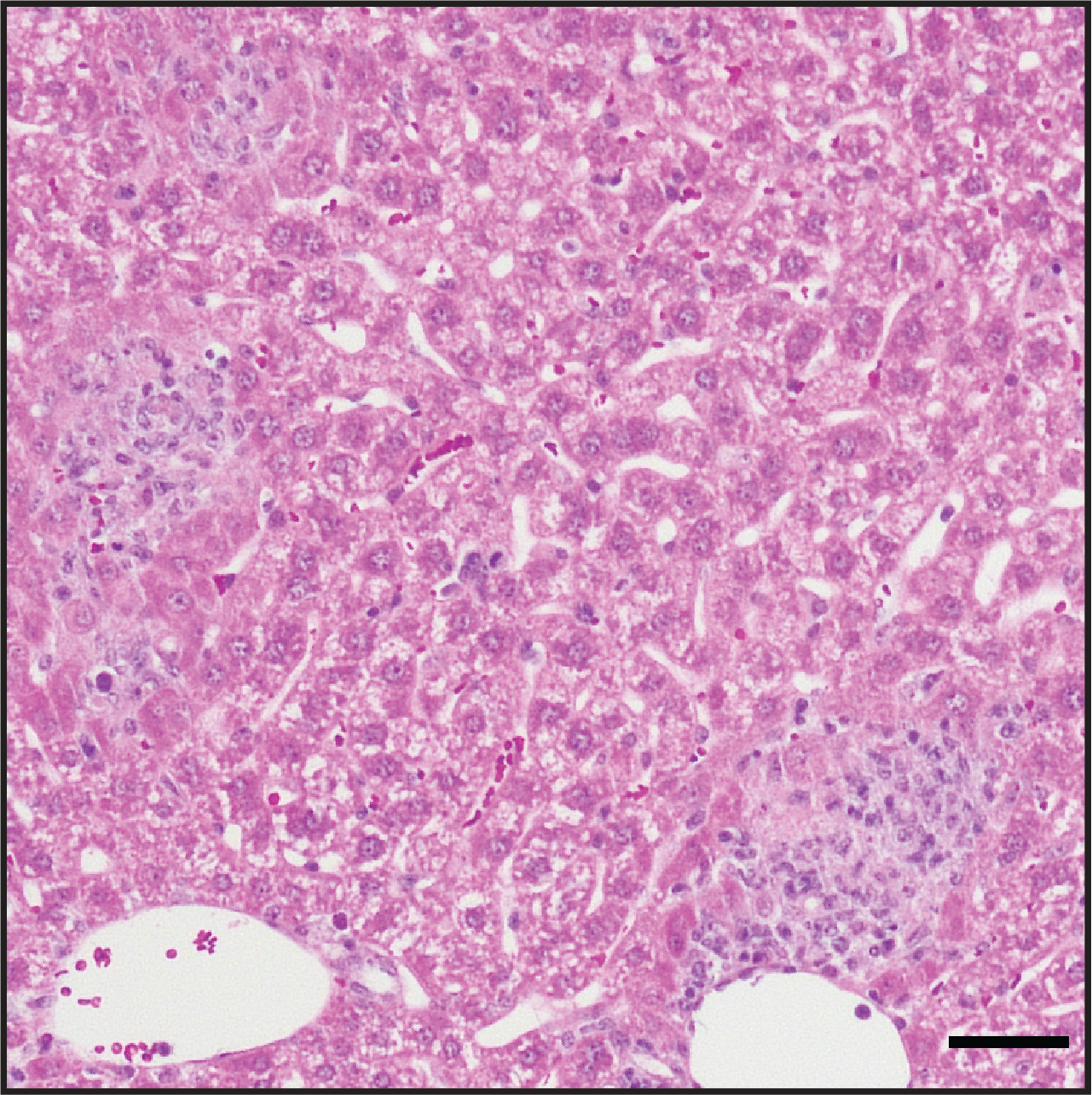

Supplement: Supplementary file 7 — Source Data for Figure 1 [file EMMM-15-e17694-s015.zip › Figure 1/1C/1C PBS.tiff]

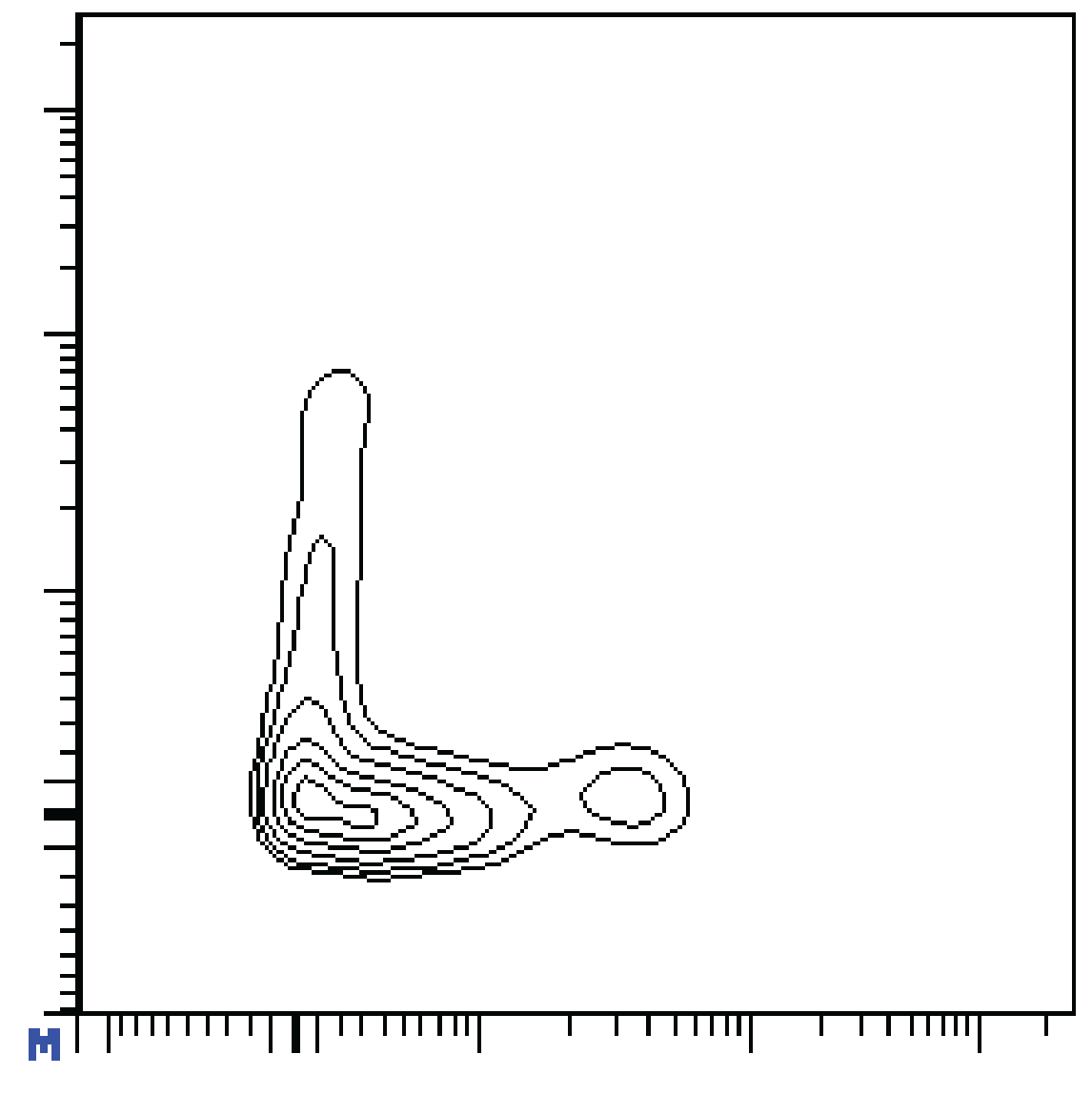

Supplement: Supplementary file 8 — Source Data for Figure 2 [file EMMM-15-e17694-s013.zip › Figure 2/2A/2A MCSF, no MCMV.tiff]

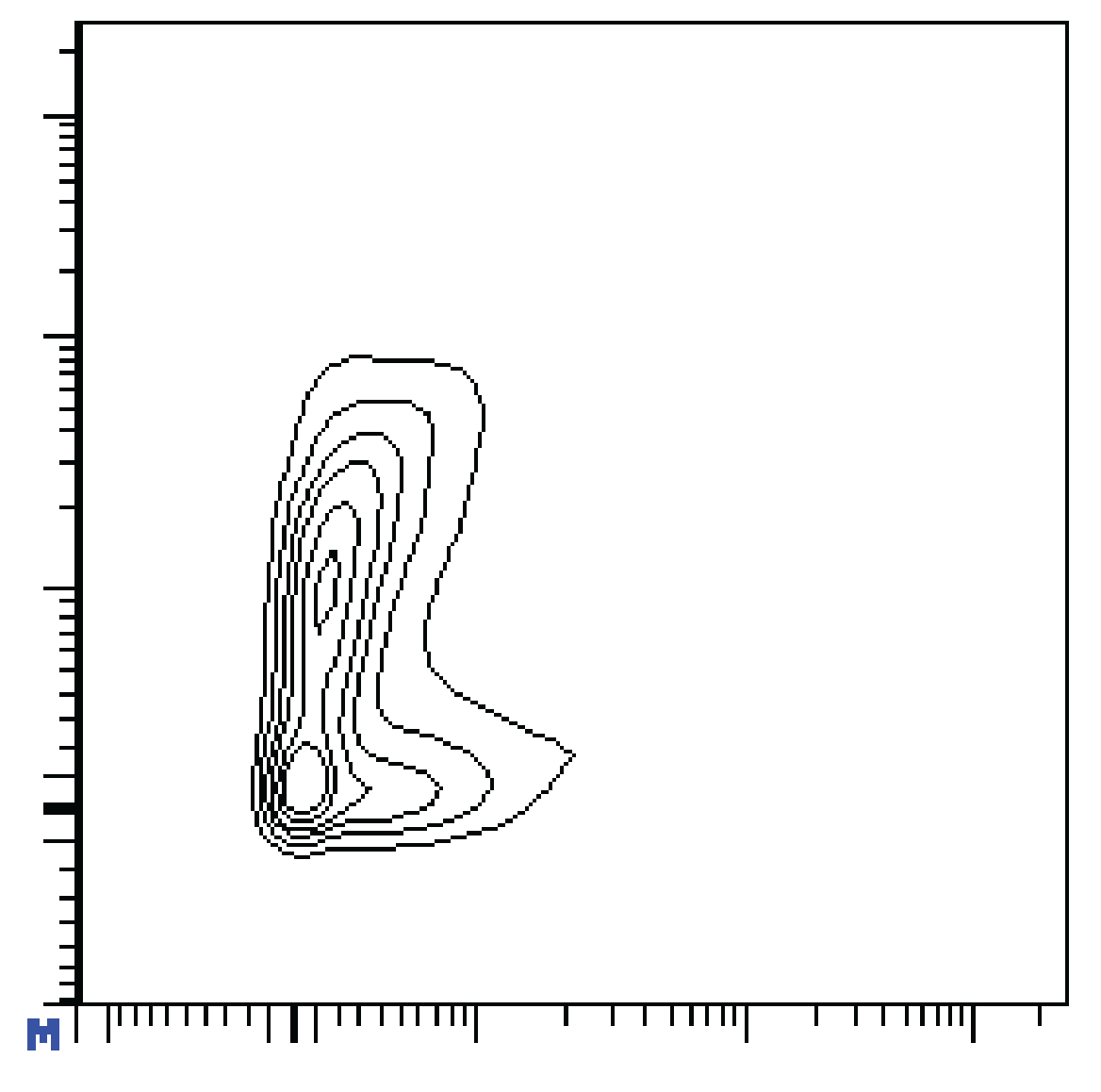

Supplement: Supplementary file 8 — Source Data for Figure 2 [file EMMM-15-e17694-s013.zip › Figure 2/2A/2A MCSF, MCMV.tiff]

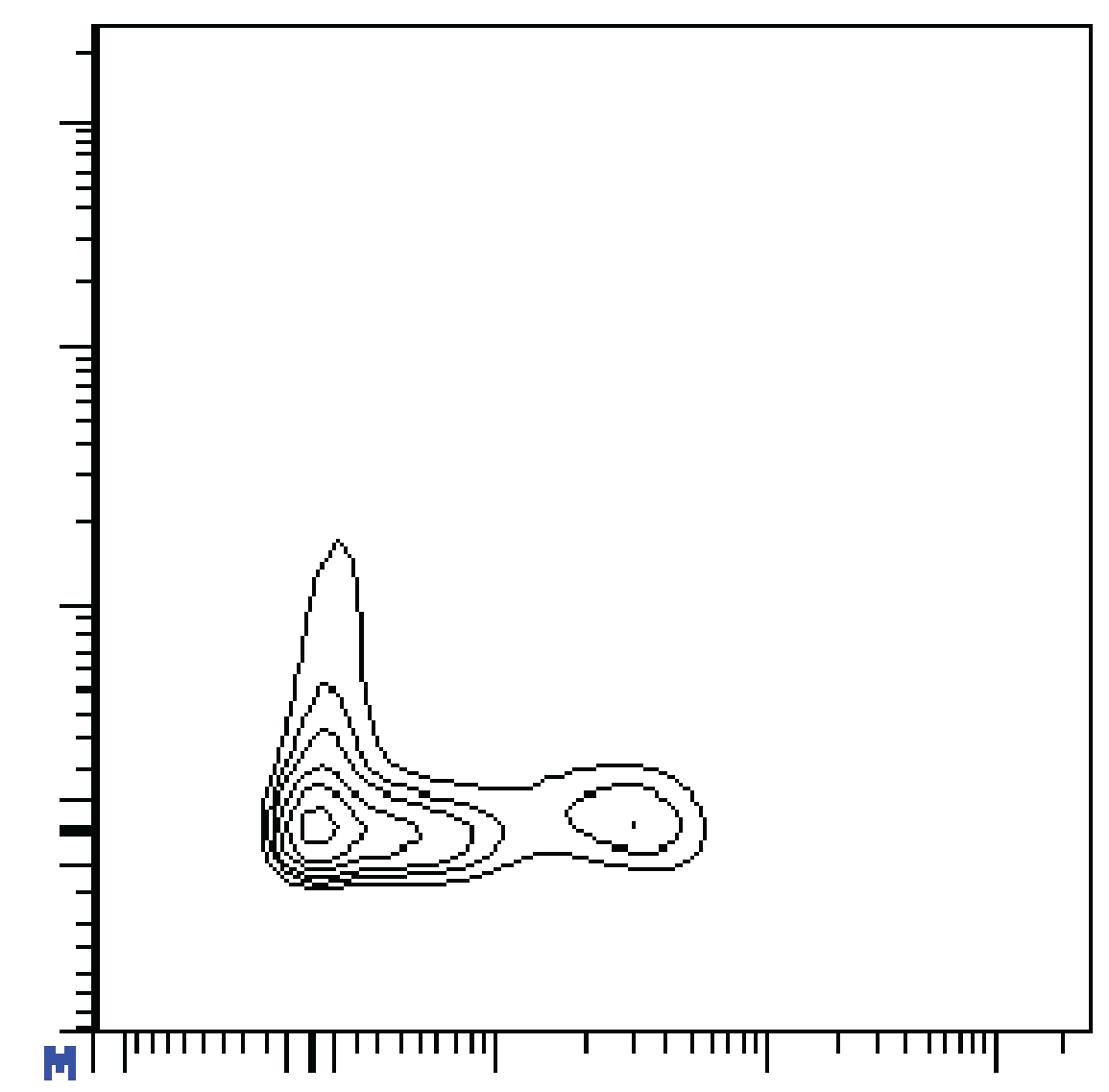

Supplement: Supplementary file 8 — Source Data for Figure 2 [file EMMM-15-e17694-s013.zip › Figure 2/2A/2A no MCSF, no MCMV.tiff]

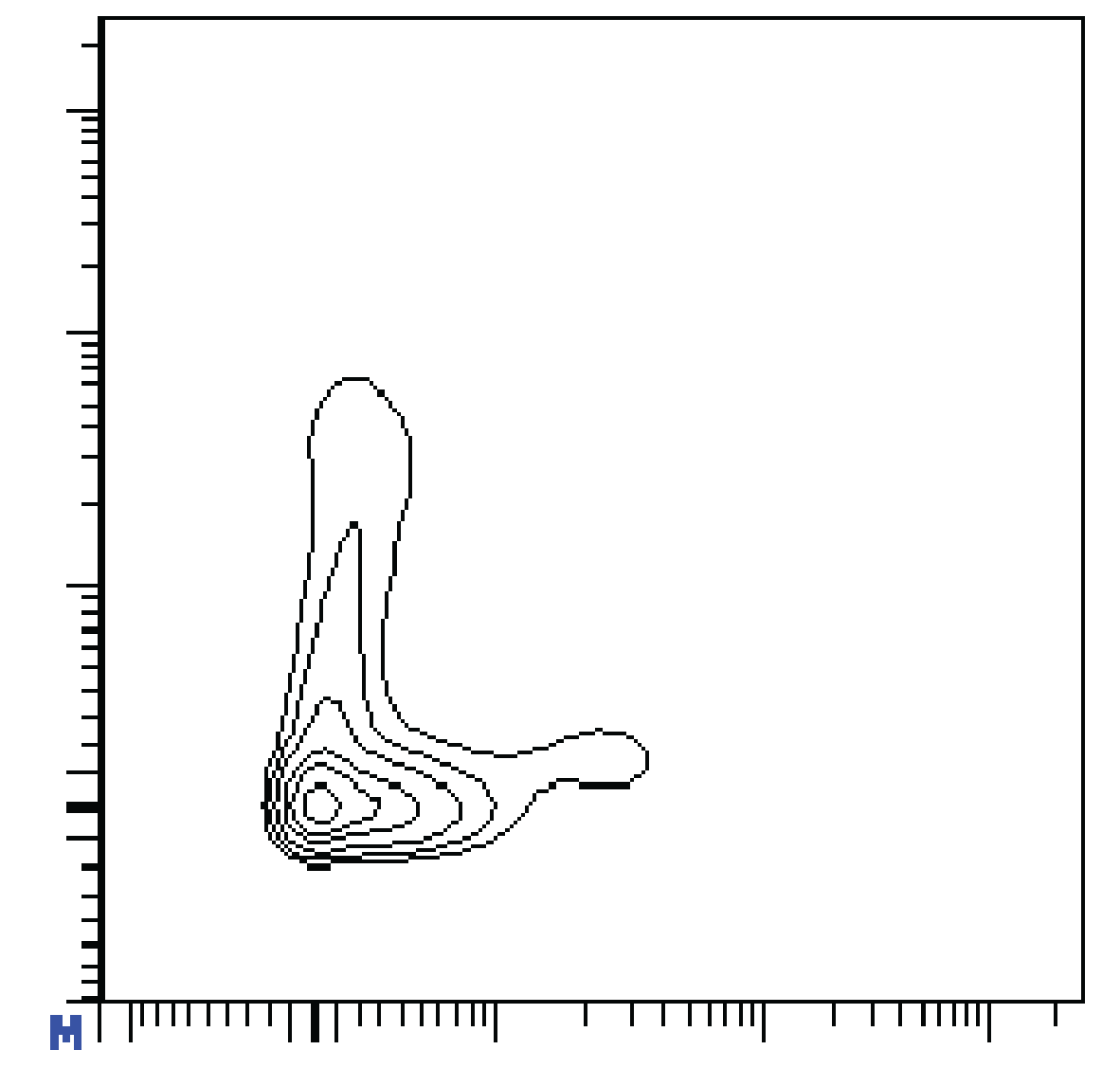

Supplement: Supplementary file 8 — Source Data for Figure 2 [file EMMM-15-e17694-s013.zip › Figure 2/2A/2A no MCSF, MCMV.tiff]

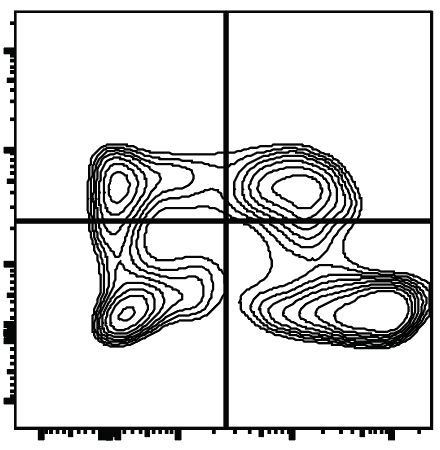

Supplement: Supplementary file 8 — Source Data for Figure 2 [file EMMM-15-e17694-s013.zip › Figure 2/2D/2D MCSF, no MCMV.tiff]

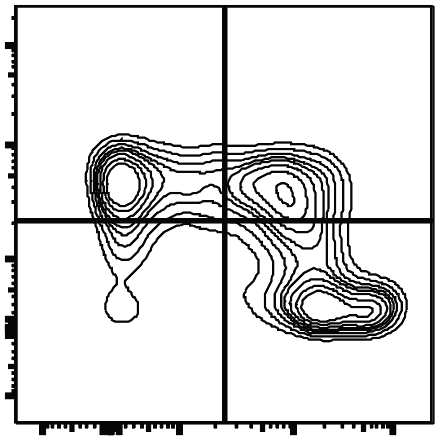

Supplement: Supplementary file 8 — Source Data for Figure 2 [file EMMM-15-e17694-s013.zip › Figure 2/2D/2D MCSF, MCMV.tiff]

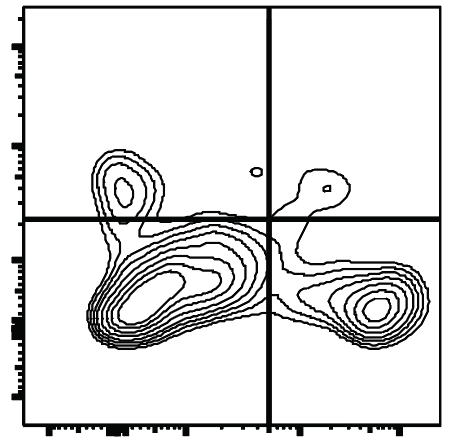

Supplement: Supplementary file 8 — Source Data for Figure 2 [file EMMM-15-e17694-s013.zip › Figure 2/2D/2D no MCSF no MCMV.tiff]

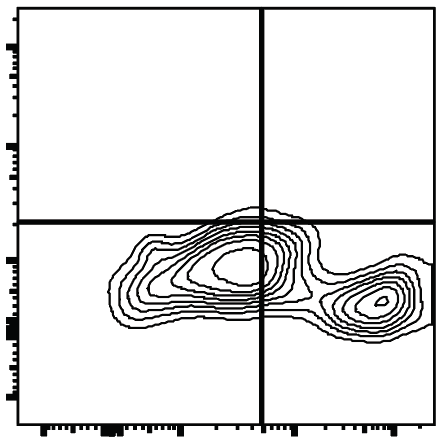

Supplement: Supplementary file 8 — Source Data for Figure 2 [file EMMM-15-e17694-s013.zip › Figure 2/2D/2D no MCSF, MCMV.tiff]

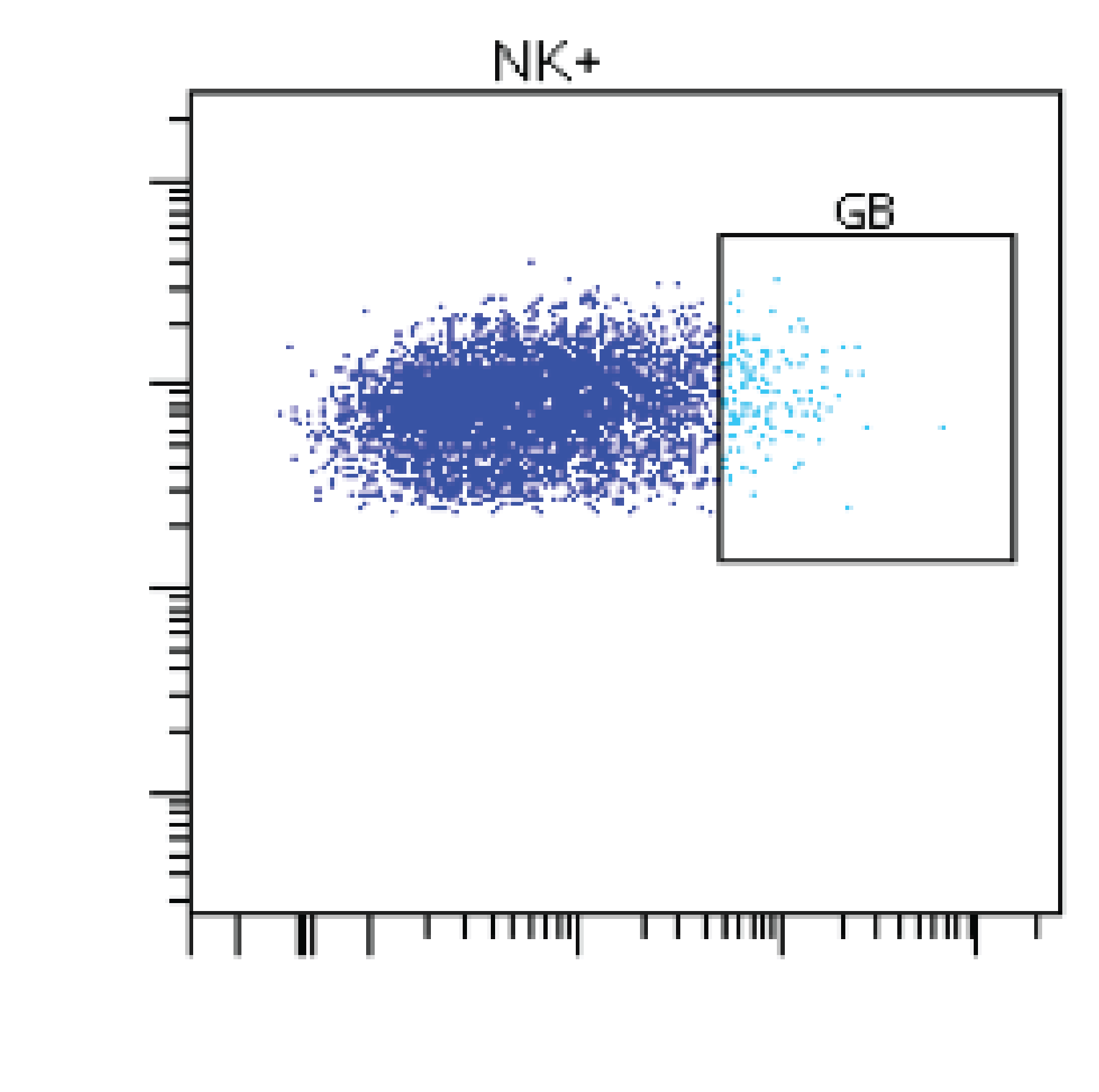

Supplement: Supplementary file 9 — Source Data for Figure 3 [file EMMM-15-e17694-s001.zip › Figure 3/3B/3B no MCSF.tiff]

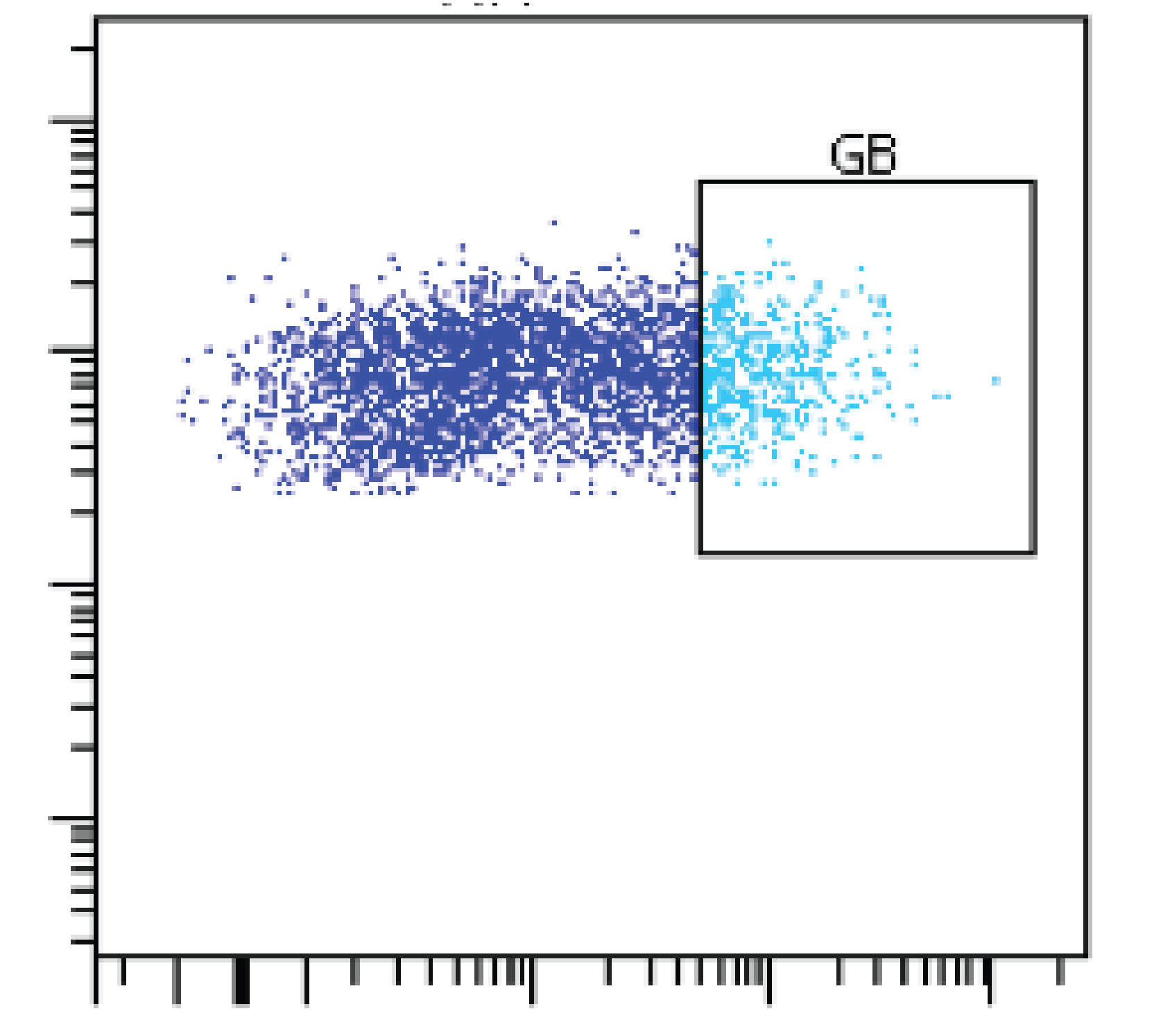

Supplement: Supplementary file 9 — Source Data for Figure 3 [file EMMM-15-e17694-s001.zip › Figure 3/3B/3B with MCSF.tiff]

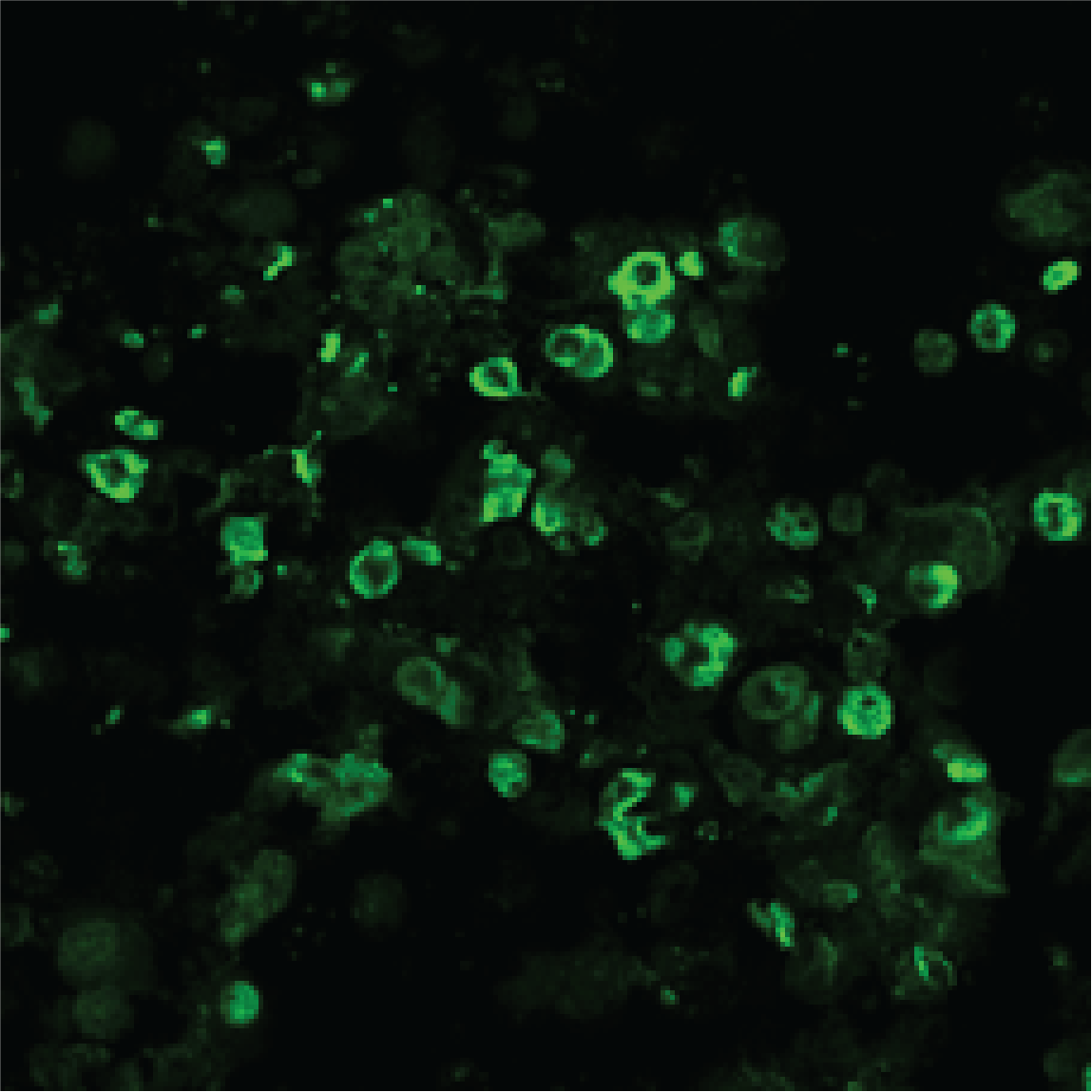

Supplement: Supplementary file 9 — Source Data for Figure 3 [file EMMM-15-e17694-s001.zip › Figure 3/3D/3D no MCSF NK1.1.tiff]

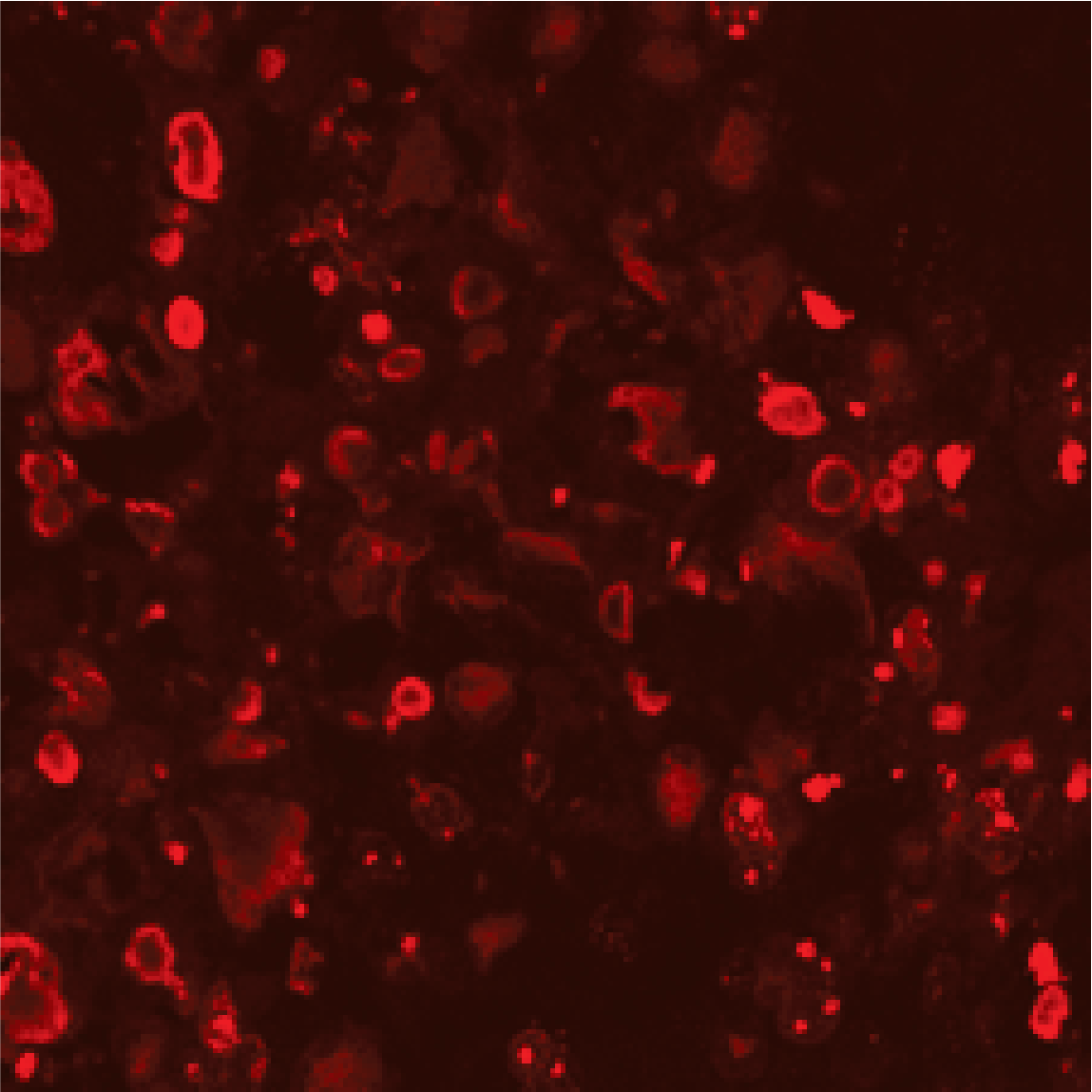

Supplement: Supplementary file 9 — Source Data for Figure 3 [file EMMM-15-e17694-s001.zip › Figure 3/3D/3D no MCSF MCMV IE1.tiff]

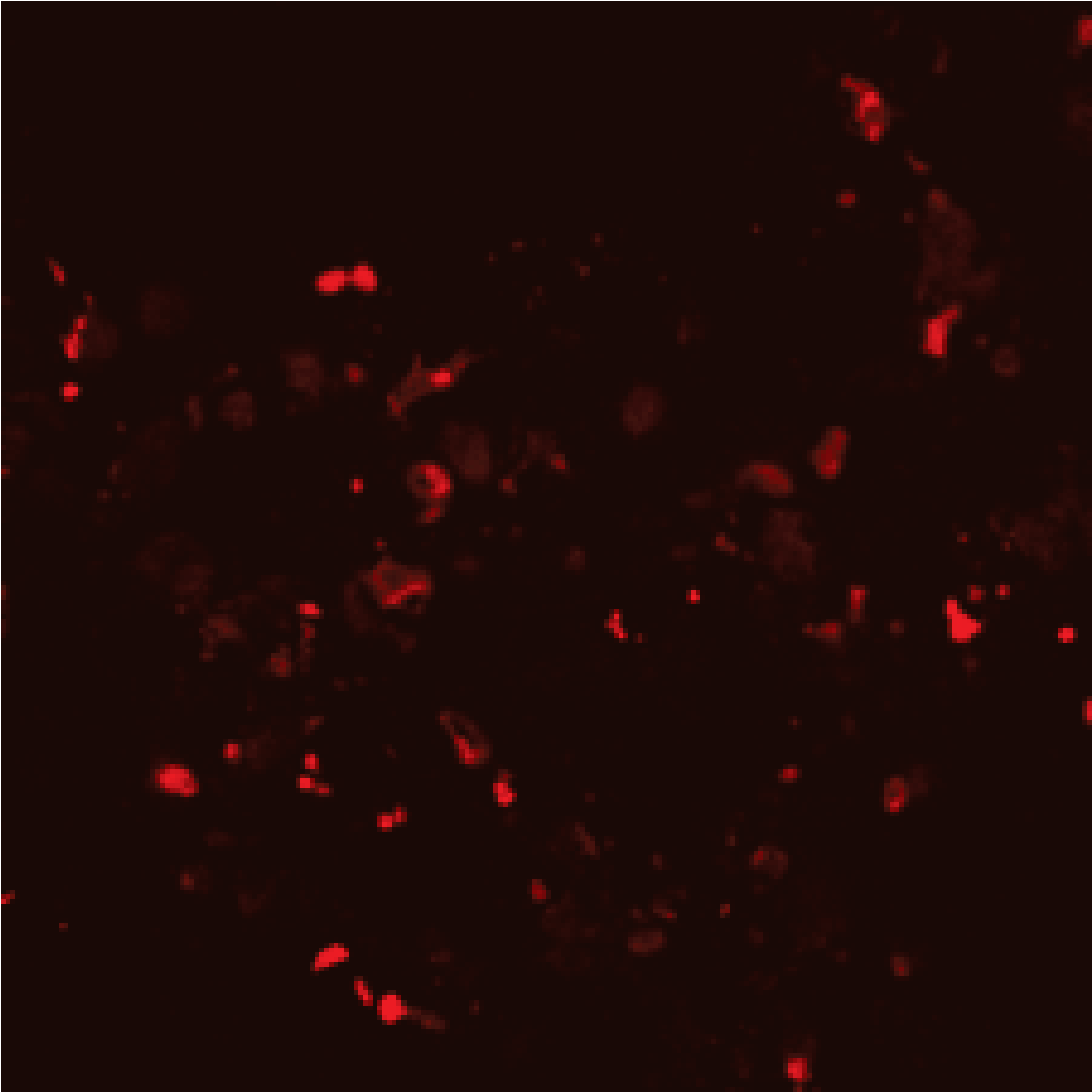

Supplement: Supplementary file 9 — Source Data for Figure 3 [file EMMM-15-e17694-s001.zip › Figure 3/3D/3D with MCSF MCMV IE1.tiff]

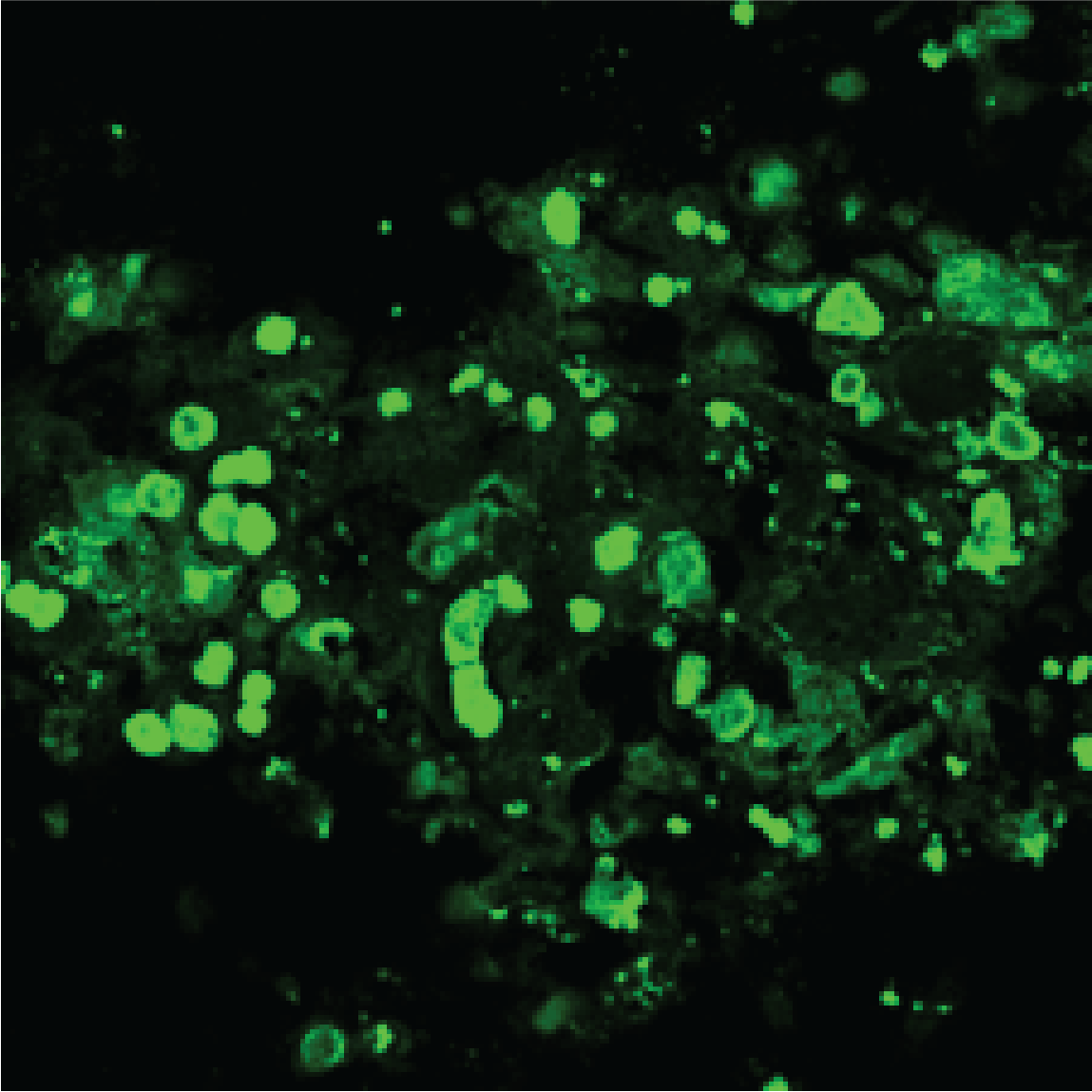

Supplement: Supplementary file 9 — Source Data for Figure 3 [file EMMM-15-e17694-s001.zip › Figure 3/3D/3D with MCSF NK1.1.tiff]

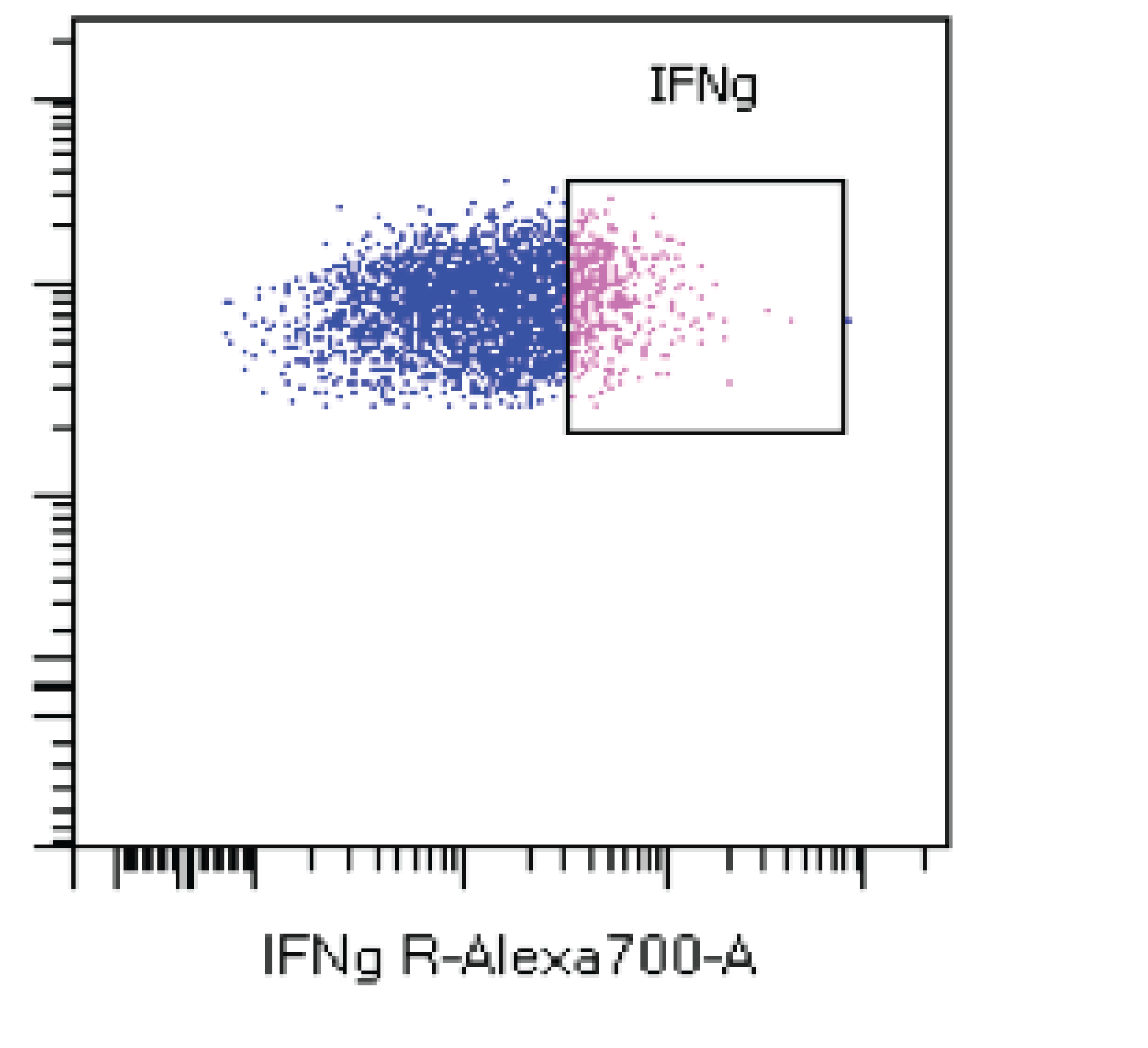

Supplement: Supplementary file 9 — Source Data for Figure 3 [file EMMM-15-e17694-s001.zip › Figure 3/3A/3A with MCSF.tiff]

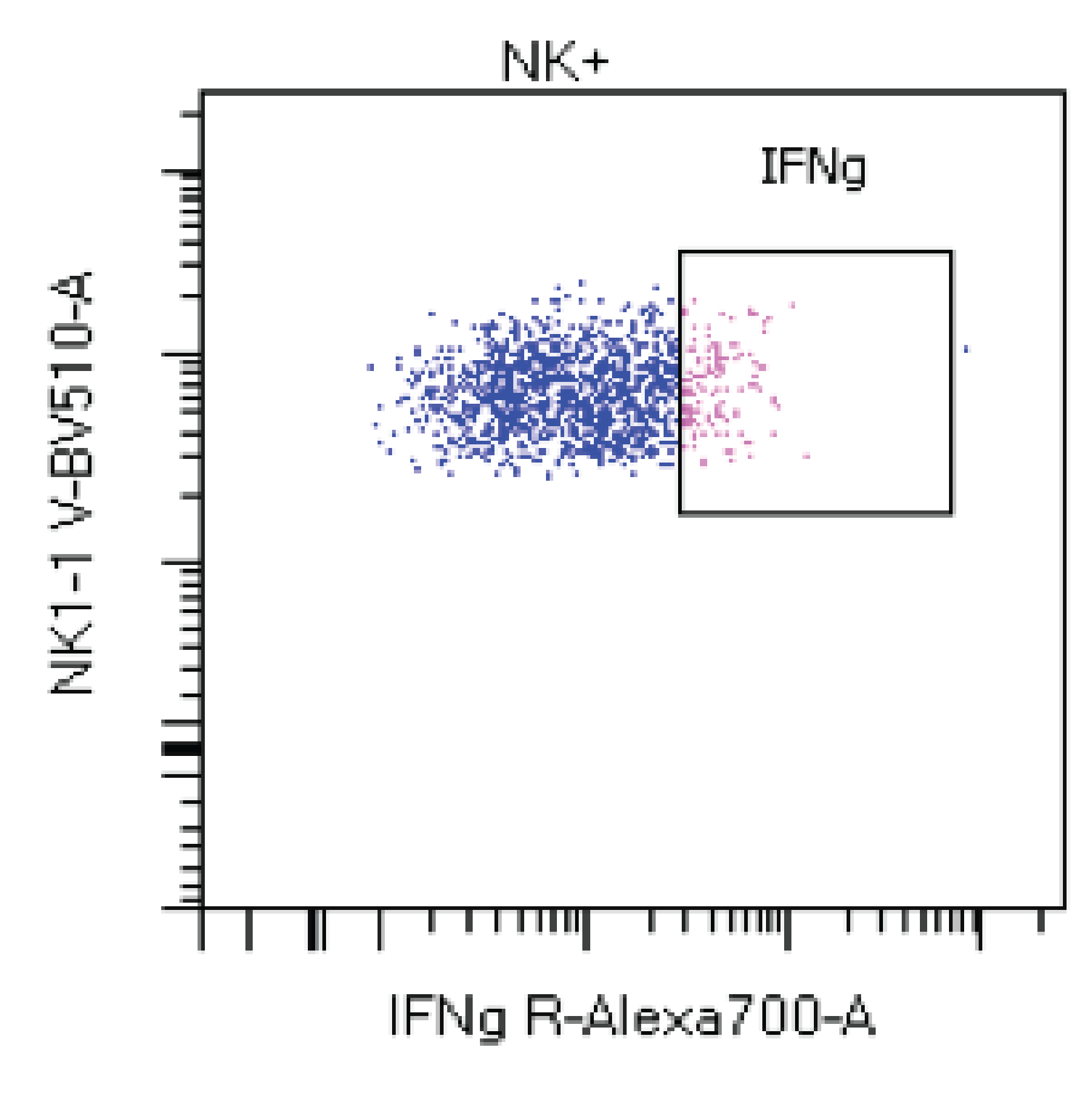

Supplement: Supplementary file 9 — Source Data for Figure 3 [file EMMM-15-e17694-s001.zip › Figure 3/3A/3A no MCSF.tiff]

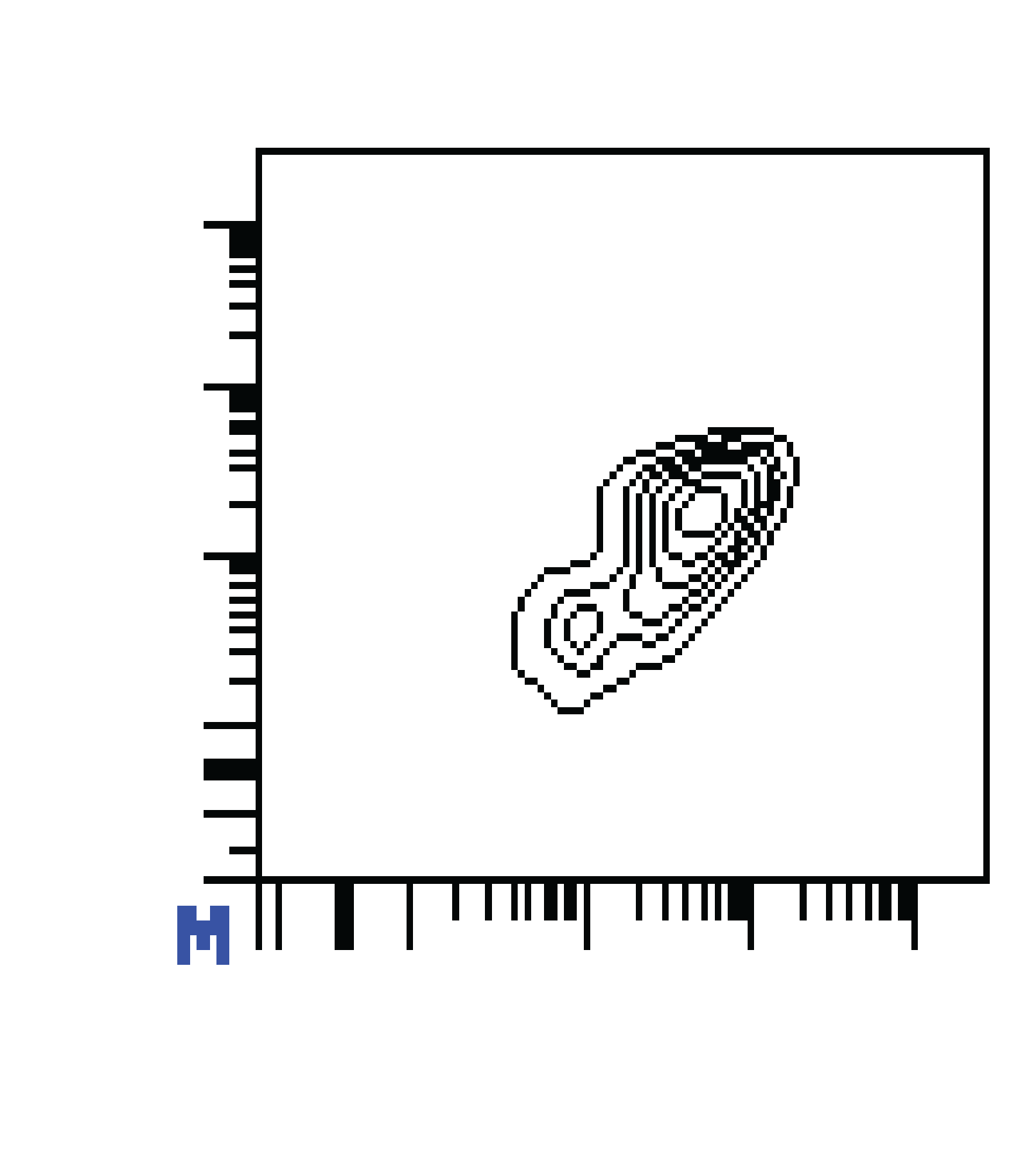

Supplement: Supplementary file 10 — Source Data for Figure 4 [file EMMM-15-e17694-s011.zip › Figure 4/4A/4A d14 GMPs panel 2.tiff]

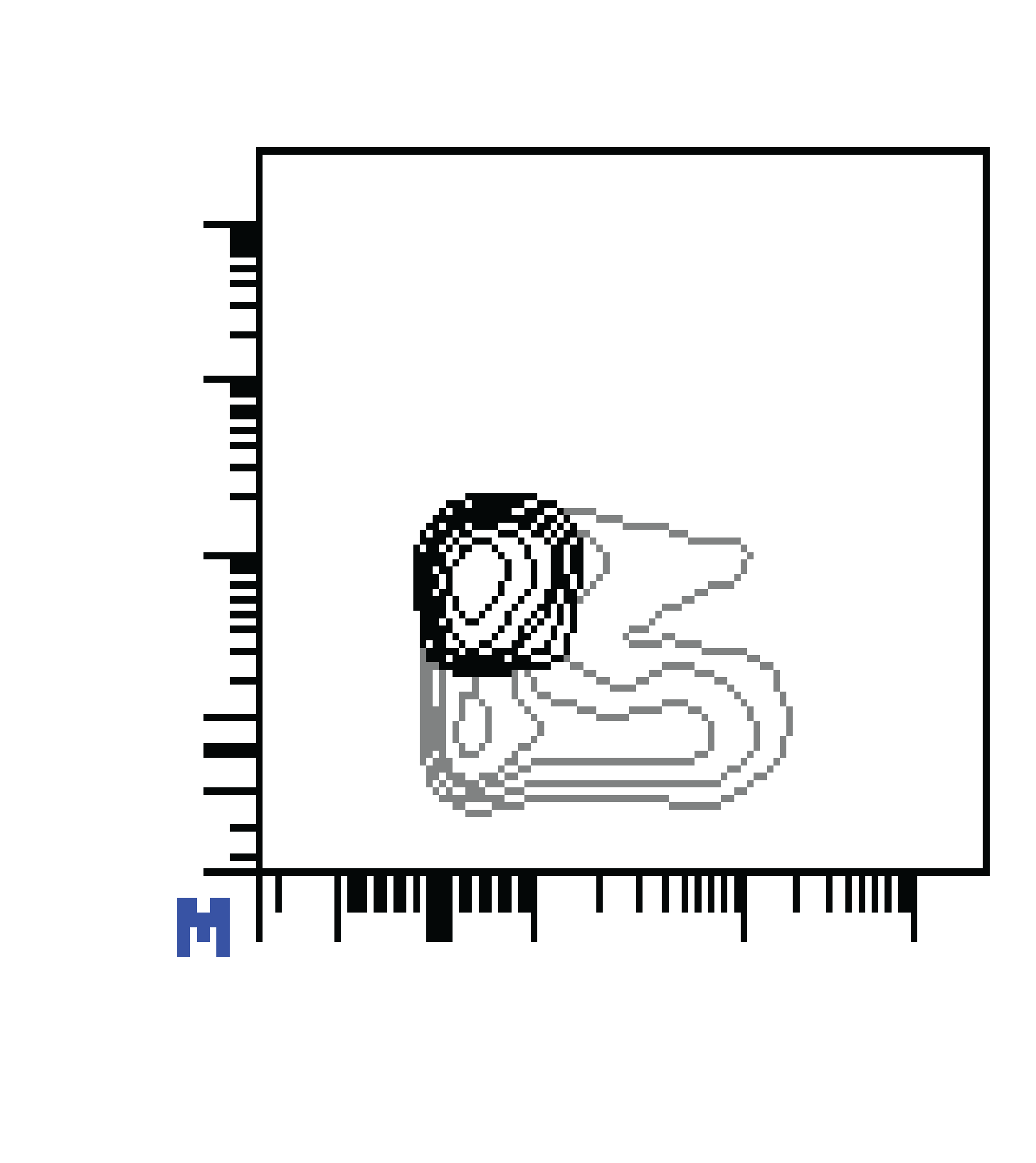

Supplement: Supplementary file 10 — Source Data for Figure 4 [file EMMM-15-e17694-s011.zip › Figure 4/4A/4A d14 GMPs panel 1.tiff]

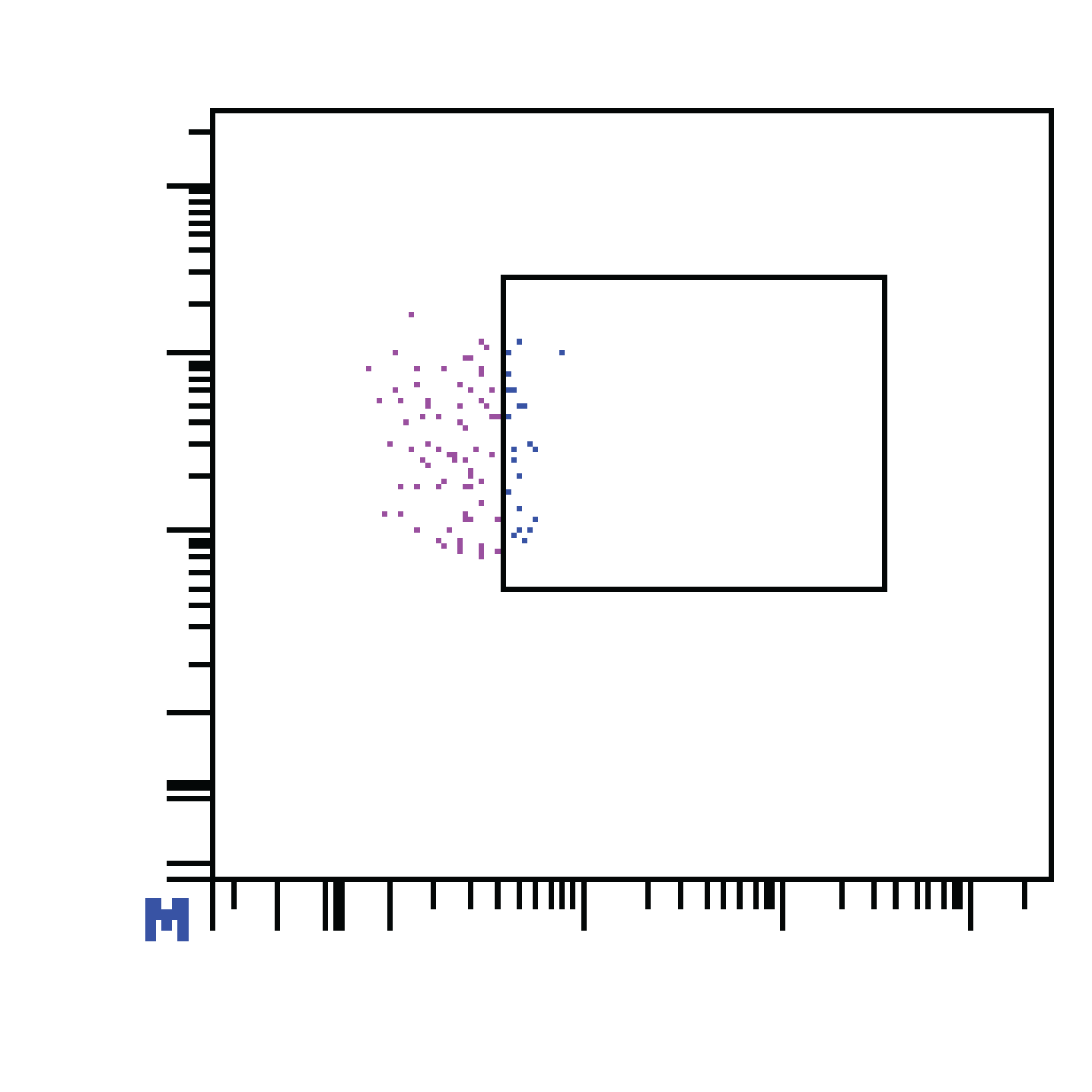

Supplement: Supplementary file 11 — Source Data for Figure 5 [file EMMM-15-e17694-s003.zip › Figure 5/5D/5D no MCSF no MCMV.tiff]

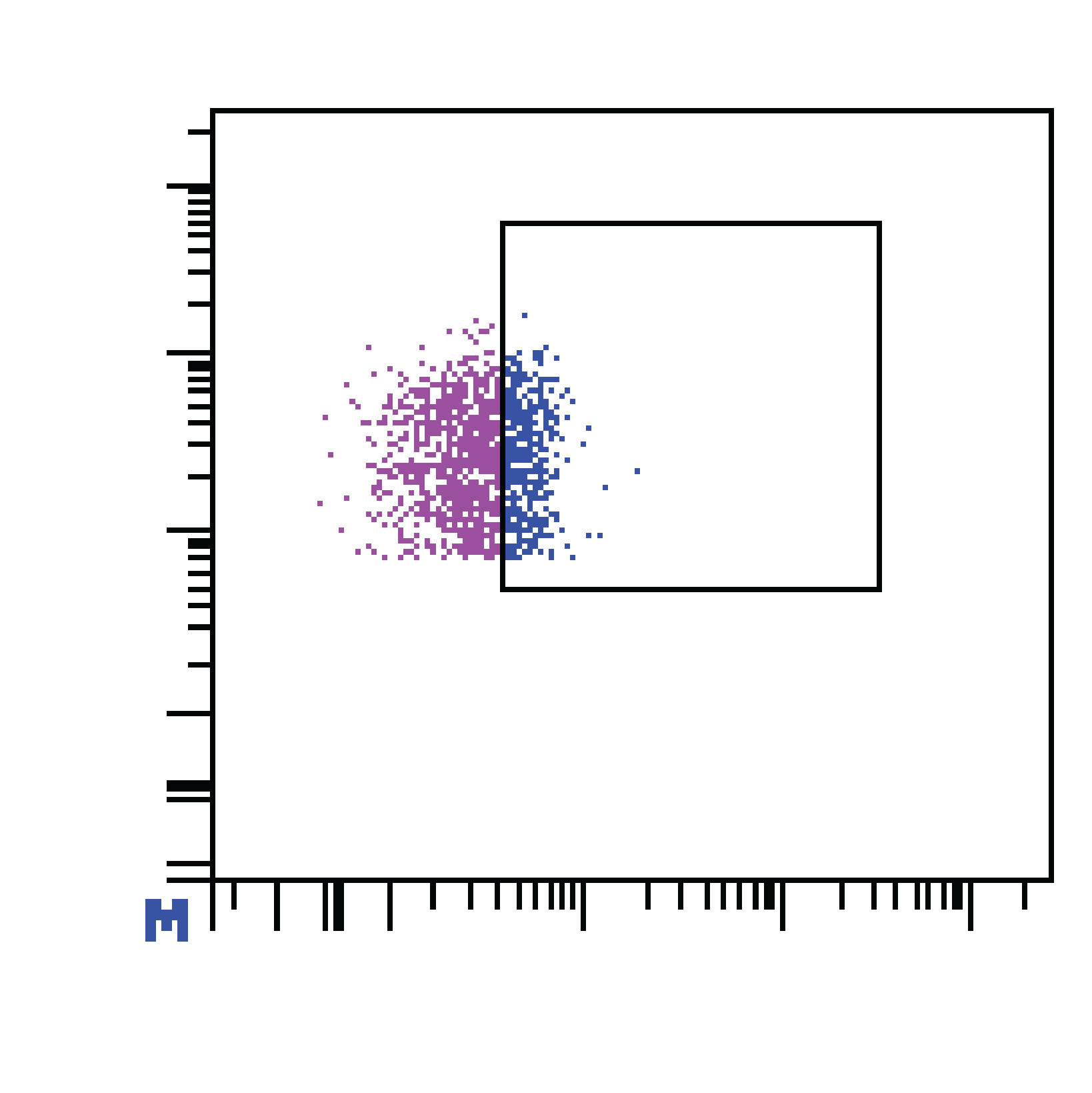

Supplement: Supplementary file 11 — Source Data for Figure 5 [file EMMM-15-e17694-s003.zip › Figure 5/5D/5D MCSF no MCMV.tiff]

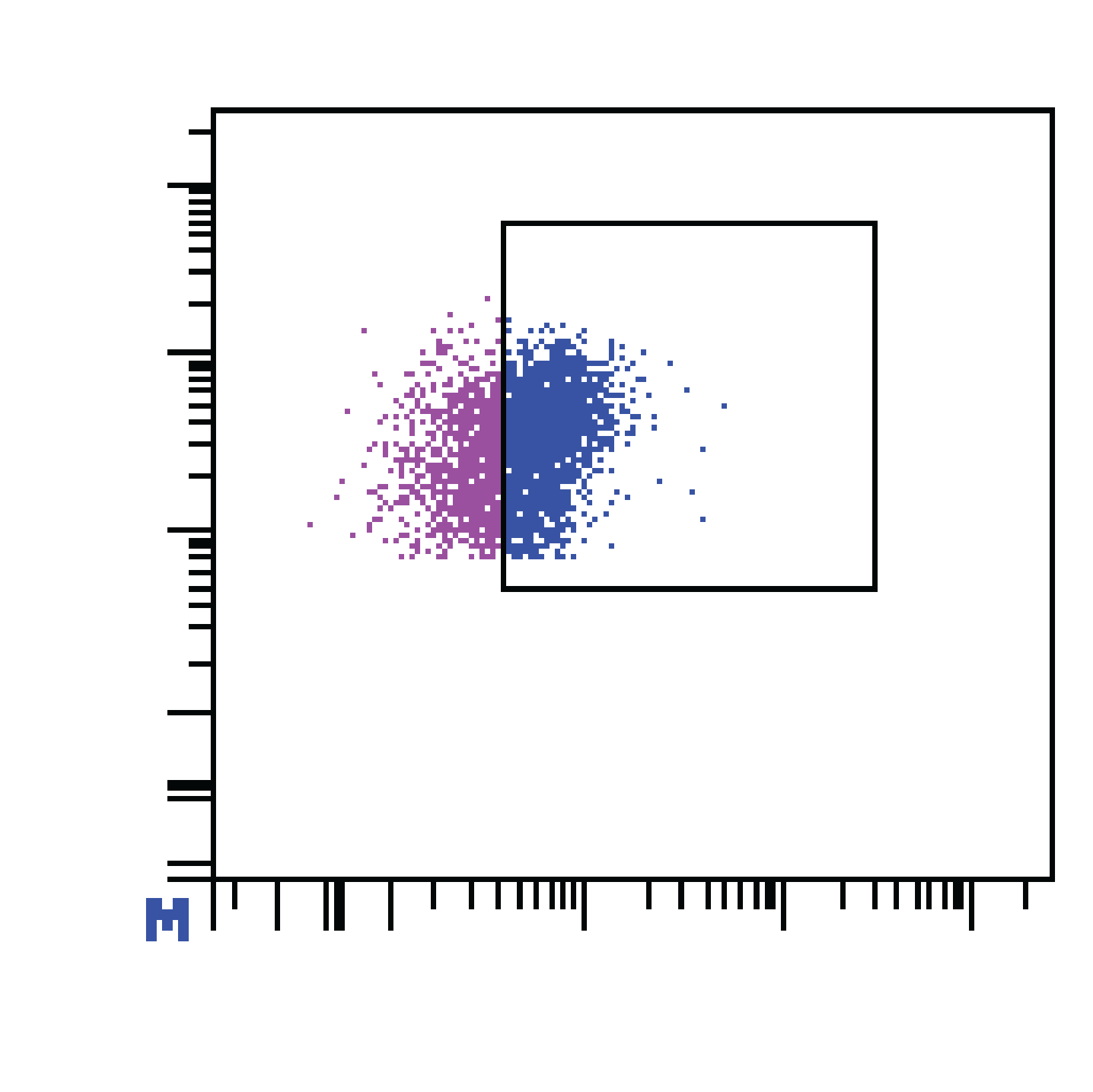

Supplement: Supplementary file 11 — Source Data for Figure 5 [file EMMM-15-e17694-s003.zip › Figure 5/5D/5D MCSF with MCMV.tiff]

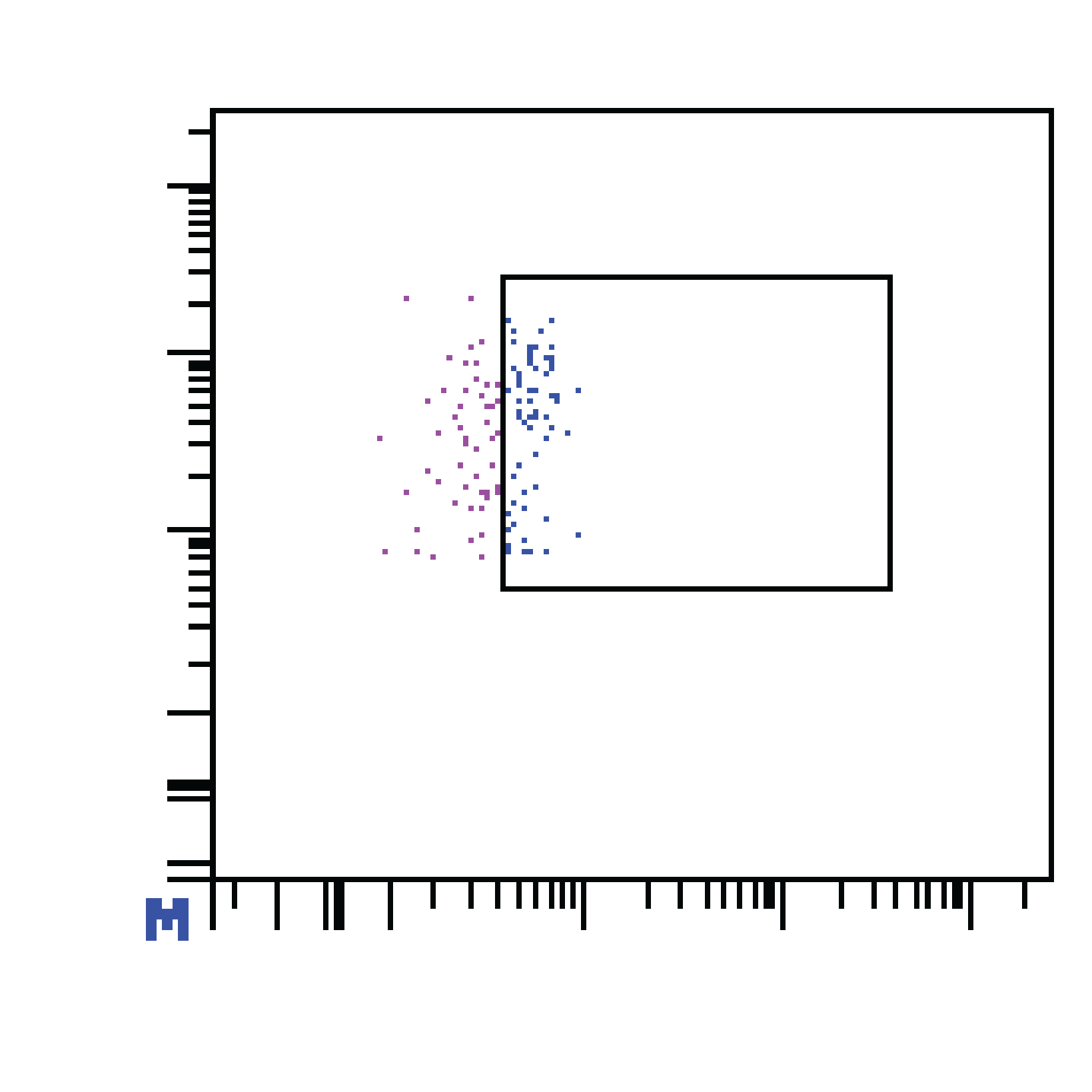

Supplement: Supplementary file 11 — Source Data for Figure 5 [file EMMM-15-e17694-s003.zip › Figure 5/5D/5D no MCSF with MCMV.tiff]

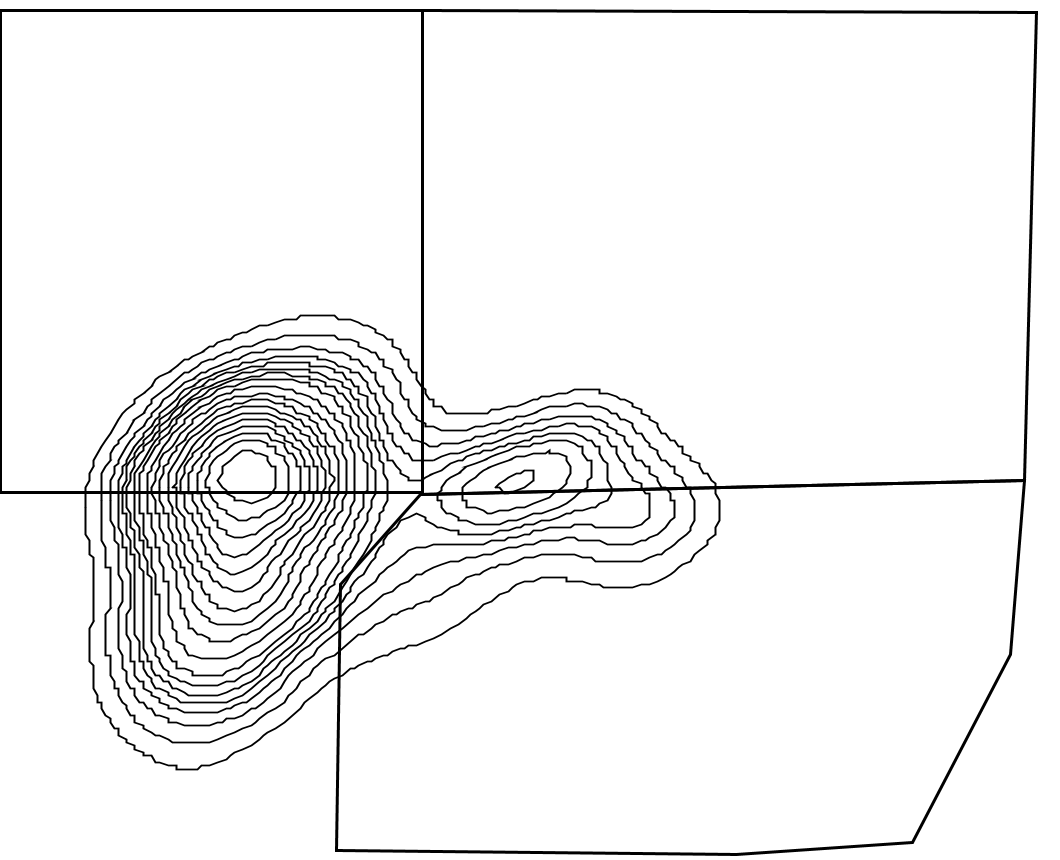

Supplement: Supplementary file 13 — Source Data for Figure 7 [file EMMM-15-e17694-s004.zip › Figure 7/7F/7F GMP SCF d+5.tiff]

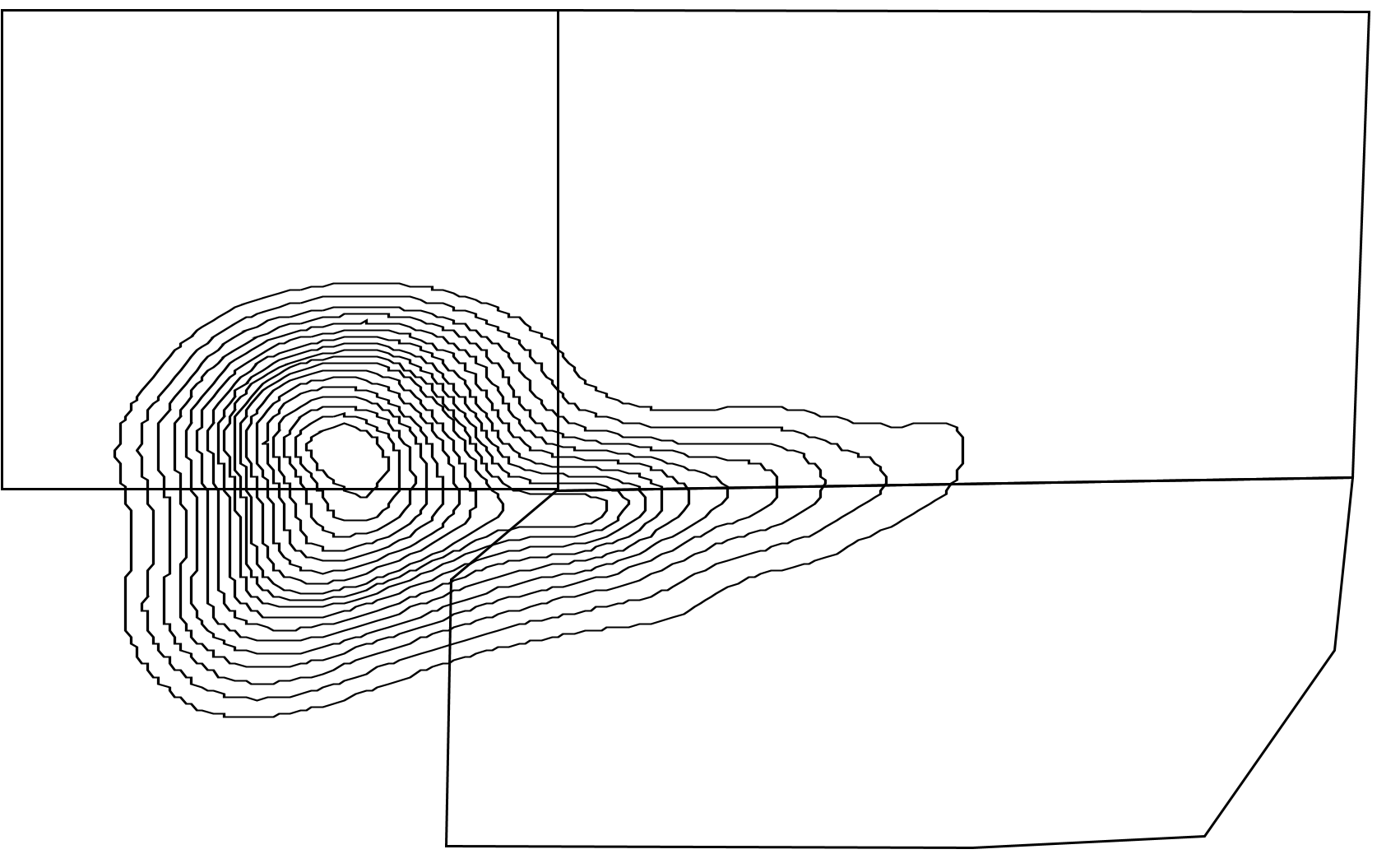

Supplement: Supplementary file 13 — Source Data for Figure 7 [file EMMM-15-e17694-s004.zip › Figure 7/7F/7F GMP IL3 d+5.tiff]

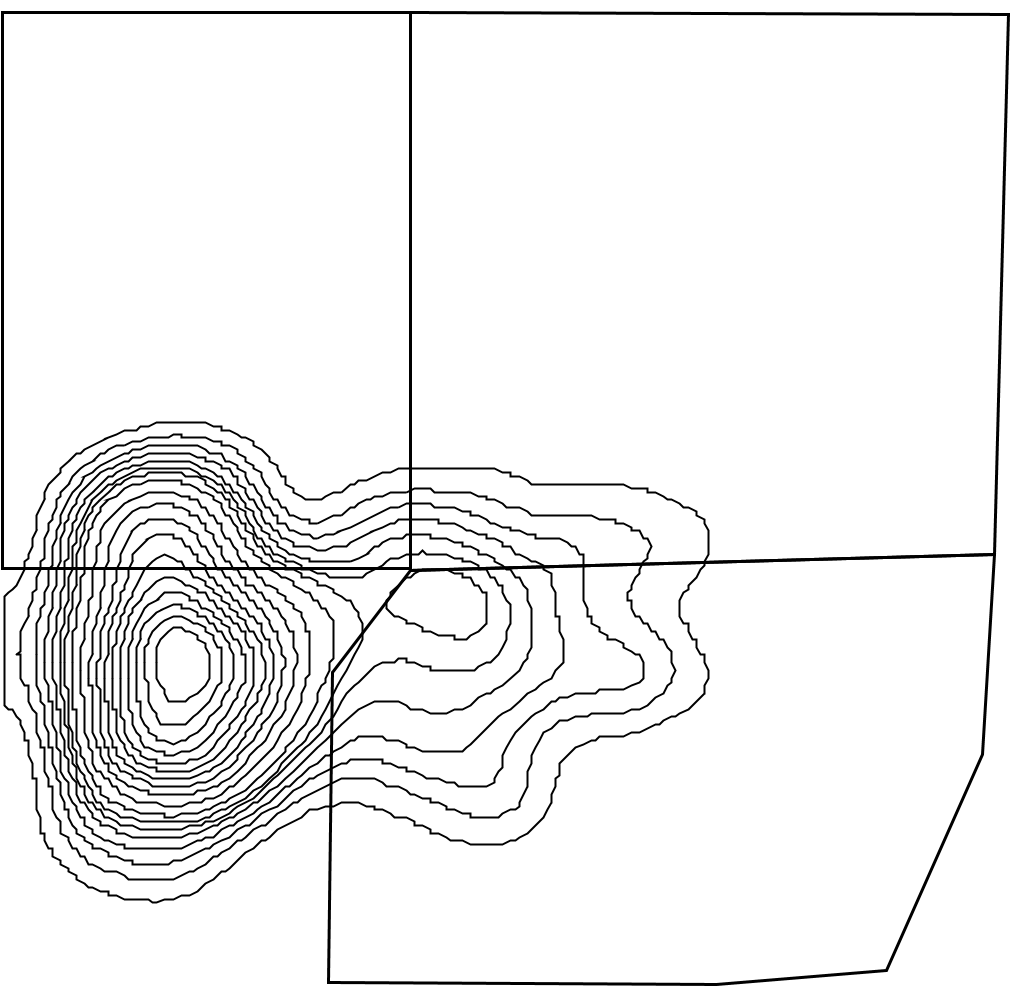

Supplement: Supplementary file 13 — Source Data for Figure 7 [file EMMM-15-e17694-s004.zip › Figure 7/7F/7F GMP base.tiff]

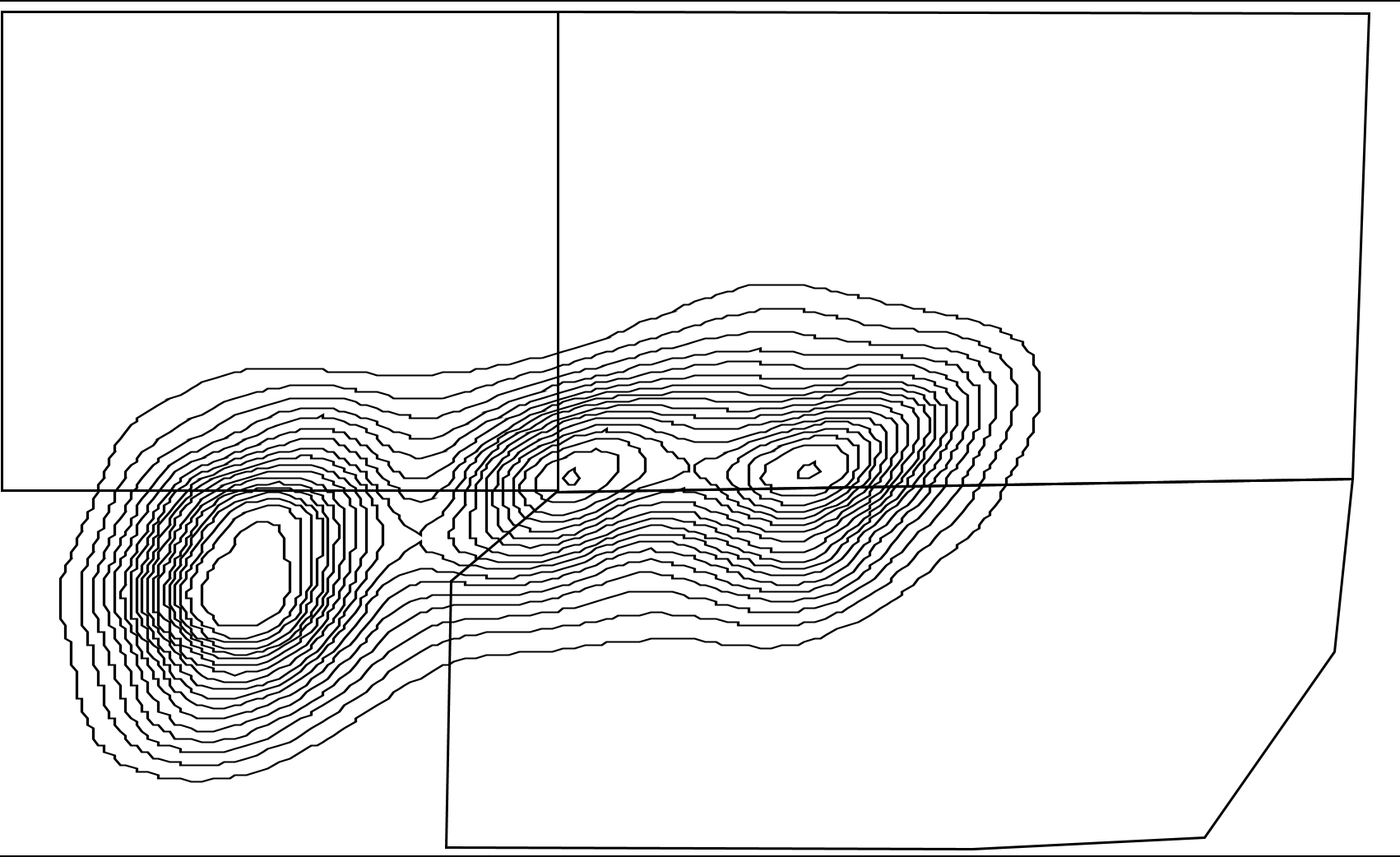

Supplement: Supplementary file 13 — Source Data for Figure 7 [file EMMM-15-e17694-s004.zip › Figure 7/7F/7F GMP M d+5.tiff]

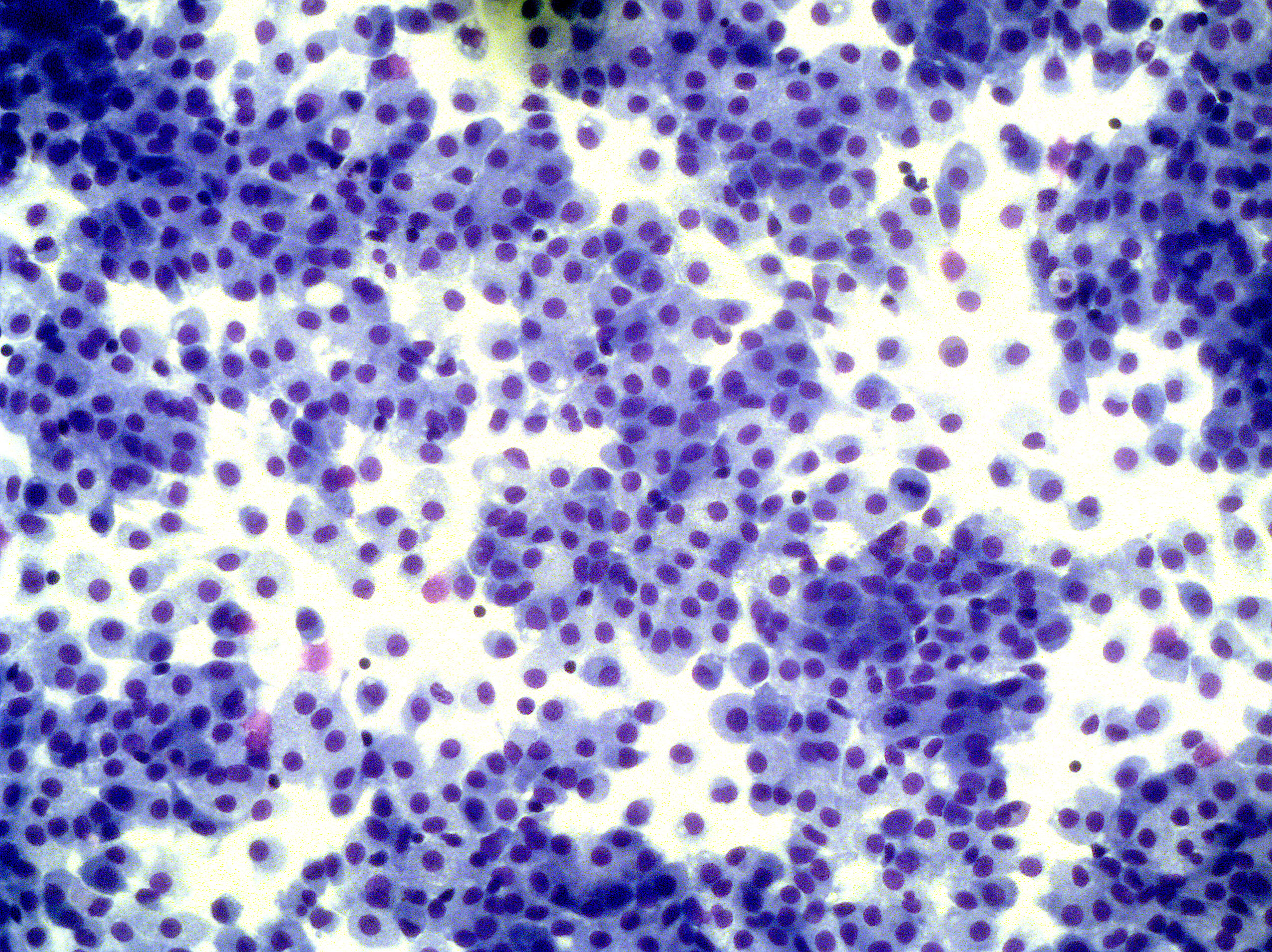

Supplement: Supplementary file 13 — Source Data for Figure 7 [file EMMM-15-e17694-s004.zip › Figure 7/7A/MSJSNK d+5 M.tiff]

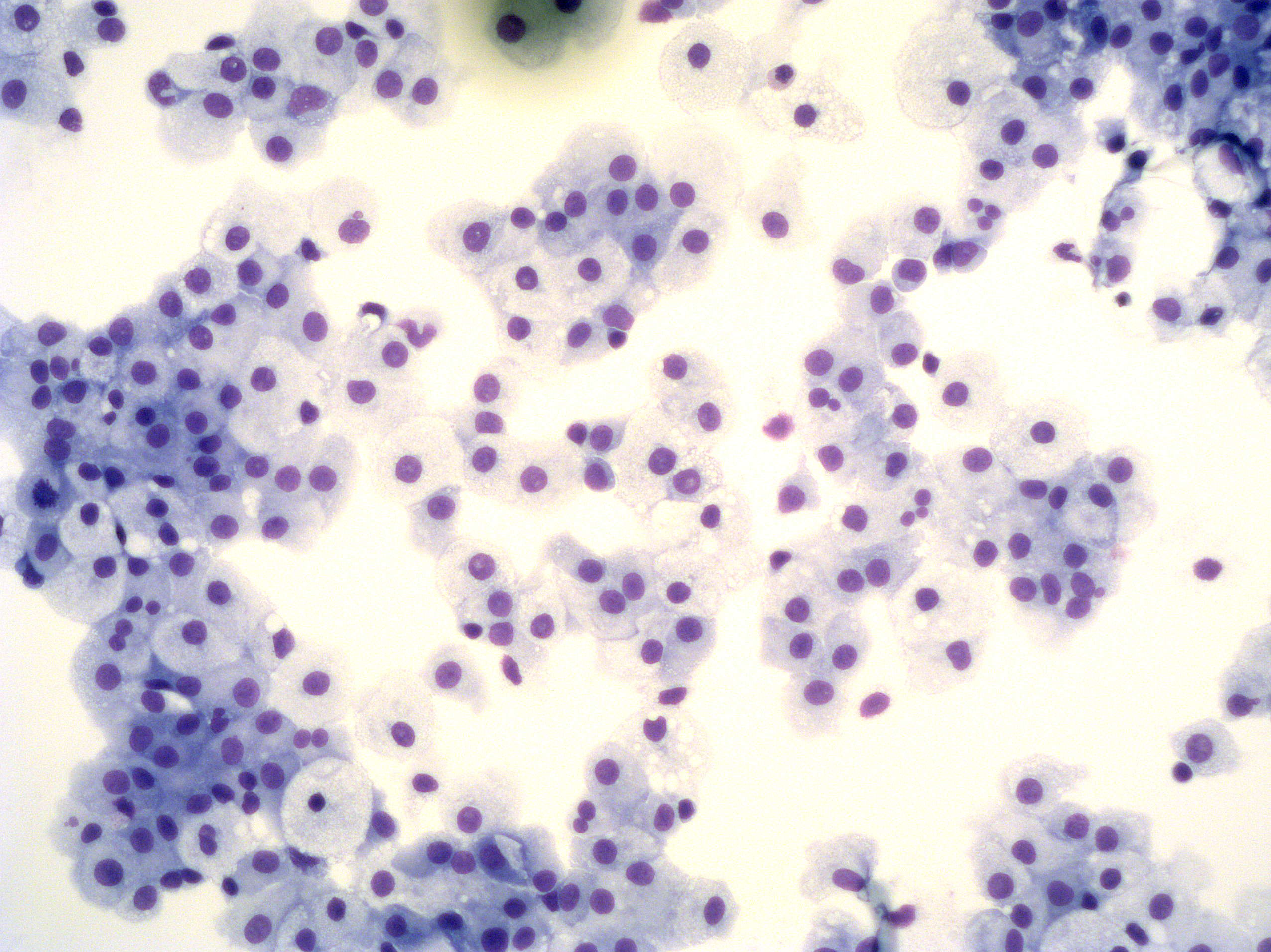

Supplement: Supplementary file 13 — Source Data for Figure 7 [file EMMM-15-e17694-s004.zip › Figure 7/7A/MSJSNK d+9 M.tiff]

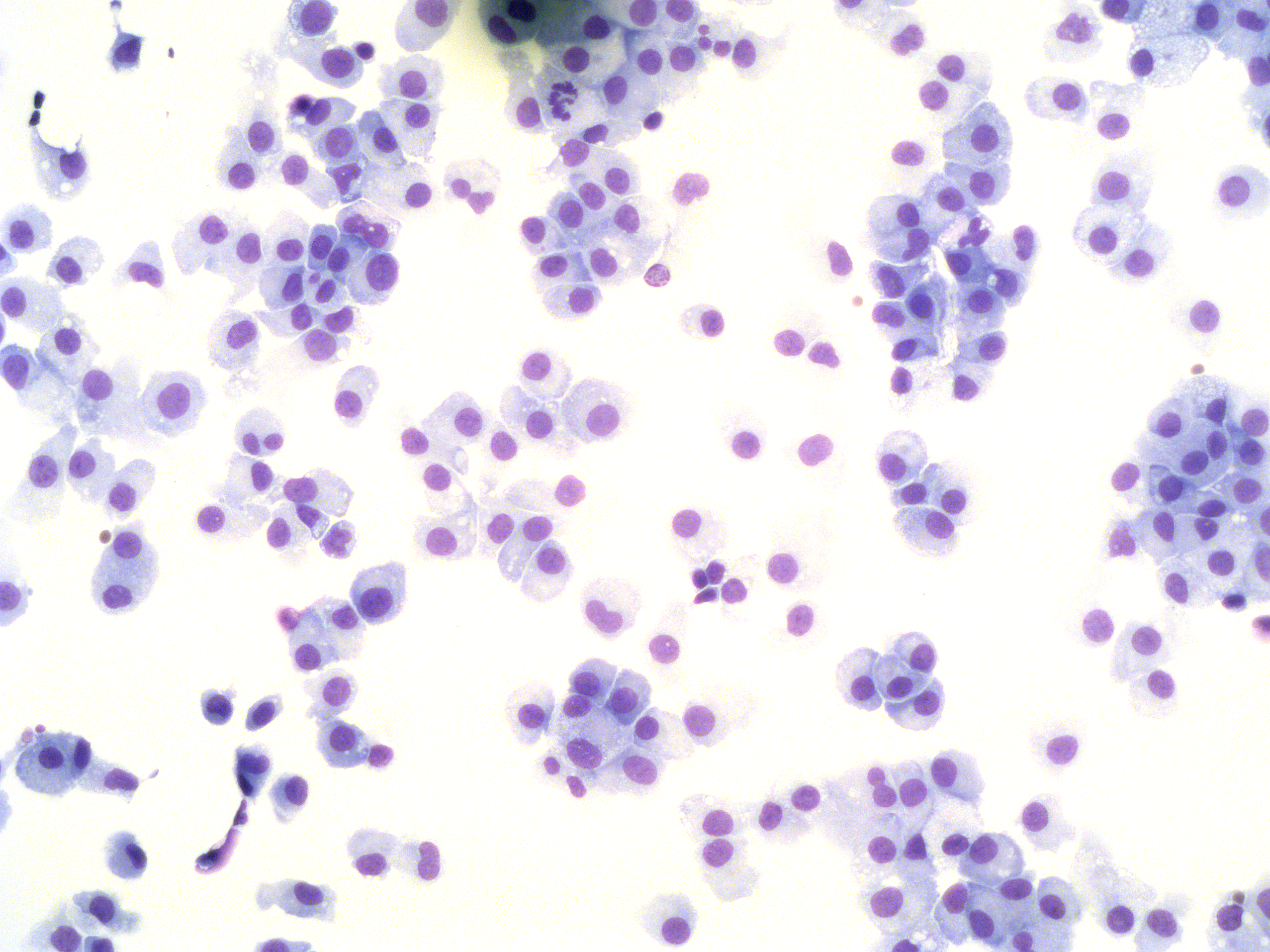

Supplement: Supplementary file 13 — Source Data for Figure 7 [file EMMM-15-e17694-s004.zip › Figure 7/7A/MSJSNK d+9 IL3.tiff]

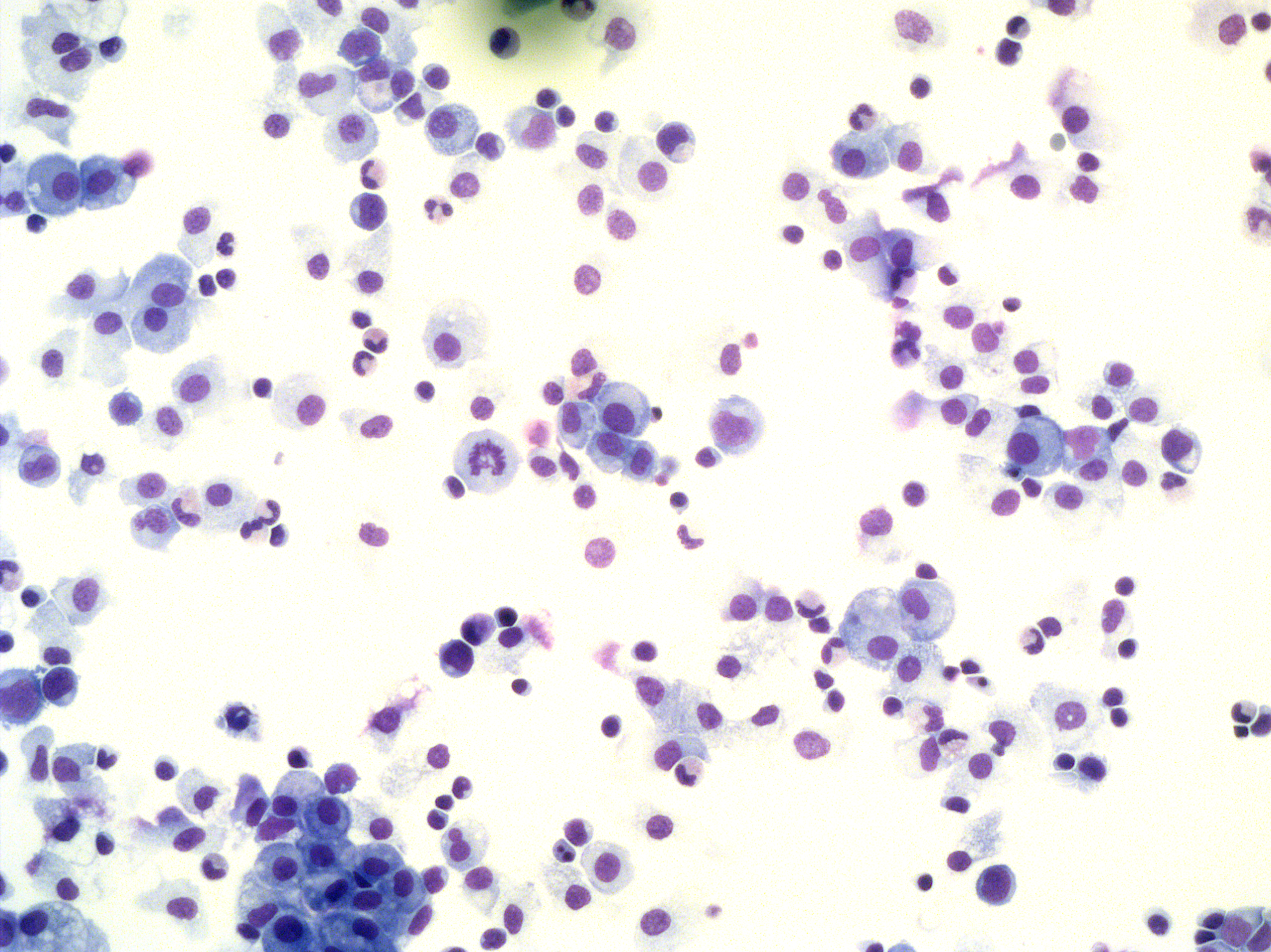

Supplement: Supplementary file 13 — Source Data for Figure 7 [file EMMM-15-e17694-s004.zip › Figure 7/7A/MSJSNK SCFd+5.tiff]

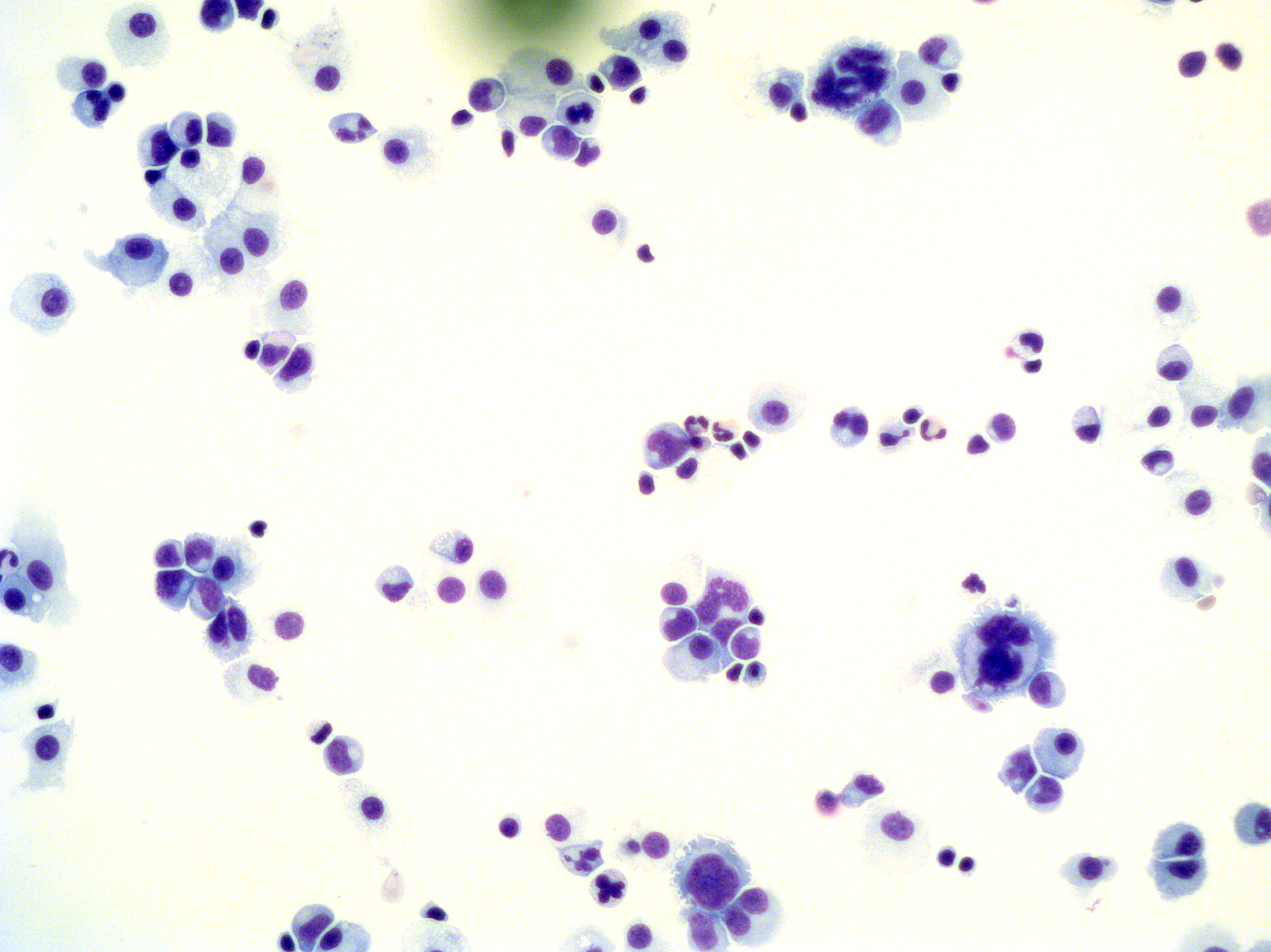

Supplement: Supplementary file 13 — Source Data for Figure 7 [file EMMM-15-e17694-s004.zip › Figure 7/7A/MSJSNK SCF d+9.tiff]

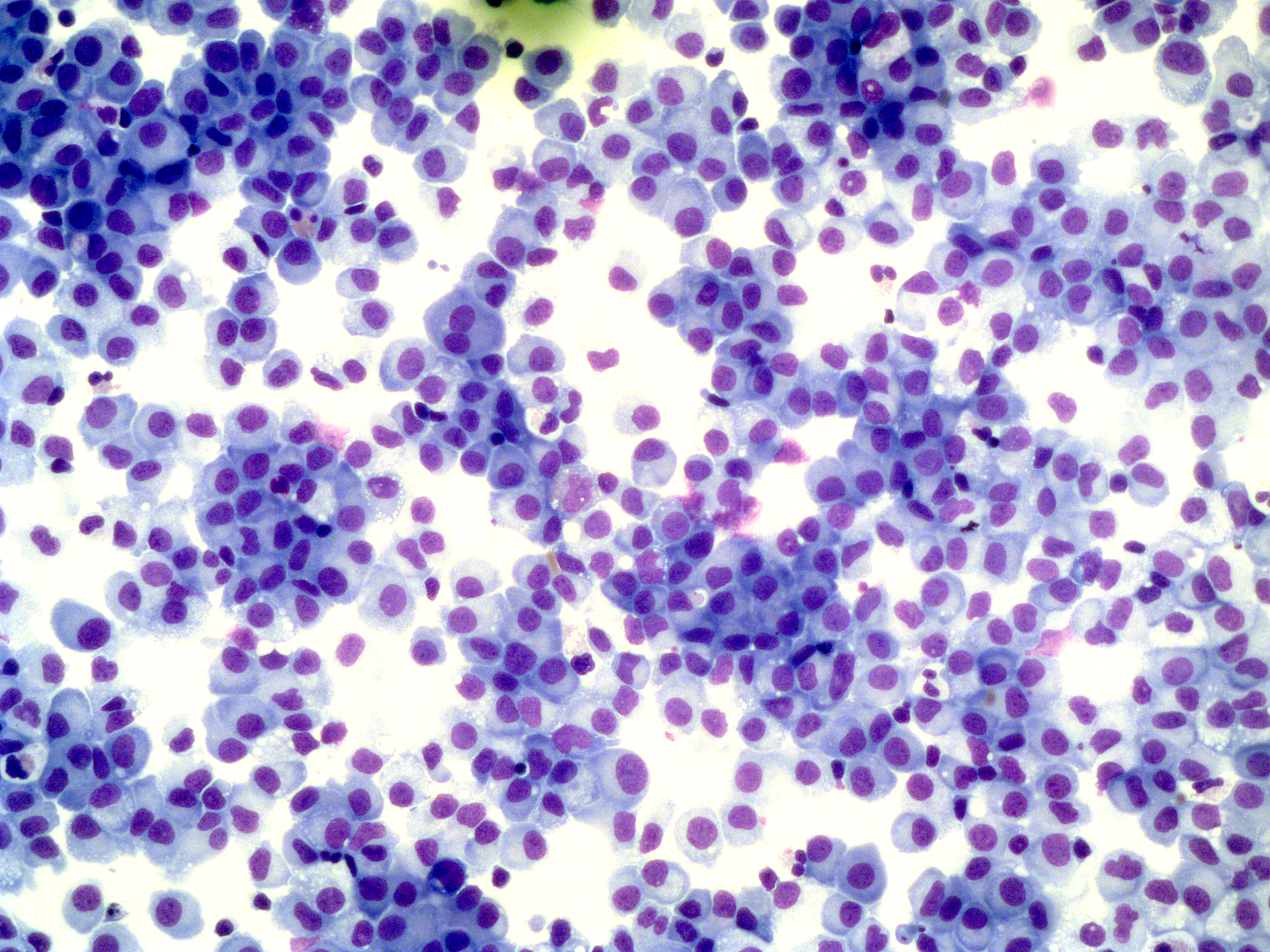

Supplement: Supplementary file 13 — Source Data for Figure 7 [file EMMM-15-e17694-s004.zip › Figure 7/7A/MSJSNK d+5 IL3.tiff]

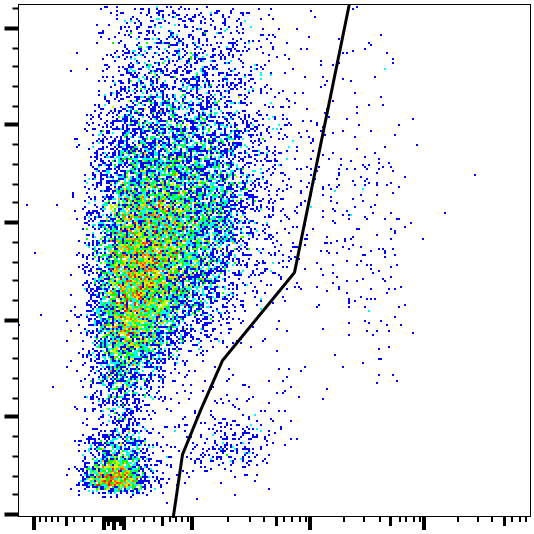

Supplement: Supplementary file 13 — Source Data for Figure 7 [file EMMM-15-e17694-s004.zip › Figure 7/7C/CD34/CD34 M.tiff]

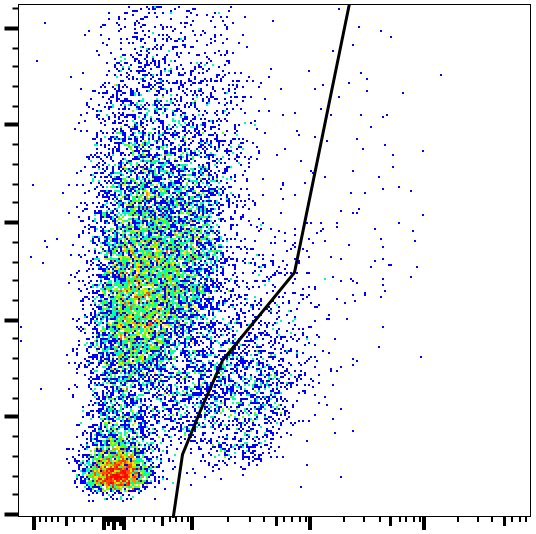

Supplement: Supplementary file 13 — Source Data for Figure 7 [file EMMM-15-e17694-s004.zip › Figure 7/7C/CD34/CD34 IL3.tiff]

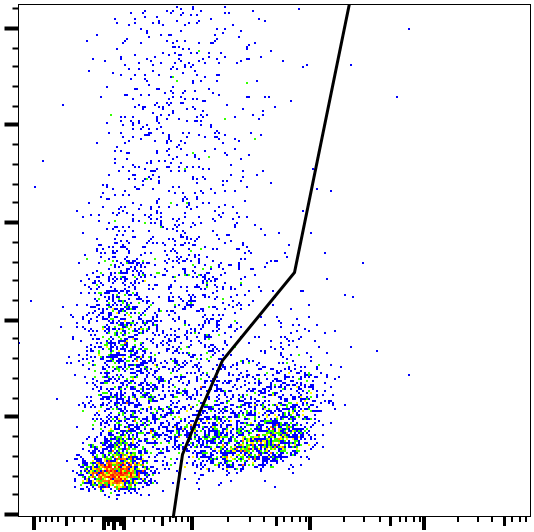

Supplement: Supplementary file 13 — Source Data for Figure 7 [file EMMM-15-e17694-s004.zip › Figure 7/7C/CD34/CD34 SCF.tiff]

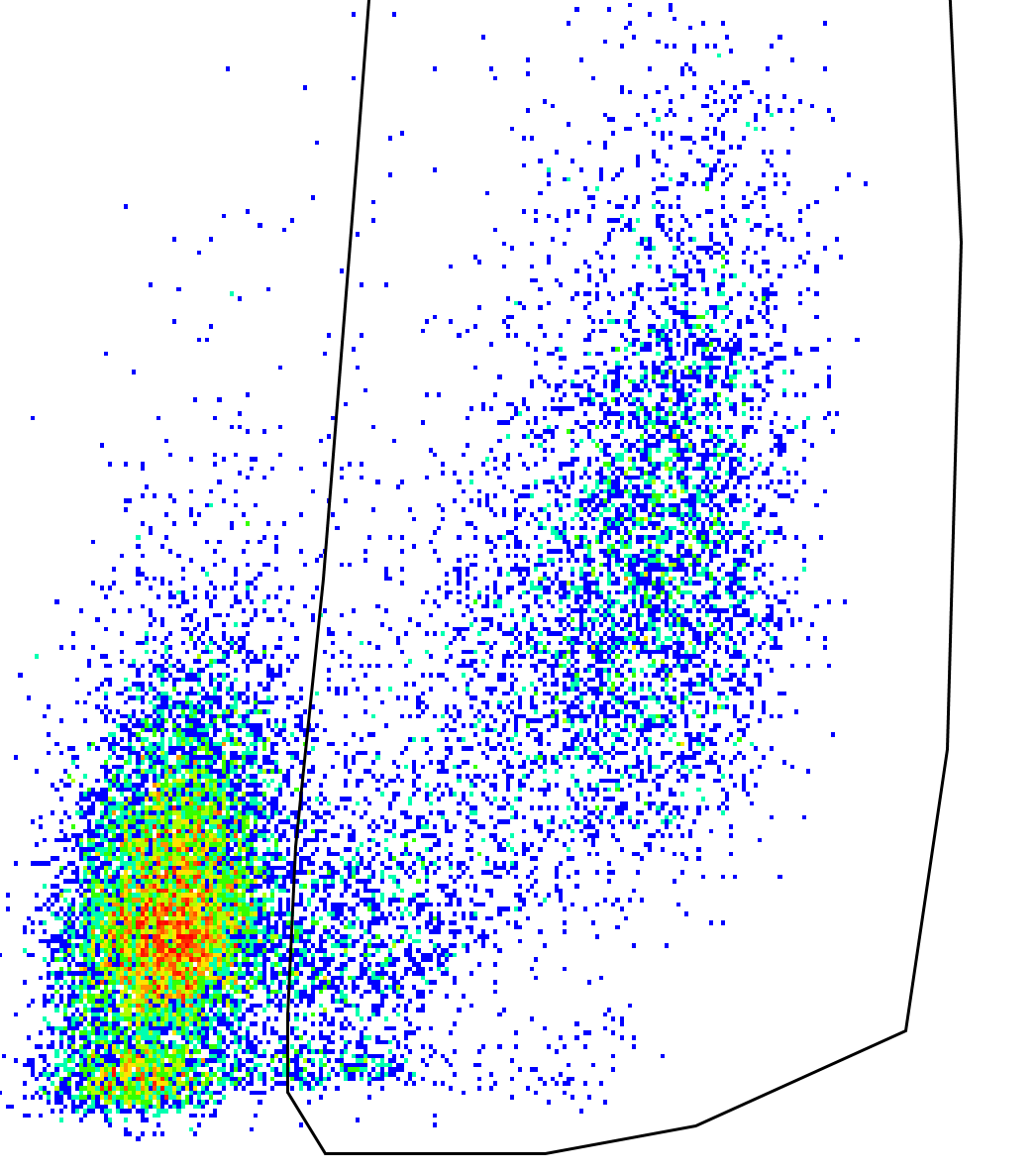

Supplement: Supplementary file 13 — Source Data for Figure 7 [file EMMM-15-e17694-s004.zip › Figure 7/7C/CD11b/CD11b IL3.tiff]

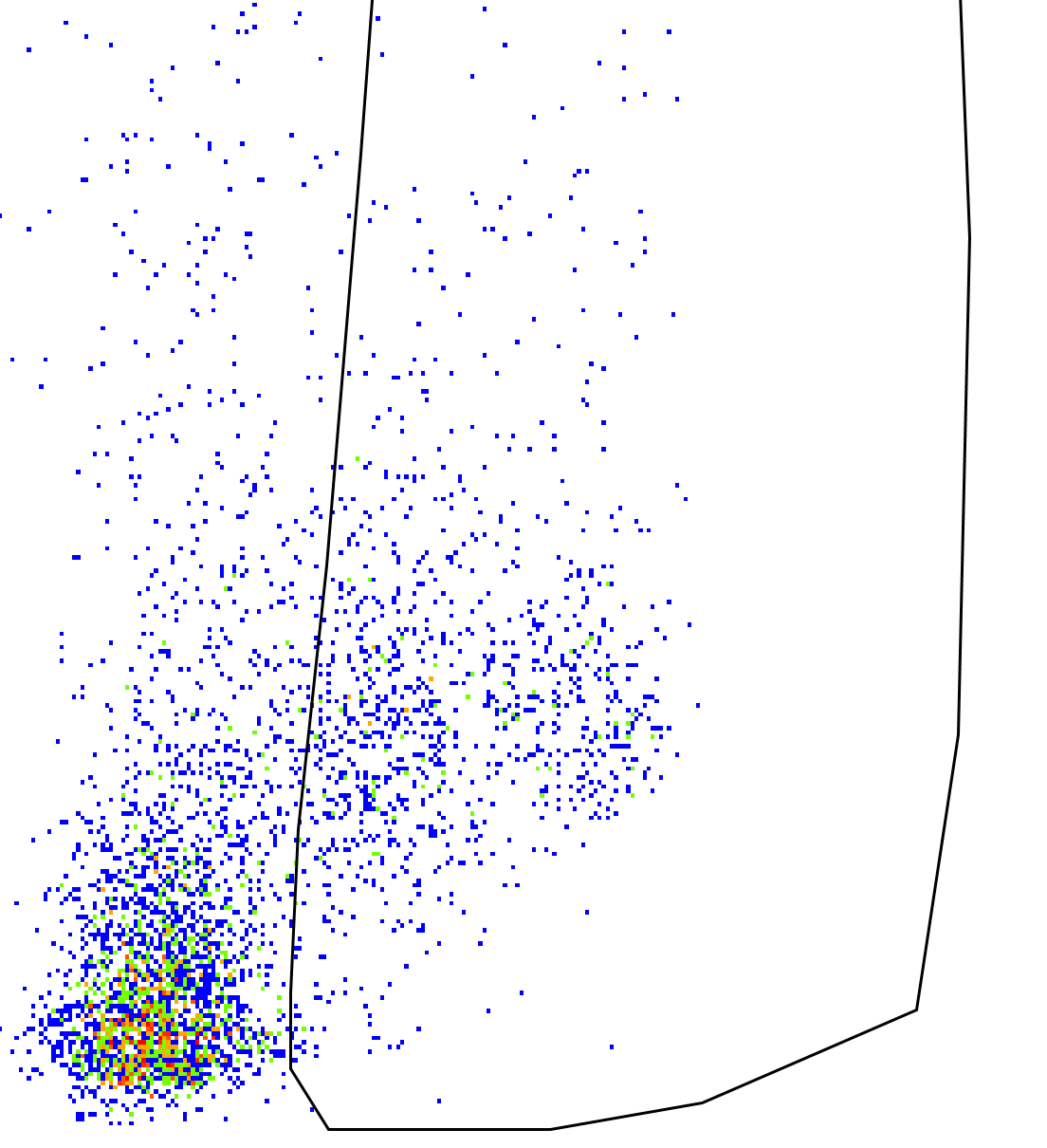

Supplement: Supplementary file 13 — Source Data for Figure 7 [file EMMM-15-e17694-s004.zip › Figure 7/7C/CD11b/CD11b SCF.tiff]

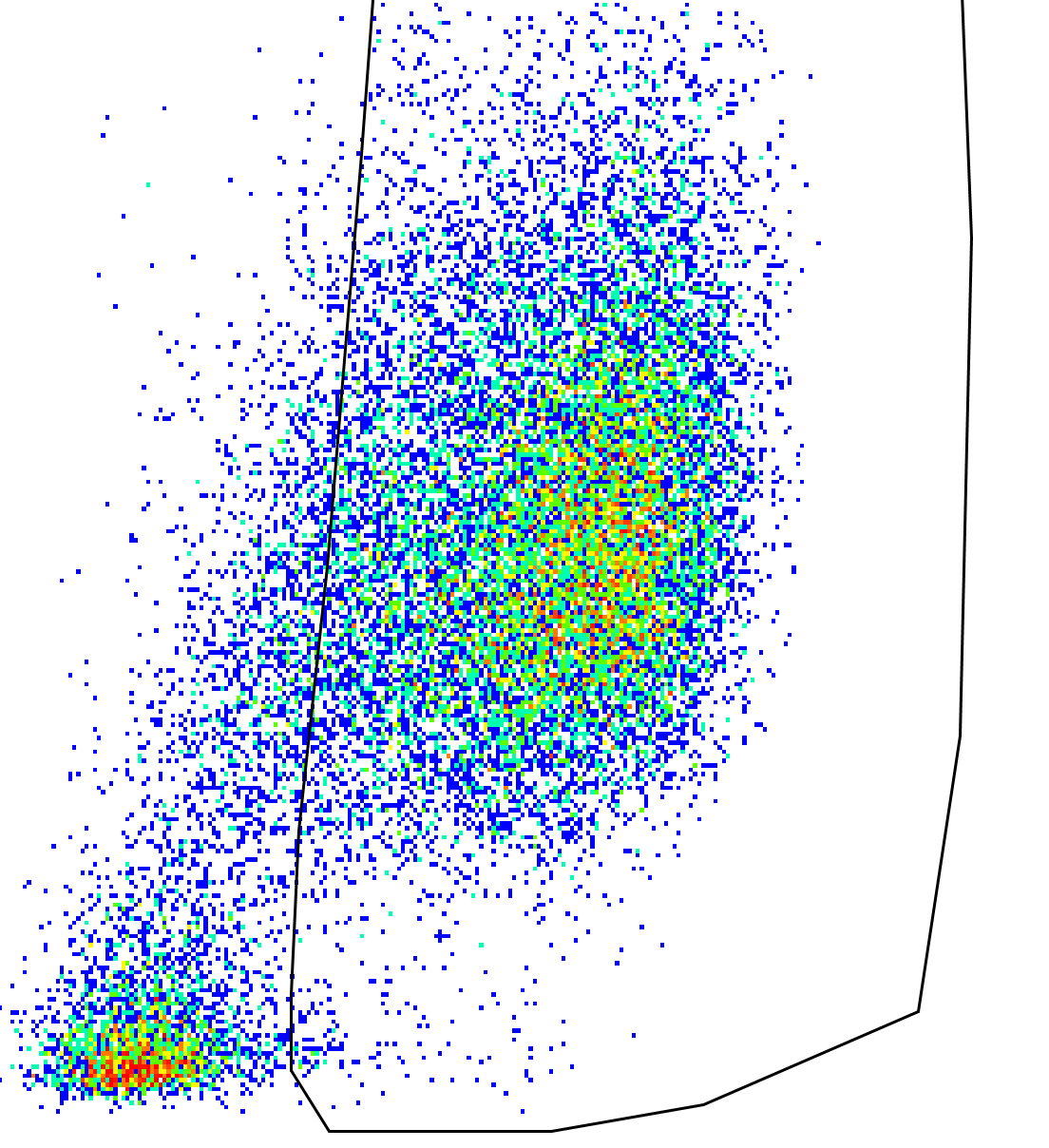

Supplement: Supplementary file 13 — Source Data for Figure 7 [file EMMM-15-e17694-s004.zip › Figure 7/7C/CD11b/CD11b M-CSF.tiff]

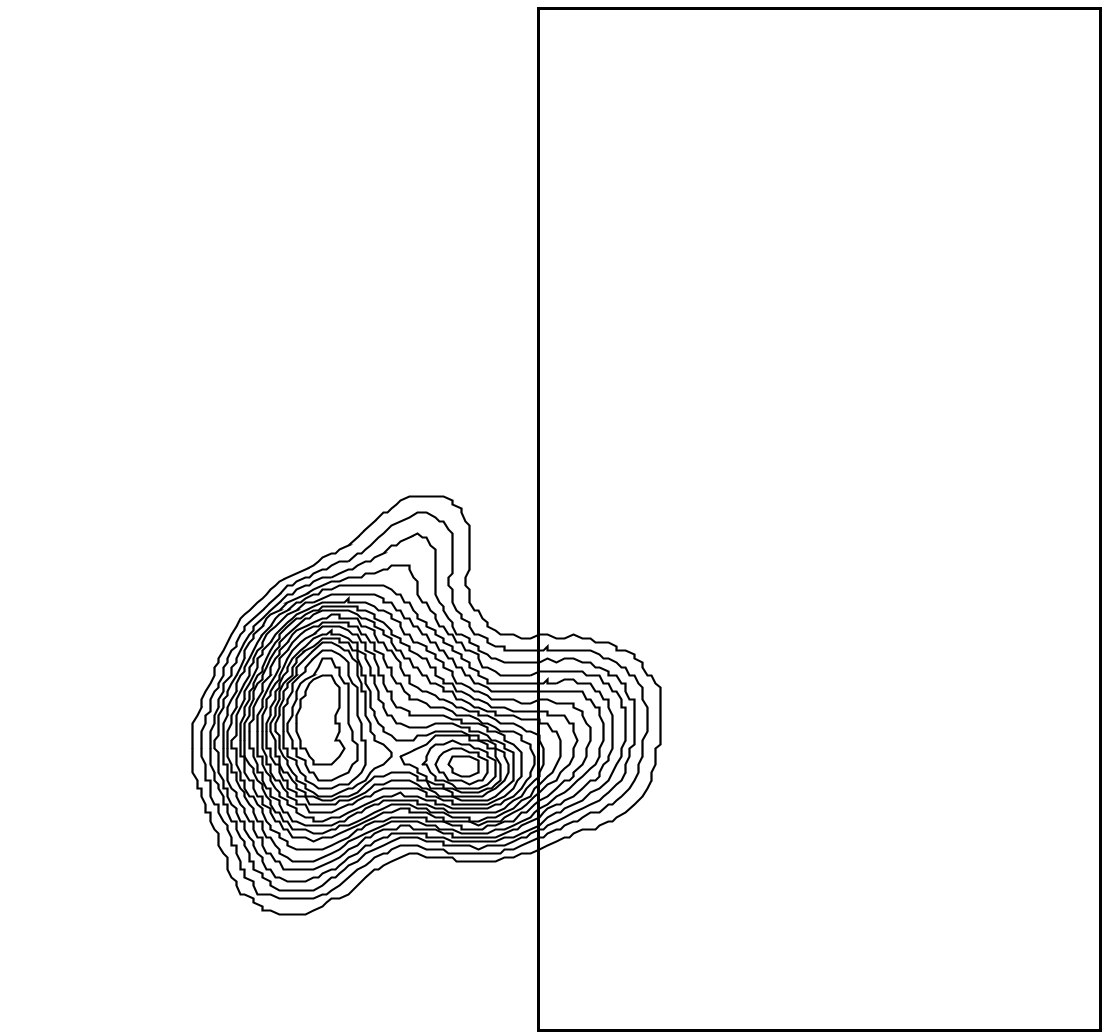

Supplement: Supplementary file 14 — Source Data for Figure 8 [file EMMM-15-e17694-s012.zip › Figure 8/8F/GrB IL-3.tiff]

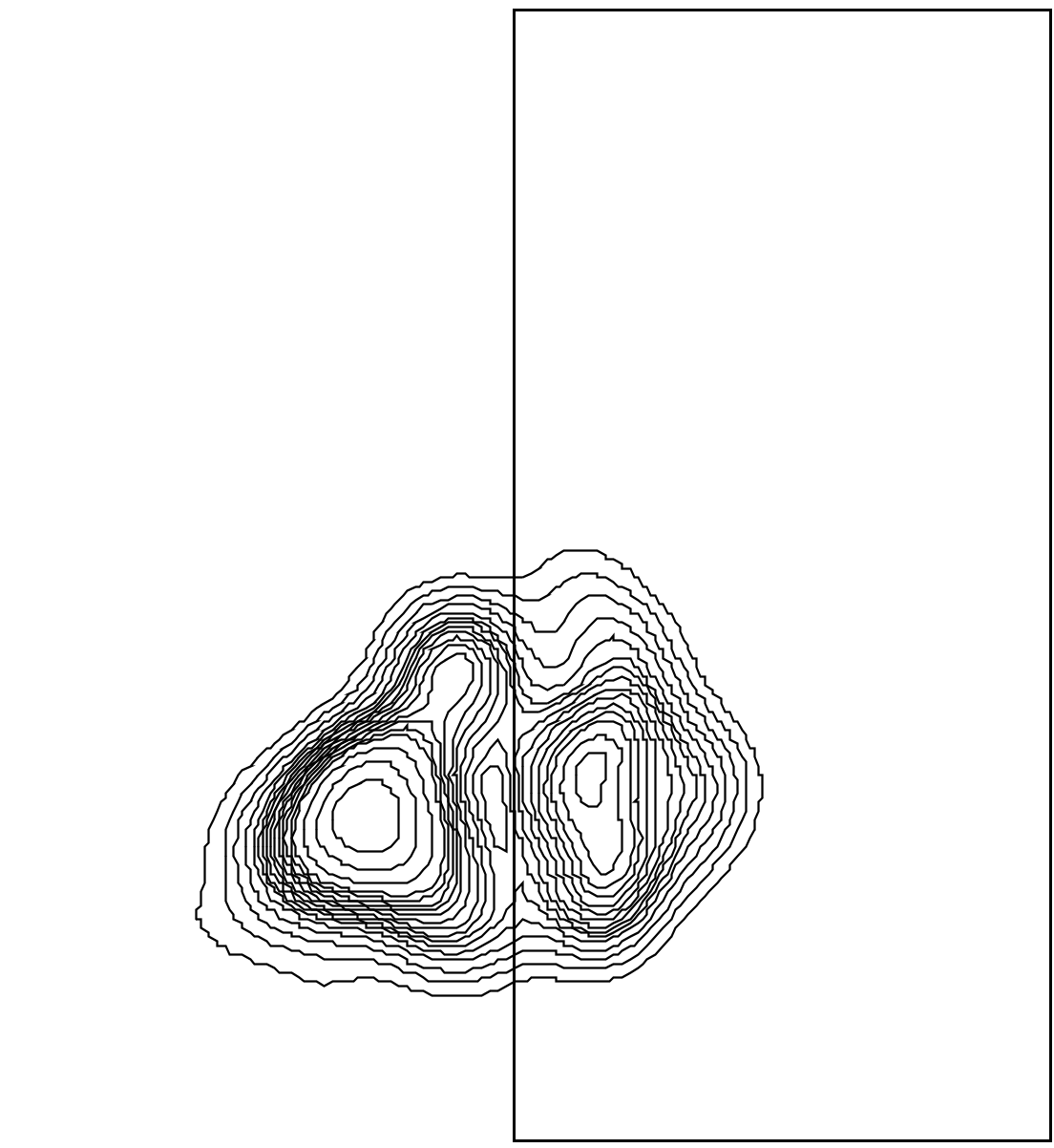

Supplement: Supplementary file 14 — Source Data for Figure 8 [file EMMM-15-e17694-s012.zip › Figure 8/8F/GrB M-CSF.tiff]

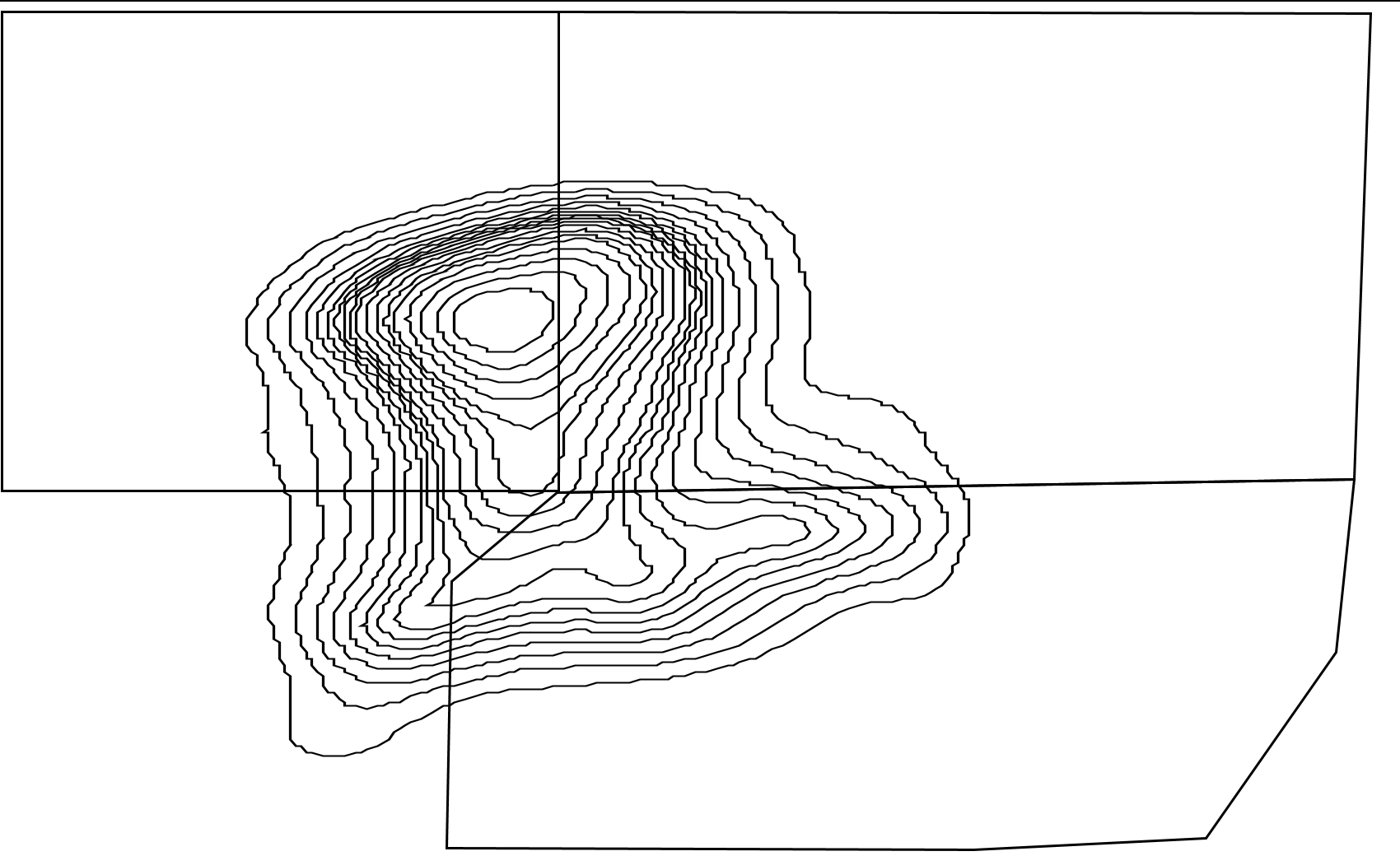

Supplement: Supplementary file 14 — Source Data for Figure 8 [file EMMM-15-e17694-s012.zip › Figure 8/8D/CLIPs IL3.tiff]

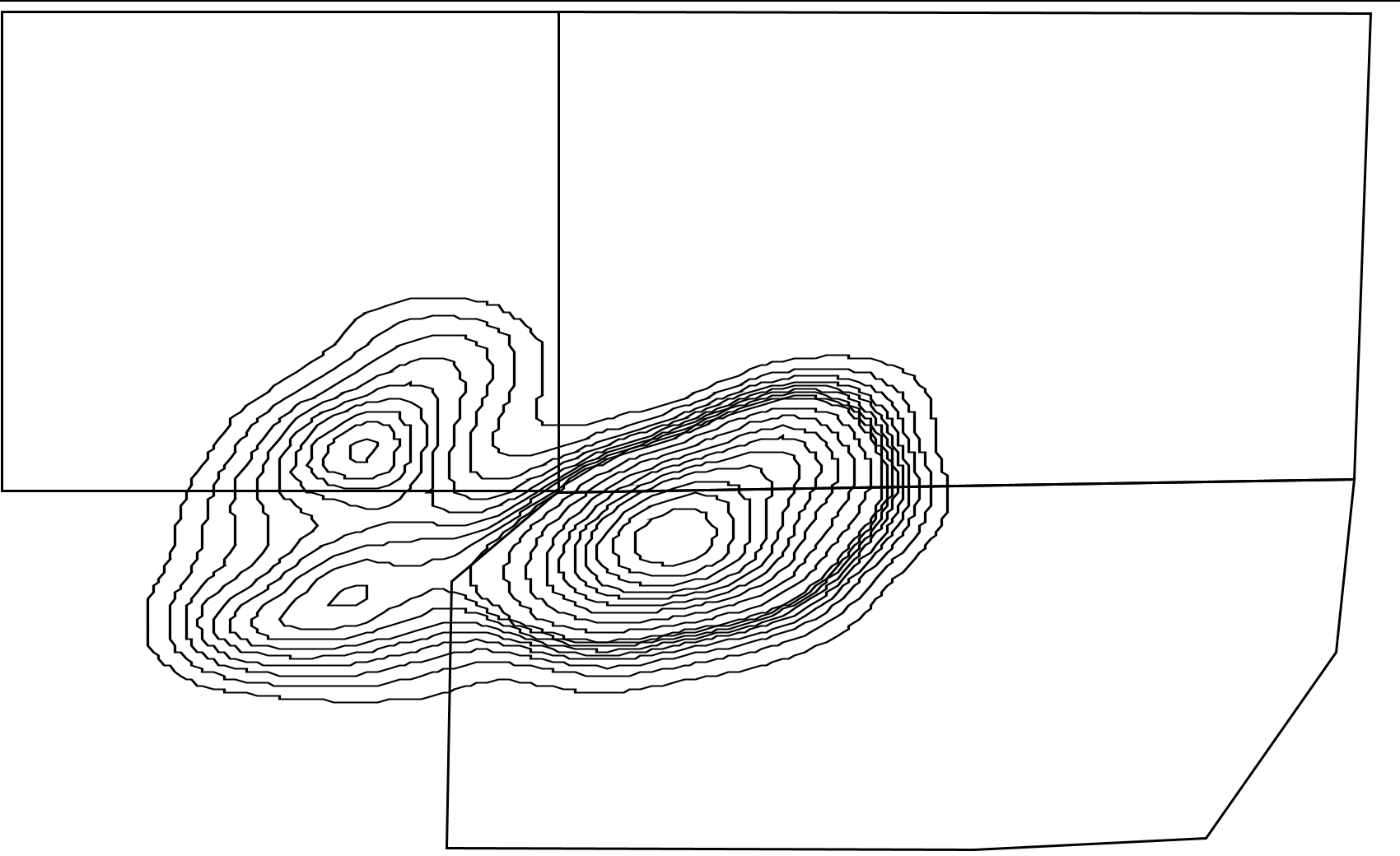

Supplement: Supplementary file 14 — Source Data for Figure 8 [file EMMM-15-e17694-s012.zip › Figure 8/8D/CLIPs M-CSF.tiff]

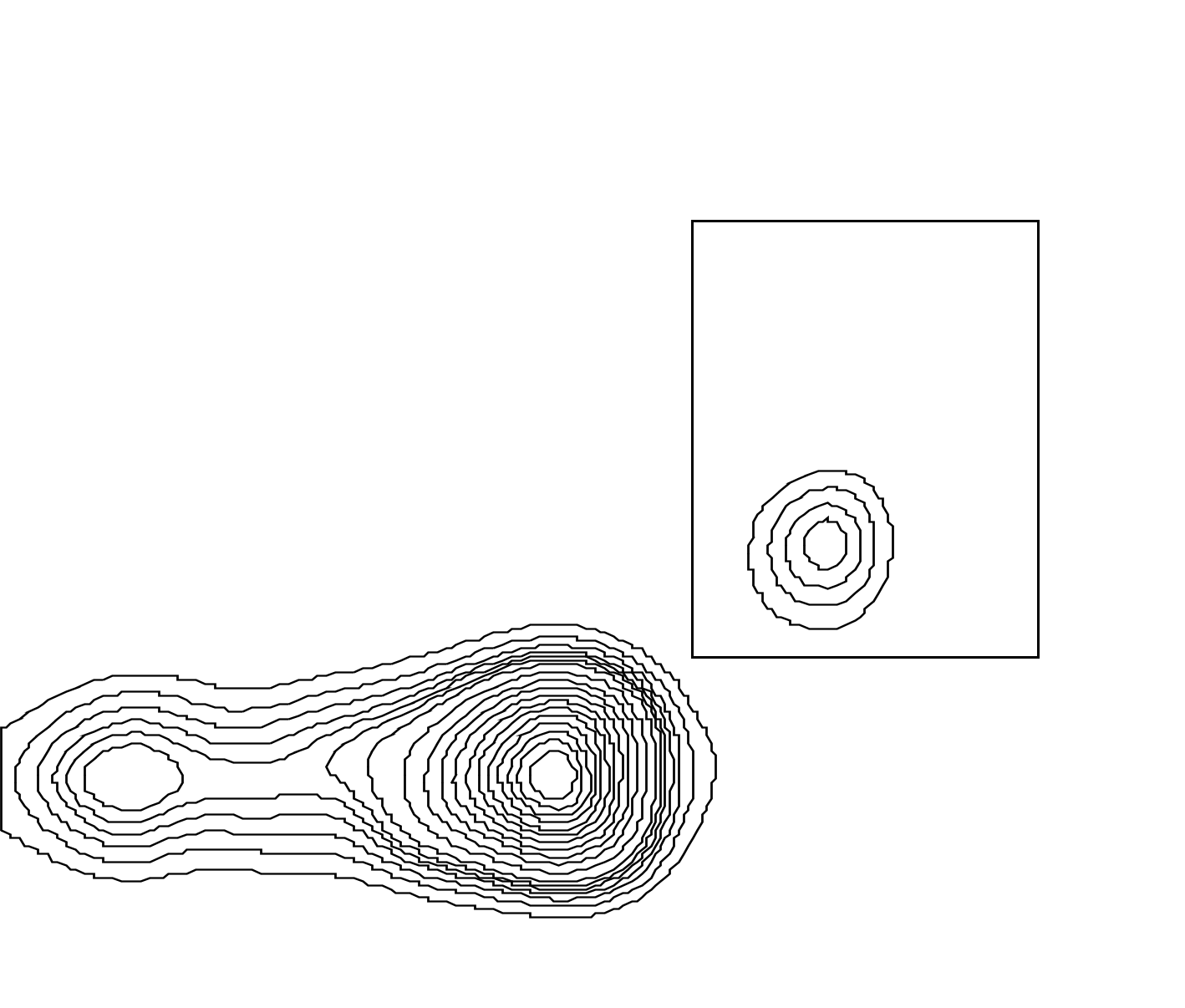

Supplement: Supplementary file 14 — Source Data for Figure 8 [file EMMM-15-e17694-s012.zip › Figure 8/8E/NKs IL-3.tiff]

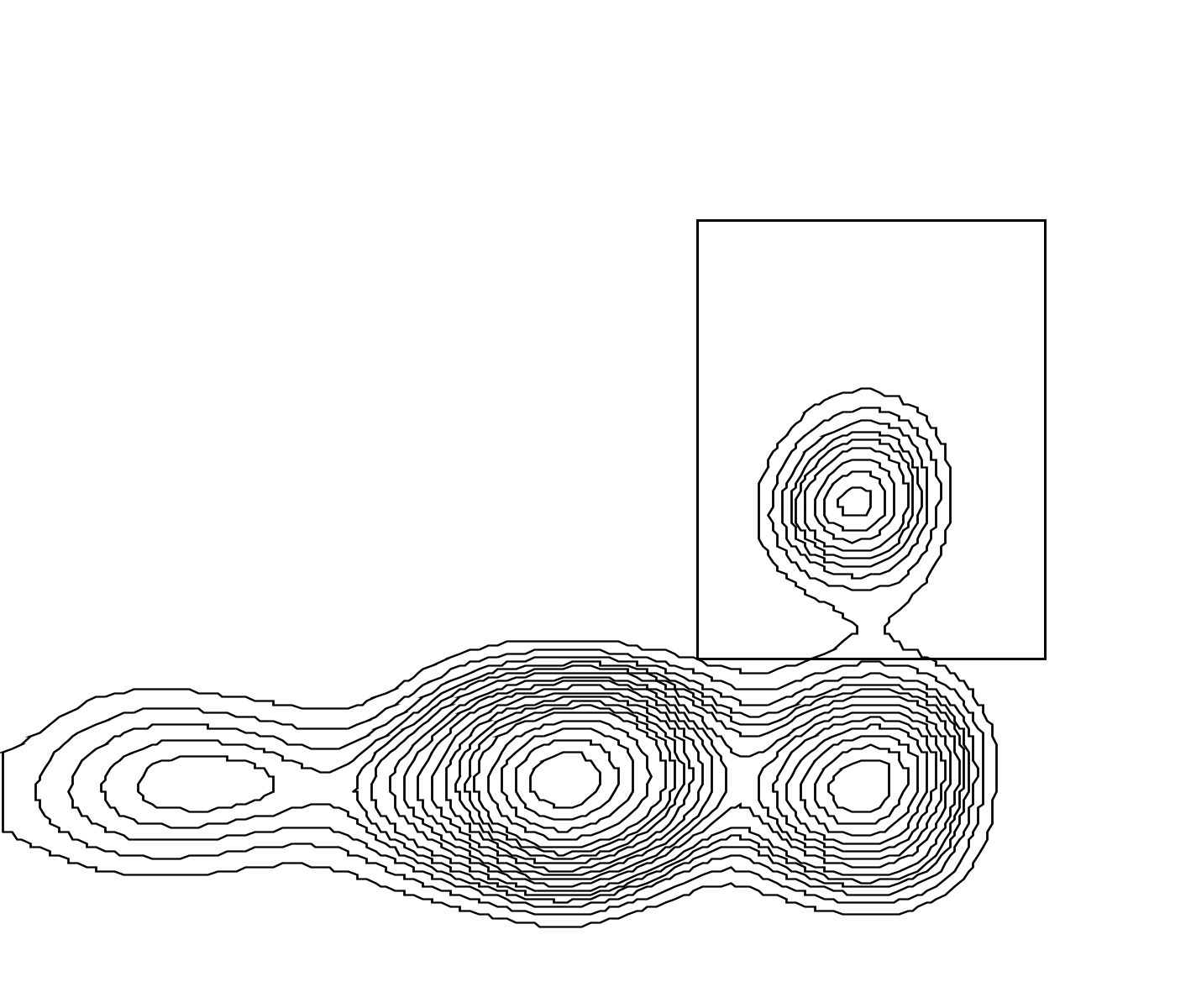

Supplement: Supplementary file 14 — Source Data for Figure 8 [file EMMM-15-e17694-s012.zip › Figure 8/8E/NKs M-CSF.tiff]

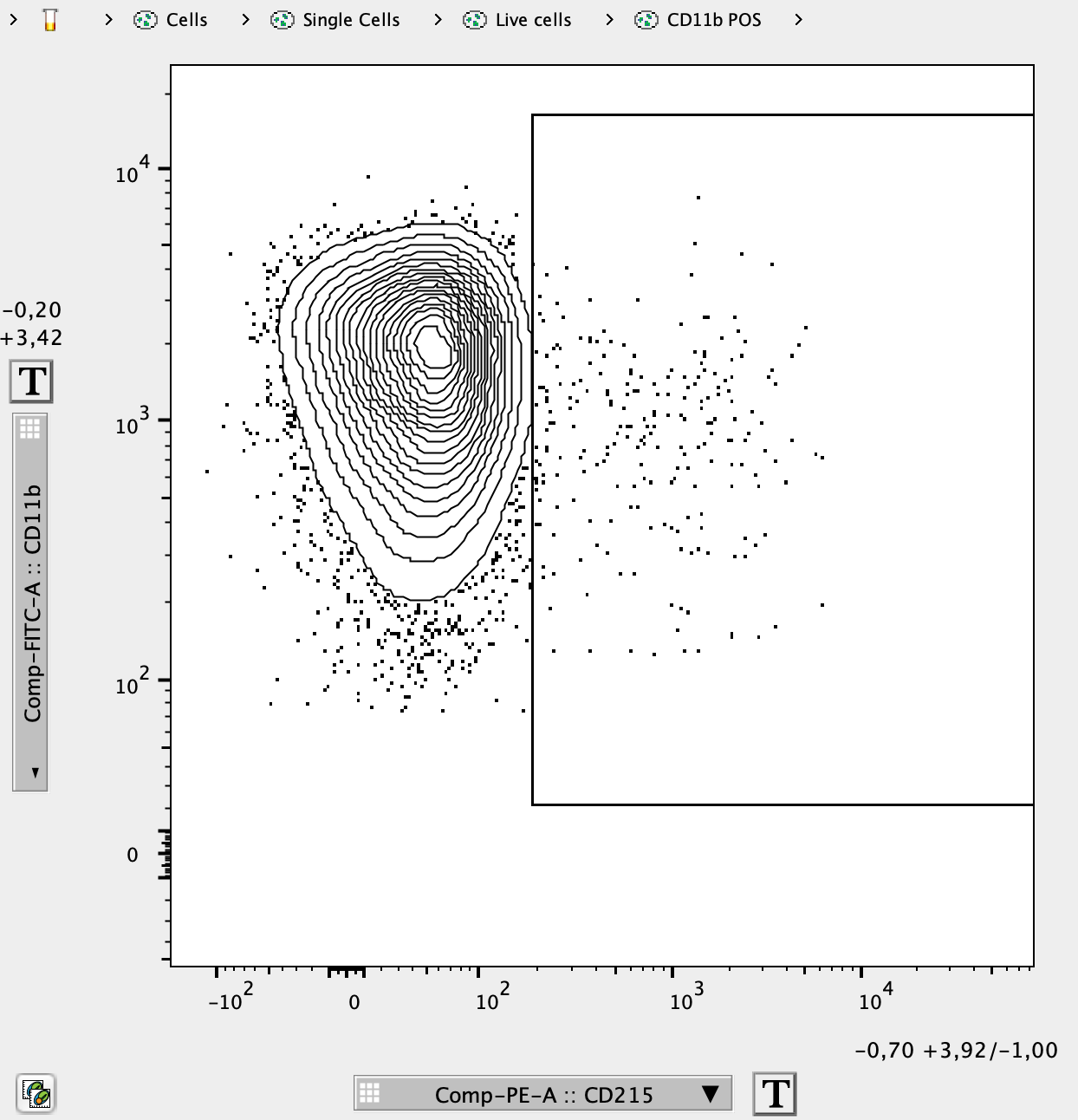

Supplement: Supplementary file 14 — Source Data for Figure 8 [file EMMM-15-e17694-s012.zip › Figure 8/8A/CD11b IL15Ra+/8A IL-3 d+9.tiff]

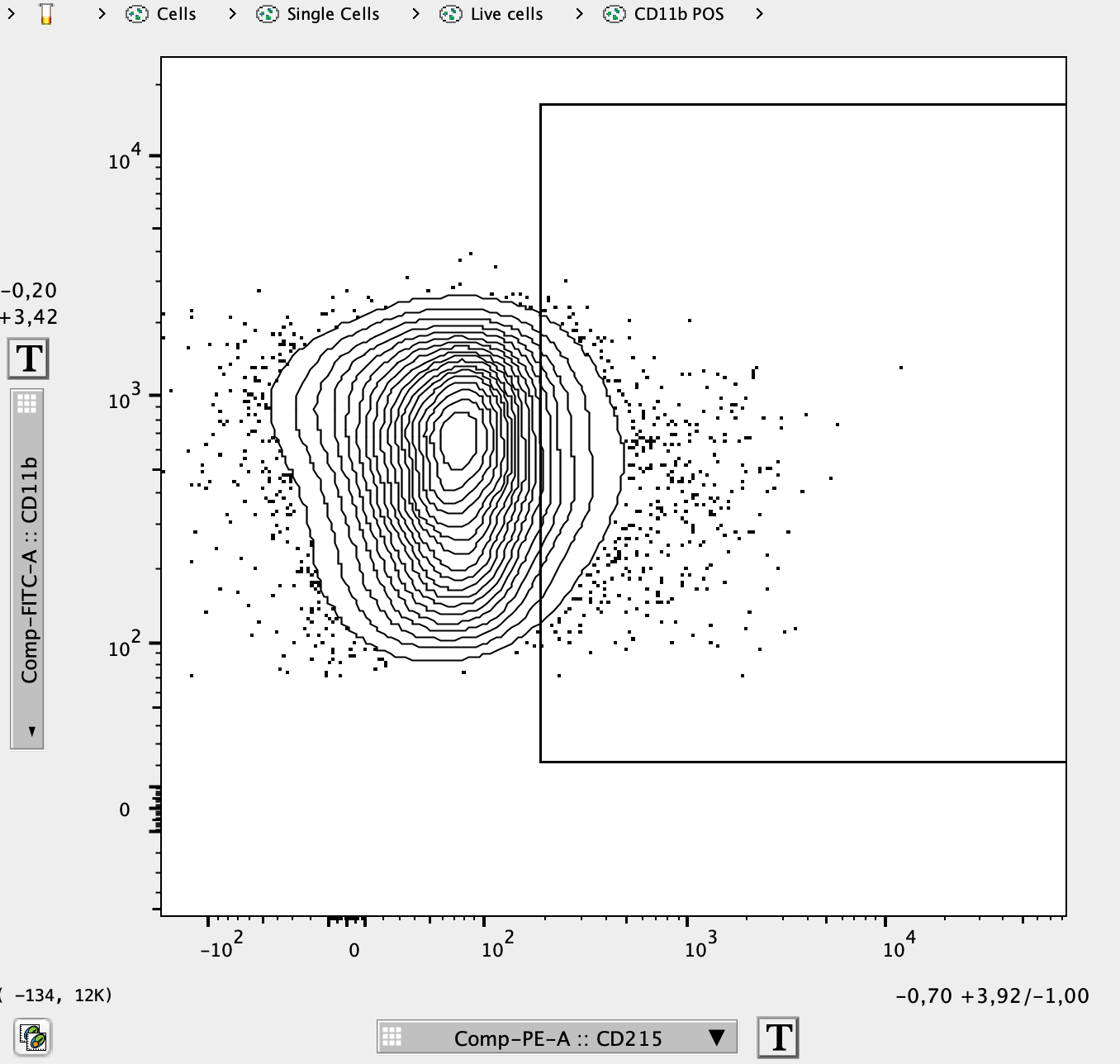

Supplement: Supplementary file 14 — Source Data for Figure 8 [file EMMM-15-e17694-s012.zip › Figure 8/8A/CD11b IL15Ra+/8A M-CSF d+9.tiff]

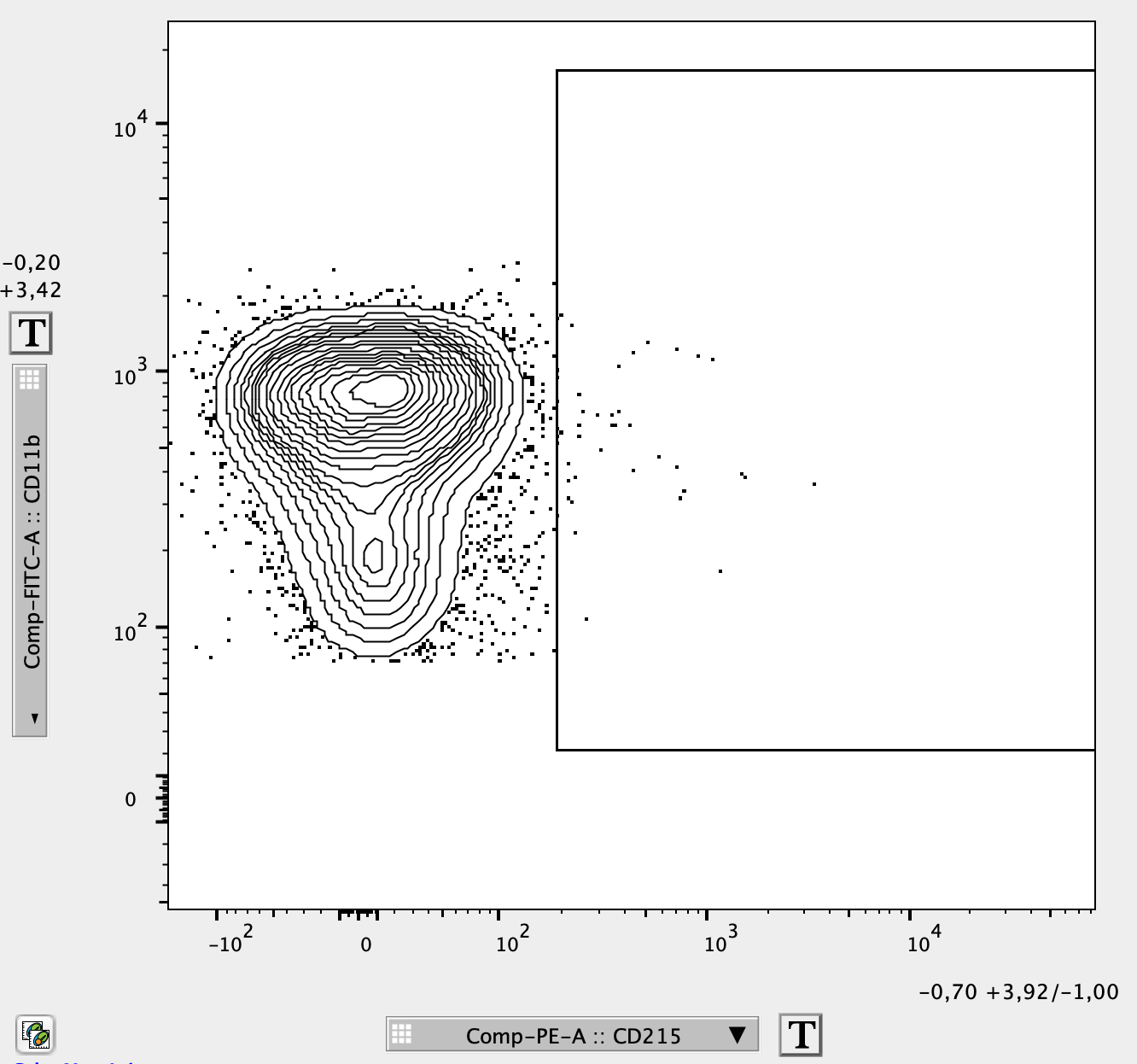

Supplement: Supplementary file 14 — Source Data for Figure 8 [file EMMM-15-e17694-s012.zip › Figure 8/8A/CD11b IL15Ra+/8A Base.tiff]

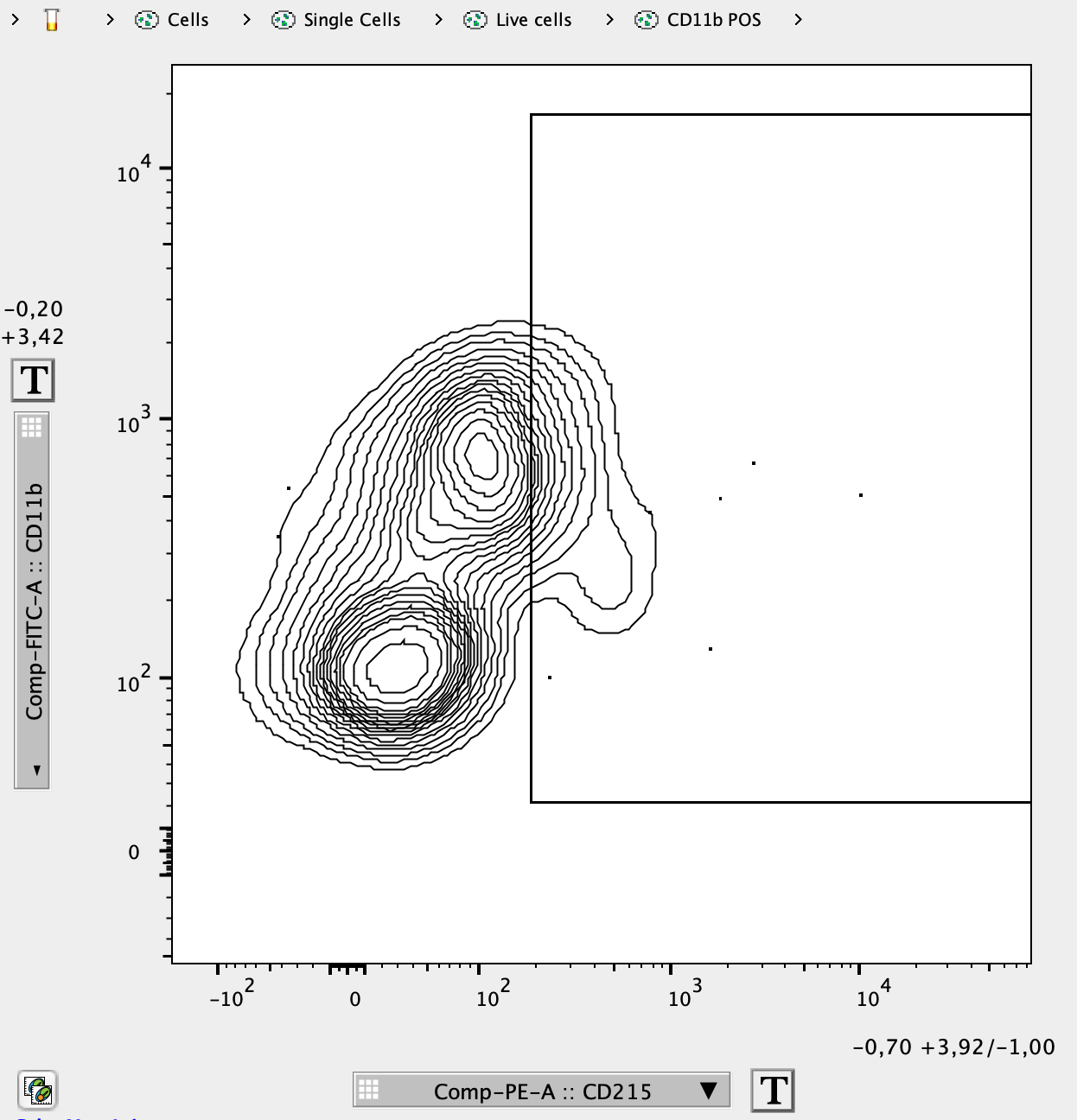

Supplement: Supplementary file 14 — Source Data for Figure 8 [file EMMM-15-e17694-s012.zip › Figure 8/8A/CD11b IL15Ra+/8A SCF d+9.tiff]

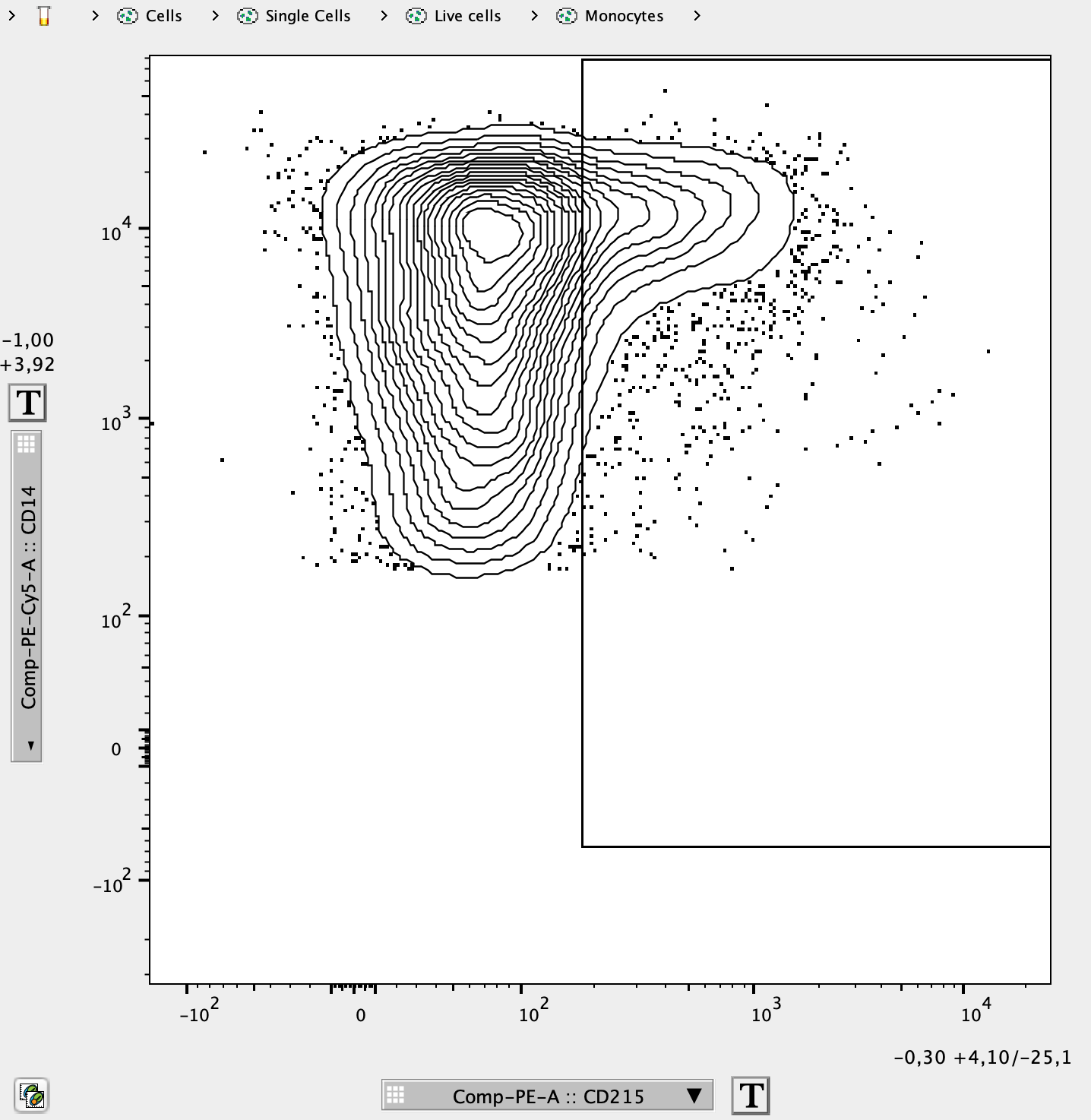

Supplement: Supplementary file 14 — Source Data for Figure 8 [file EMMM-15-e17694-s012.zip › Figure 8/8A/CD14 IL15Ra+/8A M-CSF d+9.tiff]

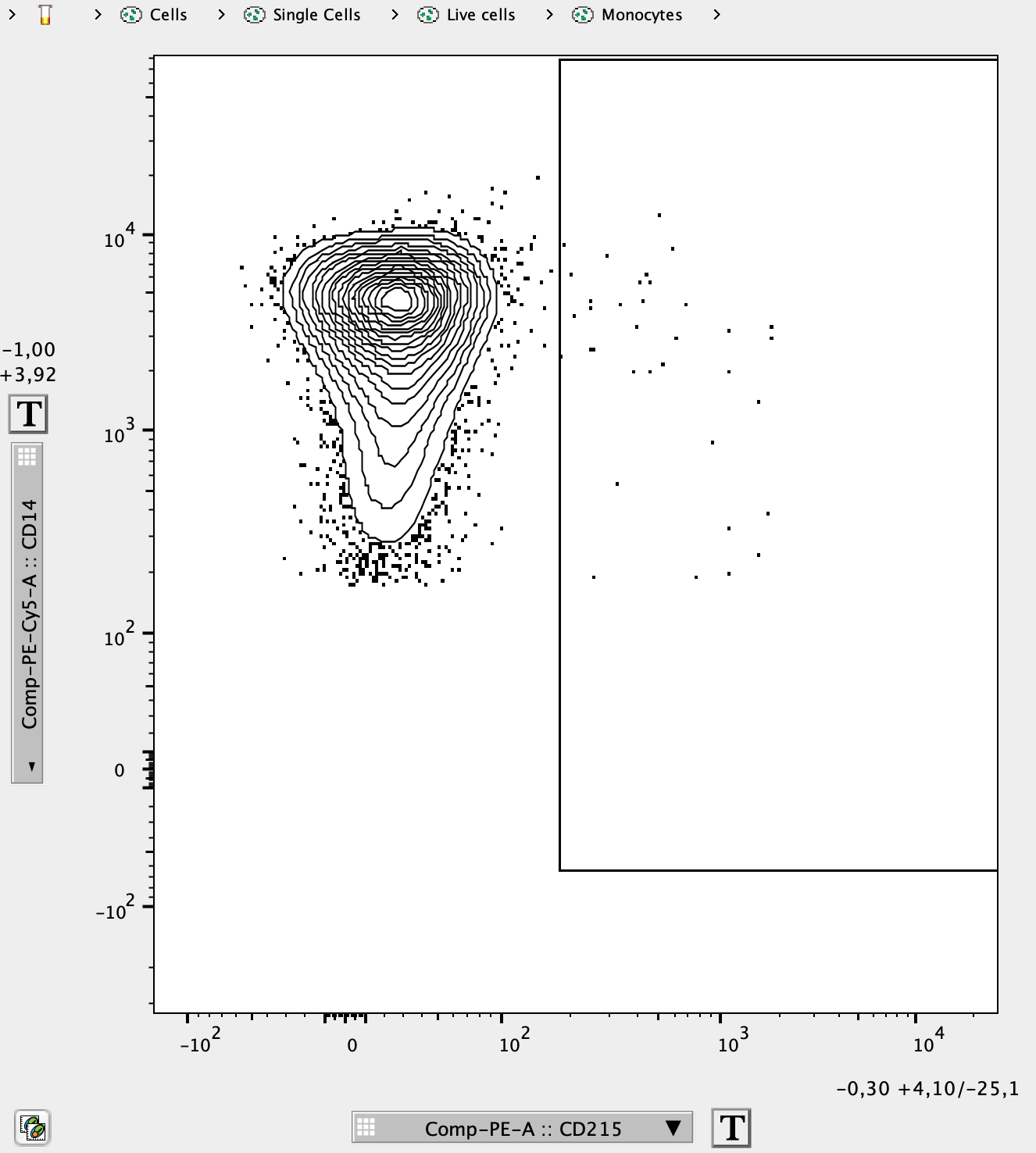

Supplement: Supplementary file 14 — Source Data for Figure 8 [file EMMM-15-e17694-s012.zip › Figure 8/8A/CD14 IL15Ra+/8A Base.tiff]

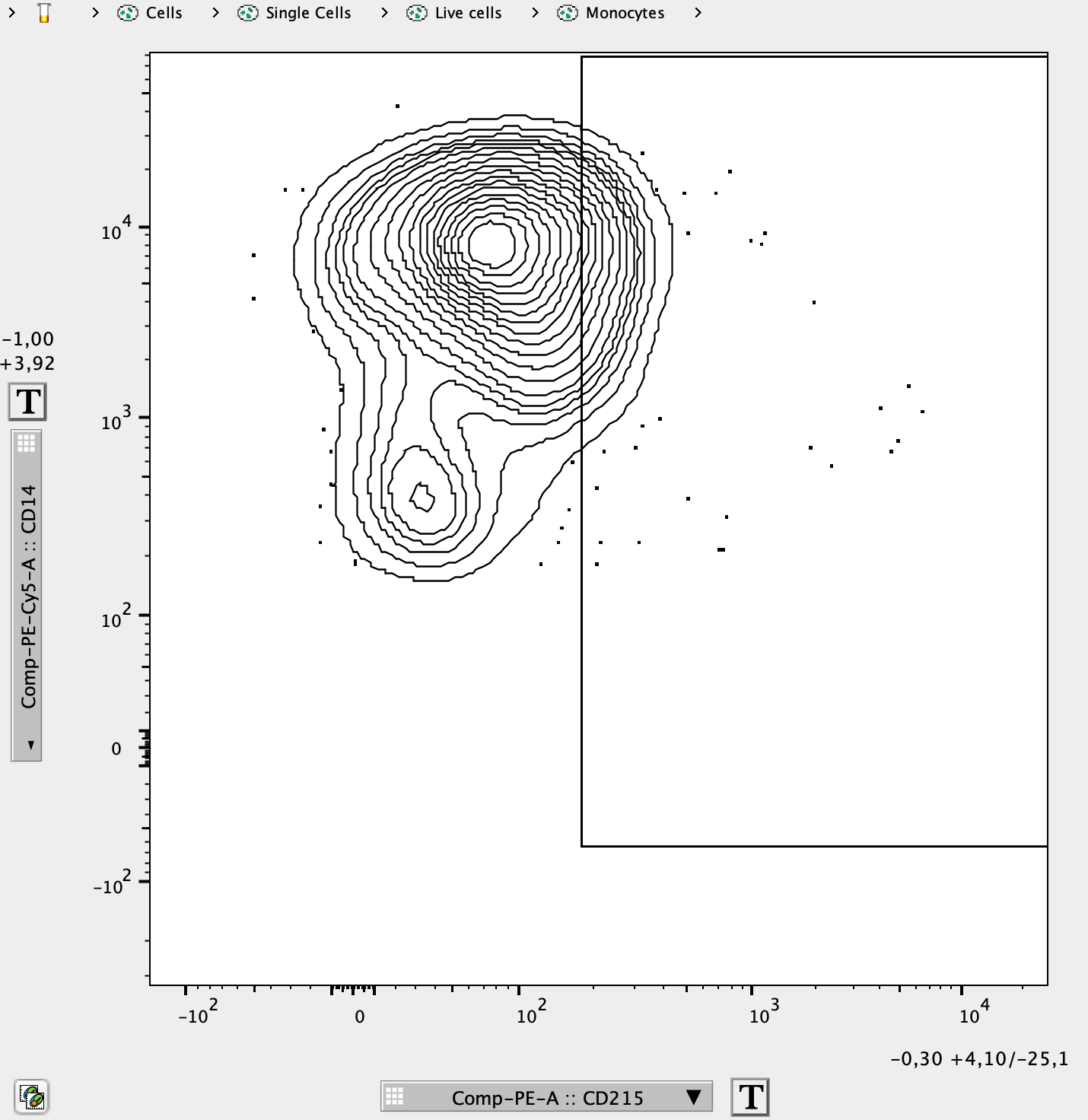

Supplement: Supplementary file 14 — Source Data for Figure 8 [file EMMM-15-e17694-s012.zip › Figure 8/8A/CD14 IL15Ra+/8A SCF d+9.tiff]

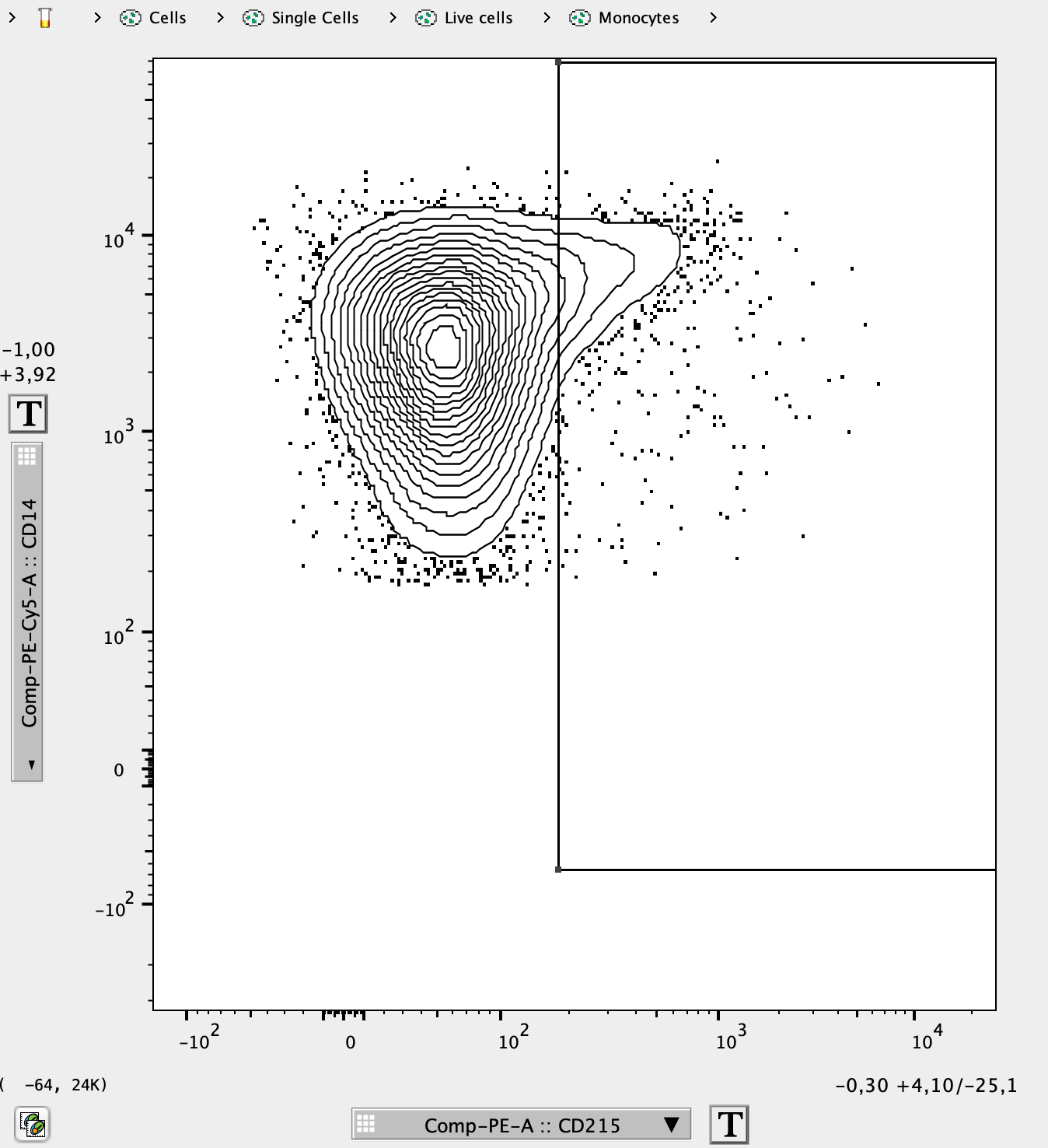

Supplement: Supplementary file 14 — Source Data for Figure 8 [file EMMM-15-e17694-s012.zip › Figure 8/8A/CD14 IL15Ra+/8A IL3 d+9.tiff]
