# Supplementary material for: M‐CSF directs myeloid and NK cell differentiation to protect from CMV after hematopoietic cell transplantation
Source: EMBO Mol Med. 2023 Aug 28;15(11):e17694. doi: 10.15252/emmm.202317694 (PMC10630876; doi:10.15252/emmm.202317694)
Supplement: Supplementary file 15 [file EMMM-15-e17694-s008.pdf]

EMBO Molecular Medicine

M-CSF directs myeloid and NK cell differentiation to protect from CMV after hematopoietic cell transplantation

Prashanth Kandalla, Julien Subburayalu, Clément Cocita, Bérengère de Laval, Elena Tomasello, Johanna Iacono, Jessica Nitsche, Maria Canali, Wilfried Cathou, Gilles Bessou, Noushin Mossadegh-Keller, Caroline Huber, Guy Mouchiroud, Roland Bourette, Marie-France Grasset, Martin Bornhäuser, Sandrine Sarrazin, Marc Dalod, and Michael Sieweke
DOI: 10.15252/emmm.202317694

Corresponding author: Michael Sieweke (michael.sieweke@tu-dresden.de)

Review Timeline:

Submission Date:	10th Mar 23
Editorial Decision:	14th Mar 23
Appeal:	17th Mar 23
Editor Correspondence:	28th Mar 23
Editorial Decision:	15th May 23
Revision Received:	23rd Jun 23
Editorial Decision:	13th Jul 23
Revision Received:	25th Jul 23
Accepted:	25th Jul 23

Editor: Zeljko Durdevic

Transaction Report:

(Note: With the exception of the correction of typographical or spelling errors that could be a source of ambiguity, letters and reports are not edited. Depending on transfer agreements, referee reports obtained elsewhere may or may not be included in this compilation. Referee reports are anonymous unless the Referee chooses to sign their reports.)

14th Mar 2023

Decision on your manuscript EMM-2023-17694

Dear Dr. Sieweke,

Thank you for the submission of your research manuscript to our editorial offices. I have now had the opportunity to read it and to discuss it with the other members of our editorial team. I am afraid we all agree that the manuscript is not well suited for publication in EMBO Molecular Medicine and have therefore decided not to proceed with its handling and peer review.

We do recognize potential interest of the study; however, previous studies, including from your laboratory, showing antimicrobial effects of M-CSF after hematopoietic cell transplantation (HCT), antimicrobial effects of myeloid progenitors after HCT as well as antiviral effects of G-CSF detract from the sort of conceptual advance we would expect in an EMBO Molecular Medicine article. Given these considerations, I am afraid that we cannot offer further consideration to your manuscript.

That being said, I discussed your work with Dr. Novella Guidi, scientific editor at our sister journal Life Science Alliance (www.life-science-alliance.org). Life Science Alliance is an open access journal launched in partnership between EMBO, Rockefeller, and Cold Spring Harbor Laboratory Presses, and it publishes work that is of high value to the respective communities across all areas in the life sciences. I am glad to say that Novella is interested in considering your work for publication and would like to send it out for formal peer-review at Life Science Alliance.

I am very sorry to disappoint you on this occasion and I hope you will view the encouragement of a transfer favorably. If this is the case, please use the link below to transfer the manuscript directly; no re-formatting is required.

Yours sincerely,

Zeljko Durdevic

Zeljko Durdevic
Editor
EMBO Molecular Medicine

==========================

As a service to authors, EMBO provides authors with the possibility to transfer a manuscript that one journal cannot offer to publish to another EMBO publication. The full manuscript and if applicable, reviewers reports are automatically sent to the receiving journal to allow for fast handling and a prompt decision on your manuscript. For more details of this service, and to transfer your manuscript to another EMBO title please click on Link Not Available

Please do not share this URL as it will give anyone who clicks it access to your account.

Dear Zeljko Durdevic

thank you for your swift response. Obviously we respect your editorial prerogatives in manuscript selection. Nevertheless I would like to argue with your reasoning for not sending the paper for review.

Indeed we showed previously that M-CSF during HCT has anti-bacterial and anti-fungal effects. However, by no means this could have predicted the anti-viral effects of M-CSF, since myeloid cells are not known to be the prime line of anti-viral defence. There also have been no reports, hints or indications that M-CSF could have such activity. This surprising effect that also puzzled ourselves initially could only be explained be the new mechanism of NK cell differentiation and activation initiated indirectly through Ill-15 by the M-CSF driven myelopoiesis that we revealed here. Therefore this study is by no means a simple embellishment or add-on to a previous study but stands out by itself and reveals a novel surprising mechanism of action.

We also would like to point out that we present human data in fig.7 that completely corroborate our findings in mice, something not done in previous studies, further underlining clinical relevance.

Furthermore G-CSF is completely ineffective as antiviral agent in HCT, as explained by the fact that it can only act on mature granulocytic cells, which are not present in the early phases of HCT, when patients are most vulnerable. Furthermore this effect on granulocytes can stimulate anti-bacterial but not antiviral activity.

Therefore there is both a novel mechanism and a clear clinical need, against which your points do not provide a counter argument. When presenting this story to clinicians in the transplantation field they have been thrilled to see M-CSF as a drug and expressed frustration that it is not available yet.

Therefore I continue to believe that EMBO MM would provide an excellent forum for clinicians and experimental scientists alike for this study that I believe will have major impact and generate a lot of attention and citations.
Although we will of course respect your final decision I would like to kindly urge you to weigh again our arguments and perhaps seek the advice of external reviewers to help in the decision process.

Best wishes

Michael Sieweke

Dear Dr. Sieweke,

Thank you for your response to the editorial decision on your manuscript entitled "M-CSF primes myeloid and NK cells to protect from CMV after hematopoietic stem cell transplantation". I have now carefully examined the arguments provided in your letter and discussed them with the other members of our editorial team. Additionally, I have sought external advice on the study from an expert in the field.

I am pleased to inform you that we decided to re-consider our initial decision and send your manuscript for peer review in EMBO Molecular Medicine.

Best wishes,
Zeljko Durdevic

15th May 2023

Dear Dr. Sieweke,

Thank you for the submission of your manuscript to EMBO Molecular Medicine, and please accept my apologies for the delay in getting back to you. We have received feedback from two of the three reviewers who agreed to evaluate your manuscript. Given that referee #2 will unfortunately not be able to return his/her report in a timely manner, we prefer to make a decision now in order to avoid further delay in the process. Should referee #2 provide a report, we will send it to you, with the understanding that we will not ask for an additional revision. As you will see from the reports below, both referees support publication of the study, but also raise important concerns that should be addressed in a revision of the current manuscript. If you would like to discuss further the points raised by the referees, I am available to do so via email or video. Let me know if you are interested in this option.

Addressing the reviewers' concerns in full will be necessary for further considering the manuscript in our journal. EMBO Molecular Medicine encourages a single round of revision only and therefore, acceptance or rejection of the manuscript will depend on the completeness of your responses included in the next, final version of the manuscript. For this reason, and to save you from any frustrations in the end, I would strongly advise against returning an incomplete revision.

We would welcome the submission of a revised version within three months for further consideration. Please let us know if you require longer to complete the revision.

I look forward to receiving your revised manuscript.

Yours sincerely,

Zeljko Durdevic

Zeljko Durdevic
Editor
EMBO Molecular Medicine

*** Instructions to submit your revised manuscript ***

*** PLEASE NOTE *** As part of the EMBO Publications transparent editorial process initiative (see our Editorial at https://www.embopress.org/doi/pdf/10.1002/emmm.201000094), EMBO Molecular Medicine will publish online a Review Process File to accompany accepted manuscripts.

In the event of acceptance, this file will be published in conjunction with your paper and will include the anonymous referee reports, your point-by-point response and all pertinent correspondence relating to the manuscript. If you do NOT want this file to be published, please inform the editorial office at contact@embomolmed.org.

When submitting your revised manuscript, please include:

1) a .docx formatted version of the manuscript text (including Figure legends and tables)

2) Separate figure files*

3) supplemental information as Expanded View and/or Appendix. Please carefully check the authors guidelines for formatting Expanded view and Appendix figures and tables at https://www.embopress.org/page/journal/17574684/authorguide#expandedview

4) a letter INCLUDING the reviewer's reports and your detailed responses to their comments (as Word

file).

5) The paper explained: EMBO Molecular Medicine articles are accompanied by a summary of the articles to emphasize the major findings in the paper and their medical implications for the non-specialist reader. Please provide a draft summary of your article highlighting
- the medical issue you are addressing,
- the results obtained and
- their clinical impact.
This may be edited to ensure that readers understand the significance and context of the research.
Please refer to any of our published articles for an example.

6) For more information: There is space at the end of each article to list relevant web links for further consultation by our readers. Could you identify some relevant ones and provide such information as well? Some examples are patient associations, relevant databases, OMIM/proteins/genes links, author's websites, etc...

7) Author contributions: the contribution of every author must be detailed in a separate section.

8) EMBO Molecular Medicine now requires a complete author checklist (https://www.embopress.org/page/journal/17574684/authorguide) to be submitted with all revised manuscripts. Please use the checklist as guideline for the sort of information we need WITHIN the manuscript. The checklist should only be filled with page numbers were the information can be found. This is particularly important for animal reporting, antibody dilutions (missing) and exact values and n that should be indicted instead of a range.

9) Every published paper now includes a 'Synopsis' to further enhance discoverability. Synopses are displayed on the journal webpage and are freely accessible to all readers. They include a short stand first (maximum of 300 characters, including space) as well as 2-5 one sentence bullet points that summarise the paper. Please write the bullet points to summarise the key NEW findings. They should be designed to be complementary to the abstract - i.e. not repeat the same text. We encourage inclusion of key acronyms and quantitative information (maximum of 30 words / bullet point). Please use the passive voice. Please attach these in a separate file or send them by email, we will incorporate them accordingly.

You are also welcome to suggest a striking image or visual abstract to illustrate your article. If you do please provide a jpeg file 550 px-wide x 400-px high.

10) A Conflict of Interest statement should be provided in the main text

11) Please note that we now mandate that all corresponding authors list an ORCID digital identifier. This takes <90 seconds to complete. We encourage all authors to supply an ORCID identifier, which will be linked to their name for unambiguous name identification.

Currently, our records indicate that the ORCID for your account is 0000-0002-3228-9537.

Please click the link below to modify this ORCID:
Link Not Available

12) The system will prompt you to fill in your funding and payment information. This will allow Wiley to send you a quote for the article processing charge (APC) in case of acceptance. This quote takes into account any reduction or fee waivers that you may be eligible for. Authors do not need to pay any fees before their manuscript is accepted and transferred to our publisher.

*Additional important information regarding Figures

Each figure should be given in a separate file and should have the following resolution:
Graphs 800-1,200 DPI
Photos 400-800 DPI
Colour (only CMYK) 300-400 DPI"

Figures are not edited by the production team. All lettering should be the same size and style; figure panels should be indicated by capital letters (A, B, C etc). Gridlines are not allowed except for log plots. Figures should be numbered in the order of their appearance in the text with Arabic numerals. Each Figure must have a separate legend and a caption is needed for each panel.

*Additional important information regarding figures and illustrations can be found at https://bit.ly/EMBOPressFigurePreparationGuideline. See also figure legend preparation guidelines: https://www.embopress.org/page/journal/17574684/authorguide#figureformat

The system will prompt you to fill in your funding and payment information. This will allow Wiley to send you a quote for the article processing charge (APC) in case of acceptance. This quote takes into account any reduction or fee waivers that you may be eligible for. Authors do not need to pay any fees before their manuscript is accepted and transferred to our publisher.

***** Reviewer's comments *****

Referee #1 (Comments on Novelty/Model System for Author):

Cytomegalovirus (CMV) infection remains a major threat in patients treated with hematopoietic stem cell transplantation and in other severe immunosuppressed conditions. The authors use a mouse model and murine CMV that was shown to recapitulate the human disease and provide attempts to validate their results in ex vivo cultures of human cells

Referee #1 (Remarks for Author):

The authors mostly use an in vivo mouse model of cytomegalovirus (CMV) infection to explore the protective effects of M-CSF and decipher the mechanisms involved. The objective is relevant as CMV infection (and some other viral infections) remains a major threat in immunocompromised patients, and pertinent as M-CSF stimulates the production of myeloid cells that are involved in the response to CMV infection.
While M-CSF injected at the time of hematopoietic stem cell transplantation (HSCT) has no adverse effect on donor cell engraftment and graft versus host disease, it protects >80% of animals from tissue damage and death induced by one dose of 5,000 cfu murine CMV (MCMV) injected at day 14 post-transplant. Using a variety of mouse models, M-CSF is shown to enhance reconstitution and development of monocytes and plasmacytoid dendritic cells. In turn, type 1 interferon, mostly released by pDCs, may activate IL-15 production by myeloid cells. IL-15 presentation by IL-15Rα on myeloid cells promotes the production of donor mature NK cells that accumulate at infectious foci in the liver and produce granzyme B and interferon-γ.
Finally, ex vivo experiments on human cells (peripheral blood mononucleated cells collected from G-CSF stimulated donors) exposed to stem cell factor and M-CSF confirms the stimulation of myelopoiesis with enhanced production of monocytes overexpressing IL-15Rα and functionally competent NK cells.
Overall, this is a well conducted and clearly reported study that enforces the previous demonstration of M-CSF ability to protect against bacterial and fungal infections and argues for re-introducing this growth factor in the therapeutic armamentarium used in severely immunosuppressed patients.

Suggestions to improve the manuscript
When G-CSF mobilized PBMCs are cultured ex vivo with M-CSF and SCF, monocytes are reported to express simultaneously CD14 and CD16 and designated as intermediate monocytes (IMs). It is very difficult to prevent the differentiation of ex vivo cultured human monocytes into monocyte-derived macrophages (Mo-Mac) and almost impossible to generate non-classical monocytes in these culture conditions. Could these cells be Mo-Mac rather that IMs?
Did the authors monitor the production of type 1 interferon, and possibly other inflammatory cytokines during MCMV infection of HSCT recipients?

Referee #3 (Comments on Novelty/Model System for Author):

The detailed examination of the effects of M-CSF on NK cells and on generation of IL-15 and different interferons both in vitro and in vivo are well done. The knockout model data are of particular value and interest. M-CSF has been largely overlooked by the transplant community. However, this paper, although about murine CMV and only a little about human CMV, could stimulate an important new direction.

Referee #3 (Remarks for Author):

This is an interesting paper on the effects of M-CSF on early reconstitution of NK cells and their precursors, as well as different subsets of monocytes, macrophages and dendritic cells and their function. The data presented on cytokines generated and on effects of M-CSF when administered to different KO mice are particularly interesting.

There are certain points that could further illustrate the significance of the data presented:
1. Most of the in vivo experiments provide survival data only through day 35 post M-CSF. Figure 3 appears to have longer follow-up. It would be important to know how long the mice were followed after day 35, at least in that experiment, so as to establish whether and to what degree effective resistance is persistent.
2. The data on GVHD are of critical importance, since concern regarding the potential of M-CSF to stimulate GVHD has historically inhibited consideration of M-CSF as an adjunct to enhance early recovery of innate and potentially adaptive immunity. However, it would be important to know to what degree M-CSF protects against viral infections when the alloresponsive T-cells begin to react against the host. In your model, the CMV challenge is administered at 2 weeks post

treatment with M-CSF and the transplant, but, as shown in Figure EV-5D by that time the alloresponse of T-cells inducing GVHD has almost completely resolved.

3. This paper is particularly focused on innate antiviral resistance induced by monocyte/macrophage populations their IL-15 and IL-15Rα products and their induction of NK cells. While it may be beyond the scope of the current report, do the authors have any information regarding resistance of the mice to secondary challenge with MCV?

Also, in the introduction and discussion, citation regarding experiment with murine CMV vs. human CMV should be clearly distinguish. In most instances, this has been done, but not all.

Revision and point-by-point response to “EMM-2023-17694-V2-Q”

Referee #1:

Point 1: […] Overall, this is a well conducted and clearly reported study that enforces the previous demonstration of M-CSF ability to protect against bacterial and fungal infections and argues for re-introducing this growth factor in the therapeutic armamentarium used in severely immunosuppressed patients.

Reply 1: We thank the reviewer for the positive evaluation of our manuscript and agree with the reviewer’s interest to re-introduce M-CSF as a general host-directed therapeutic for severe states of immunosuppression.

Point 2: Suggestions to improve the manuscript:

When G-CSF mobilized PBMCs are cultured ex vivo with M-CSF and SCF, monocytes are reported to express simultaneously CD14 and CD16 and designated as intermediate monocytes (IMs). It is very difficult to prevent the differentiation of ex vivo cultured human monocytes into monocyte-derived macrophages (Mo-Mac) and almost impossible to generate non-classical monocytes in these culture conditions. Could these cells be Mo-Mac rather than IMs?

Postadresse
Zentrum für Regenerative Therapien
TU Dresden
Fetscherstraße 105
01307 Dresden

Besucheradresse
Fetscherstraße 105
01307 Dresden

Zufahrt für
Rollstuhlfahrer
zum EG über Rampe
Haupteingang

Internet www.tu-dresden.de/crtd

Kein Zugang für elektronisch signierte sowie verschlüsselte elektronische Dokumente.

Mitglied von:

Reply 2: We appreciate and fully agree with the reviewer's comment. Indeed, our cytospins indicate macrophage rather than monocyte morphology at day 5 and day 9 of *in vitro* culture.

Accordingly, we have changed the terminology used in this manuscript to refer to *in vitro* cultured cells as being monocyte-derived macrophages (Mo-Macs). Moreover, this notion is also corroborated by re-analysis of our flow cytometric data (see panels B, I,J,K,L in the revised Fig. 7) that shows that compared to baseline (day 0), cells increase

- Fig. 7B: in granularity (SSC-A^{low} increases to SSC-$A^{intermediate}$) suggesting transition from monocytes to Mo-Macs,
- Fig. 7I: in numbers expressing the macrophage marker CD64 in $CD11b^{+}CD66b^{-}$ cells, suggesting transition from monocytes to Mo-Macs,
- Fig. 7J: in numbers expressing the macrophage marker HLA-DR in $CD11b^{+}CD66b^{-}$ cells, suggesting transition from monocytes to Mo-Macs.
- Fig. 7K/L: in numbers expressing $CD14^{hi}$ (Fig. 7K) and co-expressing CD16 on $CD14^{hi}$ cells, suggesting transition from monocytes to Mo-Macs.

Accordingly, we now indicate in **Fig. EV4C,** how $CD14^{+}CD16^{+}$ Mo-Macs were identified and removed the previous panel **E** in **Fig. 7**.

Point 3: Did the authors monitor the production of type 1 interferon, and possibly other inflammatory cytokines during MCMV infection of HSCT recipients?

Reply 3: We thank the reviewer for this question. Indeed, we have measured not only type I interferon (IFN) production such as IFN-β_1 (Ifnb1 shown in **Fig. 5C** by Fluidigm and **Fig. 6C** by FACS), but also the pro-inflammatory cytokines IL-15 (shown in **Fig. 5B** by qPCR), IFN-γ (shown in **Fig. 3A** by FACS and **Fig. 3C** by RT-qPCR), as well as Granzyme B (shown in **Fig. 3B** by FACS).

We would like to take the opportunity to thank reviewer 1 for the time and effort in reviewing our manuscript.

Referee #3:

Point 1: The detailed examination of the effects of M-CSF on NK cells and on generation of IL-15 and different interferons both in vitro and in vivo are well done. The knockout model data are of particular value and interest. M-CSF has been largely overlooked by the transplant community. However, this paper, although about murine CMV and only a little about human CMV, could stimulate an important new direction.

Reply 1: We are very grateful for the reviewer's appraisal of our data. We agree (and hope) that our manuscript highlights the surprising and general antimicrobial activities of M-CSF to render it a promising candidate for further pre-clinical and clinical studies regarding translatability and usability as a host-directed therapy to human disease in states of severe/acute leukocytopenia.

Point 2: There are certain points that could further illustrate the significance of the data presented:

1. Most of the in vivo experiments provide survival data only through day 35 post M-CSF. Figure 3 appears to have longer follow-up. It would be important to know how long the mice were followed after day 35, at least in the experiment, so as to establish whether and to what degree effective resistance is persistent.

   Reply 2.1: We thank the reviewer for this comment. Indeed, several mice were observed until day 50 without further signs of morbidity or mortality if the initial phase of MCMV viremia was survived (see Appendix Figure 1). We have added a statement to the "Materials and Methods" section to reflect this point:

   "For consistency, all survival analyses using Kaplan-Meier estimates pertaining to MCMV-infected mice are illustrated until 35 days post HSPC transplantation, although several mice were observed until day 50 without any further signs of morbidity and mortality."

   Accordingly, we are now showing the data consistently until day 35 in Fig. 1B, Fig. EV1A-D, Fig. 3E, Fig. 4C/E Fig. 5F/G, and Fig. 6D/E.

2. The data on GVHD are of critical importance, since concern regarding the potential of M-CSF to stimulate GVHD has historically inhibited consideration of M-CSF as an adjunct to enhance early recovery of innate and potentially adaptive immunity. However, it would be important to know to what degree M-CSF protects against viral infections when the alloresponsive T-cells begin to react against the host. In your model, the CMV challenge is administered at 2 weeks post treatment with M-CSF and the transplant, but, as shown in Figure EV-5D by that time the alloresponse of T-cells inducing GVHD has almost completely resolved.

   Reply 2.2: We thank the reviewer for this important point. All our HSPC transplants are T cell-depleted. As the reviewer rightly pointed out, it was important to test the effect of M-CSF application in an allotransplantation setting. Therefore, we transplanted C57BL/6j HSPCs into BALB/c mice. Here, a clinical scoring system established by Lai et al. (Cell Transplantation 2012) was used, which had six clinical criteria (weight loss, diarrhea, posture, activity, fur texture and skin integrity) with scores ranging from 0 (minimum) to 11 (maximum). A score of 3 was reached at day 10 (**maximum 11**), which appeared to be a mixture of irradiation sickness and challenging engraftment in this allogeneic setting (as compared to the previous congenic transplantations). To reflect this notion, we have changed the axis label in Fig. EV5D to "disease score".

Common allogeneic and xenogeneic transplantations in the murine setting whose transplants are T cell-depleted, require > 10-12 weeks for functional peripheral T cell engraftment (see for example Billerbeck et al., Blood 2011, PMID: 21252091; Alpdogan et al., J. Clin. Invest. 2003, PMID: 14523046). The expression of CD3 we measured at 4- and 12-weeks post transplantation is not sufficient to identify functional T cells, without measuring functional T cell receptors (CD4 or CD8, respectively) . It therefore remains unclear if these could be primed to become alloreactive.

The concern about potentially negative effects of M-CSF on GvH disease largely stems from a study by Alexander et al. (J. Clin. Invest. 2014, PMID: 25157821) that we have discussed in the revised version of the manuscript. It is important to note that also in this study M-CSF did not show any deleterious effect in T cell-depleted grafts (Fig. 9D, B6 T cell depleted (TCD) BM). We believe the **timing of M-CSF administration** is critical to instruct whether injurious or beneficial responses are incited. Indeed, Hashimoto et al. (J. Exp. Med. 2011, PMID: 21536742), who applied M-CSF shortly prior to the time of transplantation (comparable to our protocol) have identified M-CSF to ameliorate GvHD after allogeneic transplantation. For acute GvHD, M-CSF therefore appears to be beneficial. If engraftment is underway (beginning of normalization of leukocyte counts) such as is the case of the study reported by Alexander et al. (J. Clin. Invest. 2014, PMID: 25157821), continued administration of M-CSF for 5 days from day 14 after transplantation appeared to enhance chronic GvHD. Accordingly, we have slightly amended the discussion to make this distinction based on timing of administration more obvious.

In line with this**, M-CSF** given within 8 days of undergoing bone marrow transplantation (**aligning with our protocol**) was previously also **used safely in humans** who have undergone stem cell transplantation. Here, M-CSF could also develop a beneficial role to dampen GvHD and even chronic GvHD severity was reduced (Kimura et al., Bone Marrow Transplant. 2012, PMID: 21499320).

For the reviewer’s convenience, the revised section on GvHD is pasted here:

“Allogeneic HCT harbors the risk of acute and chronic GvHD (Ferrara *et al*, 2009). To date, most pre-clinical studies indicated a beneficial effect of M-CSF in allogeneic HCT. M-CSF treatment applied in mice just prior to transplantation induced host macrophages to engulf alloreactive T cells (Hashimoto *et al*, 2011). Conversely, depletion of resident macrophages by abrogating the M-CSF/CSF-1R-axis during the peri-transplantation period aggravated GvHD *in vivo* (MacDonald *et al*., 2010). With respect to chronic GvHD in the human setting, a retrospective analysis of over 50 Japanese bone marrow transplant recipients receiving M-CSF at or early after transplantation showed a lower risk to develop this complication (Kimura *et al*, 2012). This underpins the relevance of the timing of M-CSF administration for the differential effect on GvHD development. Although the early application of M-CSF peri-transplantation mediates the antimicrobial effects described by us and leads to a lower rate of chronic GvHD, it must be noted that the therapeutic window appears to be smaller when the compound is applied later after engraftment of T cell-replete allografts in mice (Alexander *et al*, 2014).”

3. This paper is particularly focused on innate antiviral resistance induced by monocyte/macrophage populations their IL-15 and IL-15Rα products and their induction of NK cells. While it may be beyond the scope of the current report, do the

authors have any information regarding resistance of the mice to secondary challenge with MCV?

Reply 2.3: We thank the reviewer for this excellent point. Indeed, we did not specifically perform this experiment. However, trained immunity (i.e., epigenetic memory in innate immune cells) can last for a significant period of time and is something we and others have observed to also occur on the stem cell level (see for example de Laval et al., Cell Stem Cell 2020, PMID: 32386557; Divanaghi et al., Nat. Immunol., 2021, PMID: 33293712). Inducers of trained immunity include mimetics of viral stimuli like polyI:C (see **Fig. 6** of de Laval et al., Cell Stem Cell 2020).

Furthermore, we have tested the effect of M-CSF on epigenetic changes in long-term HSCs treated once with 10 µg M-CSF i.v. and found that it can induce epigenetic changes in HSCs that lasted for at least 4 weeks (see appendix data below, **Fig. 2**). These changes include differences in OCR in genes relevant to myelopoiesis and genes relevant to the communication with the NK cell lineage (IL15, IFN-β). These data provide suggestive evidence that M-CSF stimulation in itself or in combination with viral infection would also lead to improved protection against secondary viral infection.

Point 3: Also, in the introduction and discussion, citation regarding experiment with murine CMV vs. human CMV should be clearly distinguish. In most instances, this has been done, but not all.

Reply 3: We thank the reviewer for this careful vigilance. We have now clearly distinguished references describing human from murine data by specifically indicating when studies were done in the mouse (indicated as "murine" or "*in vivo*"). Accordingly, we thank the reviewer for highlighting this issue and have amended several sections as follows:

- L. 746f.: "[…] morbidity and mortality as shown in mice (Arber et al., 2003)."
- L. 774: "We showed before in murine models that M-CSF […]"
- L. 787: "[…] we showed previously in mice *in vivo* that M-CSF but not G-CSF […]"
- L. 789f.: "Importantly, in previous murine studies M-CSF-induced myelopoiesis […]"
- L. 793: "[…] of M-CSF treatment *in vivo* by […]"
- L. 821f.: "[…] described both in mice (Alexandre et al., 2014; Brinkmann et al., 2015) as well as humans (Brinkmann et al., 2015)."
- L. 836: "On the one hand, rodent studies have shown […]"
- L. 839f.: "[…] macrophage depletion *in vivo* increased […]"
- L. 843: "[…] are involved in CMV dissemination in the mouse (Daley-Bauer et al., 2014) […]"
- L. 846f.: "In murine models, $Ly6C^{+}CCR2^{+}$ inflammatory monocytes [...]"
- L. 850: "[…] via MCP-1/CCL2 *in vivo* (Salazar-Mather et al., 2002)."
- L. 854: "Previously, murine studies showed that […]"
- L. 905: "[…] NK cell activation demonstrated in murine studies (Lucas et al., 2007; Nguyen et al., 2002), […]"
- L. 919f.: "[…] suggested for SARS-CoV2 in humans (Kandikattu et al., 2020)."

We would like to appreciate the excellent questions of reviewer 3 and to thank for the time and effort in reviewing our manuscript.

We thank the reviewers and the editorial office for the high quality of the peer review and hope to have now provided a revised manuscript suitable for publication in *EMBO Molecular Medicine*.

Yours sincerely on behalf of all the authors,

Michael Sieweke

Appendix data

Figure 1 Persistence of the M-CSF-induced survival effect > 35 days

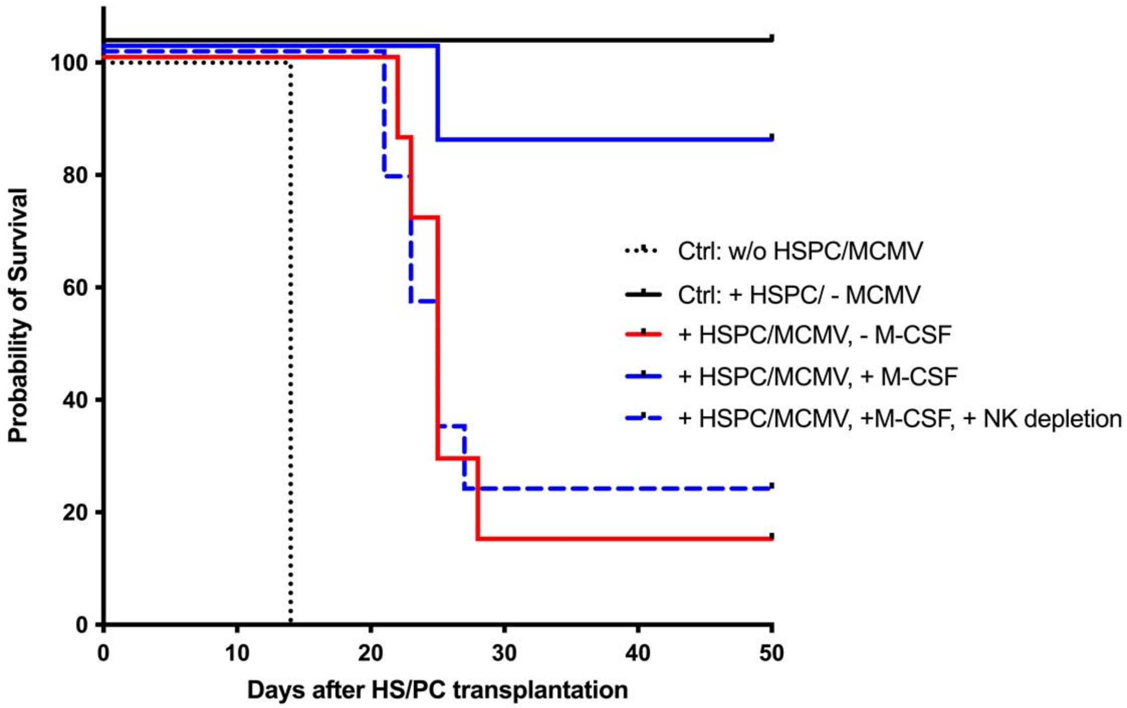

Mice were observed and assessed for their survival after HSPC transplantation followed by MCMV infection 14 days post HSPC transplantation. Mice that received M-CSF in accordance with the study protocol shown in Fig. 1A and that had survived the initial phase of MCMV-induced viremia showed no further signs of morbidity or mortality. The experiment was terminated arbitrarily at 50 days.

Figure 2 M-CSF induces epigenetic changes in long-term HSCs *in vivo*

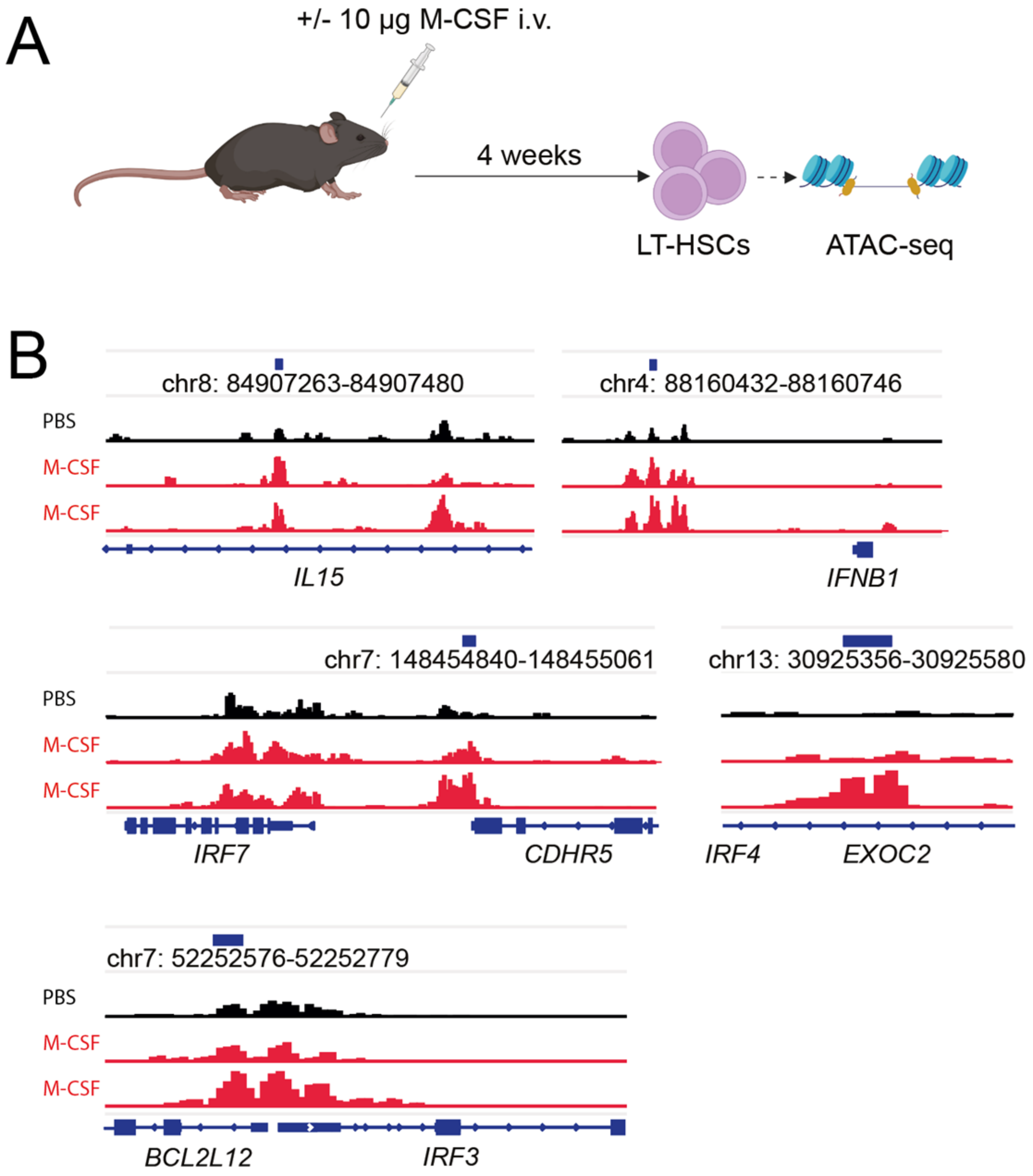

Epigenetic changes in long-term HSCs 4 weeks after one-time of *in vivo* treatment with 10 µg M-CSF i.v. are shown. LT-HSCs were sampled from the bone marrow 4 weeks after M-CSF treatment and underwent ATAC-seq. Genes for NK cell support/differentiation by the myeloid lineage as well as genes of the IRF family were found to be more accessible in response to *in vivo* M-CSF stimulation.

Point-by-point response to “EMM-2023-17694-V3 Initial Quality Check”

Editorial office 1: Figure 1G and figure 3D – we have detected a potential reuse of image which is not stated in the figure legend.

Reply 1: We apologize for the reuse of the same image, which went undetected by us and was due to miscommunication between investigators in the process of figure preparation. Thank you for detecting this. We have resolved this issue by removing the previous Fig. 1G, which is not required for the argument we make (reduced viral load). We and have amended the result sections accordingly:
“M-CSF-treated mice also showed a decreased viral load as shown be reduced numbers of infected hepatocytes (Fig. 1F) and viral RNA copy numbers (Fig. 1G).”
Accordingly, the figure legend was amended. We have deleted the text from previous 1G and have moved 1H to the 1G legend.

The images shown in Figure 3D correctly represent the data and remain unchanged. They provide additional information, since they also indicate the co-localization of reduced viral protein with NK cell-enriched areas.

Editorial office 2: Figure EV4C and E and two images within Figure EV4D – we have detected similar images within the data set.

Reply 2: In our view this does not comprise data reuse. This is a panel to describe the flow cytometric gating strategy. In Fig. EV4C we focus on the “Monocyte” gate and in Fig. EV4E we show the modified strategy published as OMIP-027 by focusing on Lin^- cells. Indeed, it is the same dot plot, however, focusing on a different population. Nevertheless, we have revised the figure legend of Fig. EV4E to stress this point and state the same plot is reused:
“The gating strategy of OMIP-027 with minor modifications <u>by gating on the Lin^- cells from the dot plot shown in C</u>.”

There is no data duplication or image reuse in Fig. EV4D. The plots might look similar but all dot plots are from separate populations of cells as indicated by the arrows shown in Fig. EV4C. Therefore, no changes were made.

Again, we apologise for the inconvenience caused and would like to thank the editorial office for making us aware of these errors. We hope to have revised our manuscript to the Editorial offices’ satisfaction allowing for re-review by the previous reviewers.

13th Jul 2023

Dear Dr. Sieweke,

Thank you for the submission of your revised manuscript to EMBO Molecular Medicine. I am pleased to inform you that we will be able to accept your manuscript pending the following final amendments:

1) In the main manuscript file, please do the following:
- Correct/answer the track changes suggested by our data editors by working from the attached document.
- Remove all track changes in the file and figure legend headings from the result section.
- Remove data not shown (p. 11).
- In M&M, statistical paragraph should reflect all information that you have filled in the Authors Checklist, especially regarding randomization, blinding, replication.
- Author contributions: Please remove it from the manuscript and specify author contributions in our submission system. CRediT has replaced the traditional author contributions section because it offers a systematic machine-readable author contributions format that allows for more effective research assessment. You are encouraged to use the free text boxes beneath each contributing author's name to add specific details on the author's contribution. More information is available in our guide to authors:
https://www.embopress.org/page/journal/17574684/authorguide#authorshipguidelines
- Please place following sections after "Materials and Methods" in the subsequent order: data availability section, acknowledgments, disclosure statement and competing interests, references, main figure legends, expanded figure legends.
- Please rename "Data and materials availability" to "Data availability" and remove the sentence "For original data, please contact michael.sieweke@tu-dresden.de".
- Correct the reference citation in the reference list. Where there are more than 10 authors on a paper, 10 will be listed, followed by "et al.". Please check "Author Guidelines" for more information.
https://www.embopress.org/page/journal/17574684/authorguide#referencesformat
2) Tables: Please remove all EV tables and their legends from the main manuscript and upload them together with their legends as separate files.
3) Source data: At EMBO Press, we ask the authors to provide the source data that were used to generate the main figures. My colleague Hannah Sonntag will contact you with guidance for preparing the submission of source data files.
4) Synopsis:
- Synopsis image: Please adjust the size of the synopsis image to 550 px-wide x (250-400)-px high and submit it as a high-resolution jpeg file.
- Please check your synopsis text and image before submission with your revised manuscript. Please be aware that in the proof stage minor corrections only are allowed (e.g., typos).
5) For more information: This space should be used to list relevant web links for further consultation by our readers. Could you identify some relevant ones and provide such information as well? Some examples are patient associations, relevant databases, OMIM/proteins/genes links, author's websites, etc...
6) As part of the EMBO Publications transparent editorial process initiative (see our Editorial at http://embomolmed.embopress.org/content/2/9/329), EMBO Molecular Medicine will publish online a Review Process File (RPF) to accompany accepted manuscripts. This file will be published in conjunction with your paper and will include the anonymous referee reports, your point-by-point response and all pertinent correspondence relating to the manuscript. Let us know whether you agree with the publication of the RPF and as here, if you want to remove or not any figures from it prior to publication. Please note that the Authors checklist will be published at the end of the RPF.
7) Please provide a point-by-point letter INCLUDING my comments as well as the reviewer's reports and your detailed responses (as Word file).

I look forward to reading a new revised version of your manuscript as soon as possible.

Yours sincerely,

Zeljko Durdevic

Zeljko Durdevic
Editor
EMBO Molecular Medicine

*** Instructions to submit your revised manuscript ***

*** PLEASE NOTE *** As part of the EMBO Publications transparent editorial process initiative (see our Editorial at https://www.embopress.org/doi/pdf/10.1002/emmm.201000094), EMBO Molecular Medicine will publish online a Review Process File to accompany accepted manuscripts.

In the event of acceptance, this file will be published in conjunction with your paper and will include the anonymous referee reports, your point-by-point response and all pertinent correspondence relating to the manuscript. If you do NOT want this file to be published, please inform the editorial office at contact@embomolmed.org.

When submitting your revised manuscript, please include:

1) a .docx formatted version of the manuscript text (including Figure legends and tables)

2) Separate figure files*

3) supplemental information as Expanded View and/or Appendix. Please carefully check the authors guidelines for formatting Expanded view and Appendix figures and tables at https://www.embopress.org/page/journal/17574684/authorguide#expandedview

4) a letter INCLUDING the reviewer's reports and your detailed responses to their comments (as Word file).

5) The paper explained: EMBO Molecular Medicine articles are accompanied by a summary of the articles to emphasize the major findings in the paper and their medical implications for the non-specialist reader. Please provide a draft summary of your article highlighting
- the medical issue you are addressing,
- the results obtained and
- their clinical impact.
This may be edited to ensure that readers understand the significance and context of the research.
Please refer to any of our published articles for an example.

6) For more information: There is space at the end of each article to list relevant web links for further consultation by our readers. Could you identify some relevant ones and provide such information as well? Some examples are patient associations, relevant databases, OMIM/proteins/genes links, author's websites, etc...

7) Author contributions: the contribution of every author must be detailed in a separate section.

8) EMBO Molecular Medicine now requires a complete author checklist (https://www.embopress.org/page/journal/17574684/authorguide) to be submitted with all revised manuscripts. Please use the checklist as guideline for the sort of information we need WITHIN the manuscript. The checklist should only be filled with page numbers were the information can be found. This is particularly important for animal reporting, antibody dilutions (missing) and exact values and n that should be indicted instead of a range.

9) Every published paper now includes a 'Synopsis' to further enhance discoverability. Synopses are displayed on the journal webpage and are freely accessible to all readers. They include a short stand first (maximum of 300 characters, including space) as well as 2-5 one sentence bullet points that summarise the paper. Please write the bullet points to summarise the key NEW findings. They should be designed to be complementary to the abstract - i.e. not repeat the same text. We encourage inclusion of key acronyms and quantitative information (maximum of 30 words / bullet point). Please use the passive voice. Please attach these in a separate file or send them by email, we will incorporate them accordingly.

You are also welcome to suggest a striking image or visual abstract to illustrate your article. If you do please provide a jpeg file 550 px-wide x 400-px high.

10) A Conflict of Interest statement should be provided in the main text

11) Please note that we now mandate that all corresponding authors list an ORCID digital identifier. This takes <90 seconds to complete. We encourage all authors to supply an ORCID identifier, which will be linked to their name for unambiguous name

identification.

Currently, our records indicate that the ORCID for your account is 0000-0002-3228-9537.

Please click the link below to modify this ORCID:
Link Not Available

12) The system will prompt you to fill in your funding and payment information. This will allow Wiley to send you a quote for the article processing charge (APC) in case of acceptance. This quote takes into account any reduction or fee waivers that you may be eligible for. Authors do not need to pay any fees before their manuscript is accepted and transferred to our publisher.

*Additional important information regarding Figures

Each figure should be given in a separate file and should have the following resolution:
Graphs 800-1,200 DPI
Photos 400-800 DPI
Colour (only CMYK) 300-400 DPI"

Figures are not edited by the production team. All lettering should be the same size and style; figure panels should be indicated by capital letters (A, B, C etc). Gridlines are not allowed except for log plots. Figures should be numbered in the order of their appearance in the text with Arabic numerals. Each Figure must have a separate legend and a caption is needed for each panel.

*Additional important information regarding figures and illustrations can be found at https://bit.ly/EMBOPressFigurePreparationGuideline. See also figure legend preparation guidelines: https://www.embopress.org/page/journal/17574684/authorguide#figureformat

The system will prompt you to fill in your funding and payment information. This will allow Wiley to send you a quote for the article processing charge (APC) in case of acceptance. This quote takes into account any reduction or fee waivers that you may be eligible for. Authors do not need to pay any fees before their manuscript is accepted and transferred to our publisher.

***** Reviewer's comments *****

Referee #1 (Remarks for Author):

The authors adequately addressed the suggestions made in the initial review. No additional suggestion to improve this very interesting manuscript

Referee #3 (Remarks for Author):

Thank you for addressing each of my critiques adequately. It is a highly informative paper.

The authors addressed the minor editorial changes.

Referee #1:

Point 1: The authors adequately addressed the suggestions made in the initial review. No additional suggestion to improve this very interesting manuscript.

Reply 1: We thank the reviewer for the positive evaluation of our manuscript and would like to acknowledge the time commitment and insightful comments of Referee #1 in reviewing our manuscript.

Referee #3:

Point 1: Thank you for addressing each of my critiques adequately. It is a highly informative paper.

Reply 1: We thank the reviewer for the positive evaluation of our manuscript and would like to acknowledge the time commitment and valuable comments of Referee #3 in reviewing our manuscript.

25th Jul 2023

Dear Dr. Sieweke,

We are pleased to inform you that your manuscript is accepted for publication and is now being sent to our publisher to be included in the next available issue of EMBO Molecular Medicine.

Please read below for additional IMPORTANT information regarding your article, its publication and the production process.

Congratulations on your interesting work,

Zeljko Durdevic

Zeljko Durdevic
Editor
EMBO Molecular Medicine

Follow us on Twitter @EmboMolMed
Sign up for eTOCs at embopress.org/alertsfeeds

*** *** *** IMPORTANT INFORMATION *** *** ***

SPEED OF PUBLICATION
The journal aims for rapid publication of papers, using using the advance online publication "Early View" to expedite the process: A properly copy-edited and formatted version will be published as "Early View" after the proofs have been corrected. Please help the Editors and publisher avoid delays by providing e-mail address(es), telephone and fax numbers at which author(s) can be contacted.

Should you be planning a Press Release on your article, please get in contact with embomolmed@wiley.com as early as possible, in order to coordinate publication and release dates.

LICENSE AND PAYMENT:

All articles published in EMBO Molecular Medicine are fully open access: immediately and freely available to read, download and share.

EMBO Molecular Medicine charges an article processing charge (APC) to cover the publication costs. You, as the corresponding author for this manuscript, should have already received a quote with the article processing fee separately. Please let us know in case this quote has not been received.

Once your article is at Wiley for editorial production you will receive an email from Wiley's Author Services system, which will ask you to log in and will present you with the publication license form for completion. Within the same system the publication fee can be paid by credit card, an invoice, pro forma invoice or purchase order can be requested.

Payment of the publication charge and the signed Open Access Agreement form must be received before the article can be published online.

PROOFS

You will receive the proofs by e-mail approximately 2 weeks after all relevant files have been sent o our Production Office. Please return them within 48 hours and if there should be any problems, please contact the production office at embopressproduction@wiley.com.

Please inform us if there is likely to be any difficulty in reaching you at the above address at that time. Failure to meet our

deadlines may result in a delay of publication.

All further communications concerning your paper proofs should quote reference number EMM-2023-17694-V4 and be directed to the production office at embopressproduction@wiley.com.

Thank you,

Zeljko Durdevic
Editor
EMBO Molecular Medicine

EMBO Press Author Checklist

Corresponding Author Name: Michael H. Sieweke	
Journal Submitted to: EMBO Molecular Medicine	
Manuscript Number: EMM-2023-17694-V2-Q	

USEFUL LINKS FOR COMPLETING THIS FORM
The EMBO Journal - Author Guidelines
EMBO Reports - Author Guidelines
Molecular Systems Biology - Author Guidelines
EMBO Molecular Medicine - Author Guidelines

Reporting Checklist for Life Science Articles (updated January
This checklist is adapted from Materials Design Analysis Reporting (MDAR) Checklist for Authors. MDAR establishes a minimum set of requirements in transparent reporting in the life sciences (see Statement of Task: 10.31222/osf.io/9sm4x). Please follow the journal's guidelines in preparing your
Please note that a copy of this checklist will be published alongside your article.

Abridged guidelines for figures
1. Data
The data shown in figures should satisfy the following conditions:
- the data were obtained and processed according to the field's best practice and are presented to reflect the results of the experiments in an accurate and unbiased manner.
- ideally, figure panels should include only measurements that are directly comparable to each other and obtained with the same assay.
- plots include clearly labeled error bars for independent experiments and sample sizes. Unless justified, error bars should not be shown for technical
- if n<5, the individual data points from each experiment should be plotted. Any statistical test employed should be justified.
- Source Data should be included to report the data underlying figures according to the guidelines set out in the authorship guidelines on Data

2. Captions
Each figure caption should contain the following information, for each panel where they are relevant:
- a specification of the experimental system investigated (eg cell line, species name).
- the assay(s) and method(s) used to carry out the reported observations and measurements.
- an explicit mention of the biological and chemical entity(ies) that are being measured.
- an explicit mention of the biological and chemical entity(ies) that are altered/varied/perturbed in a controlled manner.
- the exact sample size (n) for each experimental group/condition, given as a number, not a range;
- a description of the sample collection allowing the reader to understand whether the samples represent technical or biological replicates (including how many animals, litters, cultures, etc.).
- a statement of how many times the experiment shown was independently replicated in the laboratory.
- definitions of statistical methods and measures:
  - common tests, such as t-test (please specify whether paired vs. unpaired), simple χ2 tests, Wilcoxon and Mann-Whitney tests, can be unambiguously identified by name only, but more complex techniques should be described in the methods section;
  - are tests one-sided or two-sided?
  - are there adjustments for multiple comparisons?
  - exact statistical test results, e.g., P values = x but not P values < x;
  - definition of 'center values' as median or average;
  - definition of error bars as s.d. or s.e.m.

Please complete ALL of the questions below.
Select "Not Applicable" only when the requested information is not relevant for your study.

Materials

Newly Created Materials	Information included in the manuscript?	In which section is the information available? (Reagents and Tools Table, Materials and Methods, Figures, Data Availability Section)
New materials and reagents need to be available; do any restrictions apply?		N/A

Antibodies	Information included in the manuscript?	In which section is the information available? (Reagents and Tools Table, Materials and Methods, Figures, Data Availability Section)
For **antibodies** provide the following information:<br>- Commercial antibodies: RRID (if possible) or supplier name, catalogue number and or/clone number<br>- Non-commercial: RRID or citation	Yes	Table EV1 (flow cytometry antibodies used), Table EV2 (immunofluorescent antibodies used)

DNA and RNA sequences	Information included in the manuscript?	In which section is the information available? (Reagents and Tools Table, Materials and Methods, Figures, Data Availability Section)
**Short novel DNA or RNA including primers, probes**: provide the sequences.	Yes	Table EV3 (information on primer sequences used)

Cell materials	Information included in the manuscript?	In which section is the information available? (Reagents and Tools Table, Materials and Methods, Figures, Data Availability Section)
**Cell lines:** Provide species information, strain. Provide accession number in repository **OR** supplier name, catalog number, clone number, and/**OR** RRID.	Not Applicable	N/A
**Primary cultures:** Provide species, strain, sex of origin, genetic modification status.	Yes	Human G-CSF-mobilized stem and progenitor cell/PBMCs (section Materials and Methods) sourced from the Department of Transfusion Medicine (TU Dresden)
Report if the cell lines were recently **authenticated** (e.g., by STR profiling) and tested for mycoplasma contamination.	Not Applicable	N/A

Experimental animals	Information included in the manuscript?	In which section is the information available? (Reagents and Tools Table, Materials and Methods, Figures, Data Availability Section)
**Laboratory animals or Model organisms:** Provide species, strain, sex, age, genetic modification status. Provide accession number in repository **OR** supplier name, catalog number, clone number, **OR** RRID.	Yes	Materials and Methods (Mice and in vivo treatments)
**Animal observed in or captured from the field:** Provide species, sex, and age where possible**.**	Not Applicable	N/A
Please detail **housing and husbandry conditions**.	Yes	Study approval section (Materials and Methods)

Plants and microbes	Information included in the manuscript?	In which section is the information available? (Reagents and Tools Table, Materials and Methods, Figures, Data Availability Section)
**Plants:** provide species and strain, ecotype and cultivar where relevant, unique accession number if available, and source (including location for collected wild specimens).	Not Applicable	N/A
**Microbes:** provide species and strain, unique accession number if available, and source.	Not Applicable	N/A

Human research participants	Information included in the manuscript?	In which section is the information available? (Reagents and Tools Table, Materials and Methods, Figures, Data Availability Section)
If collected and within the bounds of privacy constraints report on age, sex and gender or ethnicity for all study participants.	Yes	Healthy young volunteers (male/female) - age/sex/gender/ethnicity information not available to us - Materials and Methods

Core facilities	Information included in the manuscript?	In which section is the information available? (Reagents and Tools Table, Materials and Methods, Figures, Data Availability Section)
If your work benefited from core facilities, was their service mentioned in the acknowledgments section?	Yes	Acknowledgments

Design

Study protocol	Information included in the manuscript?	In which section is the information available? (Reagents and Tools Table, Materials and Methods, Figures, Data Availability Section)
If study protocol has been **pre-registered, provide DOI in the manuscript**. For clinical trials, provide the trial registration number **OR** cite DOI.	Not Applicable	N/A
Report the **clinical trial registration number** (at ClinicalTrials.gov or equivalent), where applicable.	Not Applicable	N/A

Laboratory protocol	Information included in the manuscript?	In which section is the information available? (Reagents and Tools Table, Materials and Methods, Figures, Data Availability Section)
Provide DOI OR other citation details if **external detailed step-by-step protocols** are available.	Not Applicable	N/A

Experimental study design and statistics	Information included in the manuscript?	In which section is the information available? (Reagents and Tools Table, Materials and Methods, Figures, Data Availability Section)
Include a statement about **sample size** estimate even if no statistical methods were used.	Yes	Statistical analysis
Were any steps taken to minimize the effects of subjective bias when allocating animals/samples to treatment (e.g. **randomization procedure**)? If yes, have they been described?	Yes	Mice and in vivo treatments
Include a statement about **blinding** even if no blinding was done.	Yes	MCMV infection, viral loads and histopathology
Describe **inclusion/exclusion criteria** if samples or animals were excluded from the analysis. Were the criteria pre-established?<br><br>If sample or data points were omitted from analysis, report if this was due to attrition or intentional exclusion and provide justification.	Yes	Statistical analysis
For every figure, are **statistical tests** justified as appropriate? Do the data meet the assumptions of the tests (e.g., normal distribution)? Describe any methods used to assess it. Is there an estimate of variation within each group of data? Is the variance similar between the groups that are being statistically compared?	Yes	Each figure caption.

Sample definition and in-laboratory replication	Information included in the manuscript?	In which section is the information available? (Reagents and Tools Table, Materials and Methods, Figures, Data Availability Section)
In the figure legends: state number of times the experiment was **replicated** in laboratory.	Yes	Each figure caption.
In the figure legends: define whether data describe **technical or biological replicates**.	Yes	Each figure caption.

Ethics

Ethics	Information included in the manuscript?	In which section is the information available? (Reagents and Tools Table, Materials and Methods, Figures, Data Availability Section)
Studies involving **human participants**: State details of **authority granting ethics approval** (IRB or equivalent committee(s), provide reference number for approval.	Yes	Study approval.
Studies involving **human participants**: Include a statement confirming that **informed consent** was obtained from all subjects and that the experiments conformed to the principles set out in the WMA Declaration of Helsinki and the Department of Health and Human Services Belmont Report.	Yes	Study approval.
Studies involving **human participants:** For publication of **patient photos**, include a statement confirming that consent to publish was obtained.	Not Applicable	N/A
Studies involving experimental **animals**: State details of **authority granting ethics approval** (IRB or equivalent committee(s), provide reference number for approval. Include a statement of compliance with ethical regulations.	Yes	Study approval.
Studies involving **specimen and field samples:** State if relevant **permits** obtained, provide details of authority approving study; if none were required, explain why.	Not Applicable	N/A

Dual Use Research of Concern (DURC)	Information included in the manuscript?	In which section is the information available? (Reagents and Tools Table, Materials and Methods, Figures, Data Availability Section)
Could your study fall under dual use research restrictions? Please check biosecurity documents and list of **select agents and toxins** (CDC): https://www.selectagents.gov/sat/list.htm	Not Applicable	N/A
If you used a select agent, is the security level of the lab appropriate and reported in the manuscript?	Not Applicable	N/A
If a study is subject to dual use research of concern regulations, is the name of the **authority granting approval and reference number** for the regulatory approval provided in the manuscript?	Not Applicable	N/A

Reporting

The MDAR framework recommends adoption of discipline-specific guidelines, established and endorsed through community initiatives. Journals have their own policy about requiring specific guidelines and recommendations to complement MDAR.

Adherence to community standards	Information included in the manuscript?	In which section is the information available? (Reagents and Tools Table, Materials and Methods, Figures, Data Availability Section)
State if relevant guidelines or checklists (e.g., **ICMJE, MIBBI, ARRIVE, PRISMA**) have been followed or provided.	Not Applicable	N/A
For **tumor marker prognostic studies,** we recommend that you follow the **REMARK** reporting guidelines (see link list at top right). See author guidelines, under 'Reporting Guidelines'. Please confirm you have followed these guidelines.	Not Applicable	N/A
For **phase II and III randomized controlled trials**, please refer to the **CONSORT** flow diagram (see link list at top right) and submit the CONSORT checklist (see link list at top right) with your submission. See author guidelines, under 'Reporting Guidelines'. Please confirm you have submitted this list.	Not Applicable	N/A

Data Availability

Data availability	Information included in the manuscript?	In which section is the information available? (Reagents and Tools Table, Materials and Methods, Figures, Data Availability Section)
Have **primary datasets** been deposited according to the journal's guidelines (see 'Data Deposition' section) and the respective accession numbers provided in the Data Availability Section?	Not Applicable	N/A
Were **human clinical and genomic datasets** deposited in a public access-controlled repository in accordance to ethical obligations to the patients and to the applicable consent agreement?	Not Applicable	N/A
Are **computational models** that are central and integral to a study available without restrictions in a machine-readable form? Were the relevant accession numbers or links provided?	Not Applicable	N/A
If publicly available data were reused, provide the respective **data citations in the reference list**.	Not Applicable	N/A
