# Supplementary material for: M‐CSF directs myeloid and NK cell differentiation to protect from CMV after hematopoietic cell transplantation
Source: EMBO Mol Med. 2023 Aug 28;15(11):e17694. doi: 10.15252/emmm.202317694 (PMC10630876; doi:10.15252/emmm.202317694)

BD FACSDiva 9.0.1

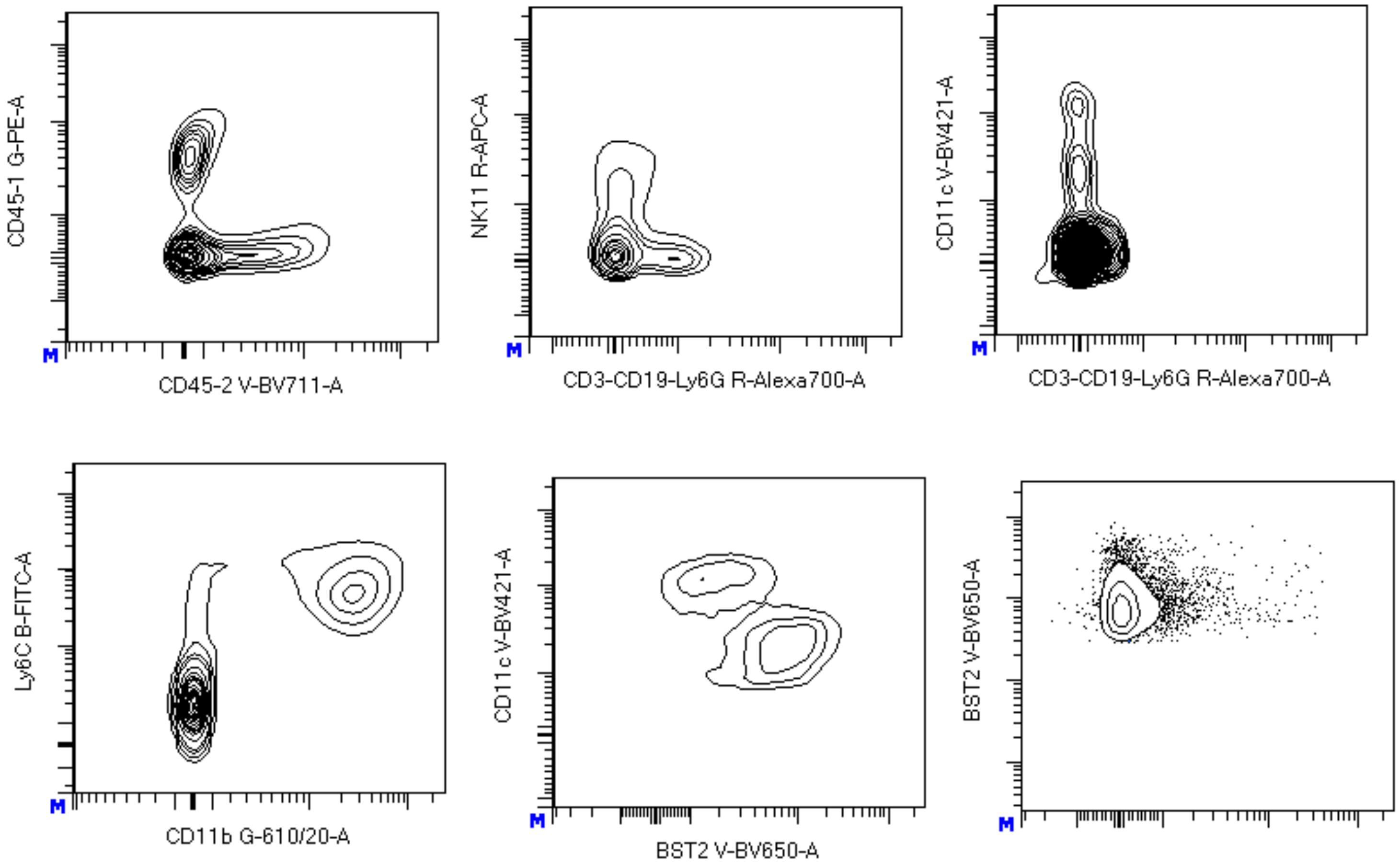

Supplement: Supplementary file 5 — Source Data for Expanded View [file EMMM-15-e17694-s006.zip › Figure EV3.zip!/Figure EV3/EV3C/EV3C FACS DIVA.tiff]
